# Univariate and Multivariate QTL Analyses Reveal Covariance Among Mineral Elements in the Rice Ionome

**DOI:** 10.3389/fgene.2021.638555

**Published:** 2021-01-25

**Authors:** Huan Liu, Su-Xian Long, Shannon R. M. Pinson, Zhong Tang, Mary Lou Guerinot, David E. Salt, Fang-Jie Zhao, Xin-Yuan Huang

**Affiliations:** ^1^State Key Laboratory of Crop Genetics and Germplasm Enhancement, College of Resources and Environmental Sciences, Nanjing Agricultural University, Nanjing, China; ^2^USDA–ARS Dale Bumpers National Rice Research Center, Stuttgart, AR, United States; ^3^Department of Biological Sciences, Dartmouth College, Hanover, NH, United States; ^4^Future Food Beacon of Excellence and the School of Biosciences, University of Nottingham, Loughborough, United Kingdom

**Keywords:** Rice, ionome, mineral nutrient, QTL, principal component analysis

## Abstract

Rice provides more than one fifth of daily calories for half of the world’s human population, and is a major dietary source of both essential mineral nutrients and toxic elements. Rice grains are generally poor in some essential nutrients but may contain unsafe levels of some toxic elements under certain conditions. Identification of quantitative trait loci (QTLs) controlling the concentrations of mineral nutrients and toxic trace metals (the ionome) in rice will facilitate development of nutritionally improved rice varieties. However, QTL analyses have traditionally considered each element separately without considering their interrelatedness. In this study, we performed principal component analysis (PCA) and multivariate QTL analyses to identify the genetic loci controlling the covariance among mineral elements in the rice ionome. We resequenced the whole genomes of a rice recombinant inbred line (RIL) population, and performed univariate and multivariate QTL analyses for the concentrations of 16 elements in grains, shoots and roots of the RIL population grown in different conditions. We identified a total of 167 unique elemental QTLs based on analyses of individual elemental concentrations as separate traits, 53 QTLs controlling covariance among elemental concentrations within a single environment/tissue (PC-QTLs), and 152 QTLs which determined covariation among elements across environments/tissues (aPC-QTLs). The candidate genes underlying the QTL clusters with elemental QTLs, PC-QTLs and aPC-QTLs co-localized were identified, including *OsHMA4* and *OsNRAMP5*. The identification of both elemental QTLs and PC QTLs will facilitate the cloning of underlying causal genes and the dissection of the complex regulation of the ionome in rice.

## Introduction

Rice provides more than one fifth of daily calories for half of the world’s human population, making it a staple food crop of primary global importance. In addition to being the main calorie source, rice can also be a major dietary source of both essential mineral nutrients and toxic elements. It was estimated that more than 60%, 30% and 15% of the world population suffer from deficiencies of iron (Fe), zinc (Zn) and selenium (Se), respectively (White and Broadley, 2009). Deficiencies of calcium (Ca), magnesium (Mg) and copper (Cu) are also increasingly common in many countries (Frossard et al., 2000; Rude and Gruber, 2004; Welch and Graham, 2005; Thacher et al., 2006). Hidden hunger, caused by diets deficient for essential vitamins and mineral nutrients, has become a global challenge. On the other hand, rice can also be a major dietary source of toxic heavy metals, especially for persons consuming rice as their staple food. For example, rice was found to contribute as much as 56 and 60% of total dietary intake of two toxic elements cadmium (Cd) and arsenic (As), respectively, for the general population in China (Li et al., 2011; Song et al., 2017). Therefore, it is desirable to increase the levels of health-beneficial elements and to decrease the accumulation of toxic elements in rice grains to ensure the production of nutritious and safe rice.

Breeding rice varieties that produce grains enriched for mineral nutrients and reduced in toxic elements offers a cost−effective strategy to alleviate micronutrient malnutrition and ensure grain nutritional quality. Identification of QTLs or genes that control the concentrations of essential mineral nutrients and toxic trace metals (also known as the ionome) in rice grains can increase the speed and efficiency of the variety improvement process. In the past few decades, many efforts have been made in mapping QTLs regulating the rice grain ionome, and hundreds of genetic loci have been identified for individual elements through bi-parental (Norton et al., 2010, 2019; Zhang et al., 2014; Yu et al., 2015; Chen et al., 2019; Raza et al., 2019; Liu et al., 2020; Wang et al., 2020) or genome wide association (GWA) mapping approaches (Norton et al., 2014; Huang et al., 2015; Yang et al., 2018; Tan et al., 2020). Among these QTLs, only a small number of causal genes have been identified and functionally characterized, including *OsHMA3*, *OsCAL1* and *OsCd1* for Cd (Ueno et al., 2010; Miyadate et al., 2011; Luo et al., 2018; Sui et al., 2019; Yan et al., 2019), *OsHMA4* for Cu (Huang et al., 2016), and *OsMOT1;1* for molybdenum (Mo) (Yang et al., 2018; Huang et al., 2019; Wang et al., 2020). This limited progress in characterizing the molecular basis of most of these ionomic QTLs is due in part to the generally small genetic contribution to the overall variation in the phenotype.

Quantitative trait loci (QTL) or GWA mapping analyses have traditionally been performed on individual elements. The ionome was simply treated as a collection of multiple independent elements and each element was analyzed individually. However, there is growing evidence showing that the elements in the ionome do not behave independently, suggesting that multiple elements should be combined into one or more multi-element phenotypes of interest (see review Baxter, 2015). Covariation among multiple elements in the ionome could be due to poor specificity of transporters allowing them to move more than one element across membranes, as well as alterations of plant physiological properties such as root structure or transpiration rates that would impact the uptake or transport of multiple elements (Baxter, 2015). Therefore, it is important to consider combinations of multiple elements in the ionome and to treat the covariation among multiple elements as a unique trait when carrying out genetic analyses. Including consideration of element interrelatedness and multi-element combinations provides additional information for better dissecting and understanding the complex ionomic regulation network, but also increases the power to detect genetic loci that control the covariation among multiple elements which are likely not detectable by the analysis of elements as separate and independent traits.

Multivariate analysis methods can be used to study multiple correlated phenotypes as observed in the ionome with multiple covarying elements. Principal components analysis (PCA) is an effective method for extracting key information from multiple correlated traits (Ringner, 2008). PCA can reduce the dimensionality of data by transforming multiple correlated variables into a smaller set of independent variables as principal components (PCs) without losing much of the variation of the original dataset. The PCs can be treated as composite traits and subjected to QTL analyses or GWA mapping. Several previous studies have shown that PCs were useful for the dissection of genetic networks controlling a variety of complex traits, including body weight of postnatal growth in mice (Ishikawa and Namikawa, 2004), plant architecture and kernel related traits in maize (Zhang et al., 2010; Choe and Rocheford, 2012; Liu et al., 2016; Bouchet et al., 2017; Fikas et al., 2019), and root system and above ground architecture in rice (Topp et al., 2013; Yano et al., 2019). Recently, GWA mapping for rice architecture using PC scores as traits have identified several genes regulating plant architecture (Yano et al., 2019). These studies suggest that the use of PCA in QTL analyses or GWA mapping can facilitate identification of genes controlling complex traits comprising multiple correlated variables.

In this study, we have performed PCA and QTL mapping on the ionome in different tissues of a rice recombinant inbred population grown under different conditions. QTL analyses have previously been performed on grain ionome of the recombinant inbred lines (LT-RILs) derived from a cross from Lemont (LM) and TeQing (TQ) grown under flooded conditions using 176 restriction fragment length polymorphism (RFLP) markers (Zhang et al., 2014). Due to the small number of markers being used and the QTL analyses being performed only on one tissue (grain) under one growth condition (flooded), limited number of QTLs with relatively large mapping intervals were identified. To capture more elemental QTLs in different tissue or under different growth conditions, as well as the QTLs controlling the covariation of elemental concentrations within an individual growing environment/tissue or across environments/tissues, we have performed univariate QTL analyses on the concentrations of 16 elements in three tissues (grains, shoots and roots) and under four different conditions (flooded, unflooded, semi-flooded and hydroponic) as well as multivariate QTL analyses by using the PC scores with individual growth environment/tissue or across environments/tissues as traits. The identification of both elemental QTLs and PC QTLs in this study not only improves opportunity to breed for, clone, or otherwise study the genes that enhance the nutritional quality of rice grains, but also shines light on the dissection of the complex regulation of the ionome in rice.

## Results

### Genotyping LT-RILs by Whole Genome Resequencing and Bin Map Construction

The Lemont-TeQing recombinant inbred population (LT-RILs), derived from a cross between a tropical *japonica* rice cultivar Lemont (LM) and an *indica* cultivar TeQing (TQ), has been previously genotyped by 176 RFLP markers (Tabien et al., 2000), and used for several QTL mapping studies, including discovery of QTLs for the rice grain ionome (Tabien et al., 2002; Pinson et al., 2005, 2010; Zhang et al., 2014). To increase the power and the precision of QTL mapping, we genotyped the RIL population and two parents by whole genome resequencing. The LT-RIL population being resequenced was at the F_20_ generation. A total of 241.84 Gb of sequencing data were generated for the two parents and 257 RILs with more than 96% of clean reads being mapped to the Nipponbare reference genome (Supplementary Table 1). LM and TQ were sequenced at 65 × and 57 × coverage, respectively, while the population was sequenced at 2 × coverage on average for each RIL. In the parental lines, more than 92% of genome region was covered at a minimum of 1 × sequencing depth, and the RILs had on average 64% of genome coverage (Supplementary Table 1).

**FIGURE 1:**
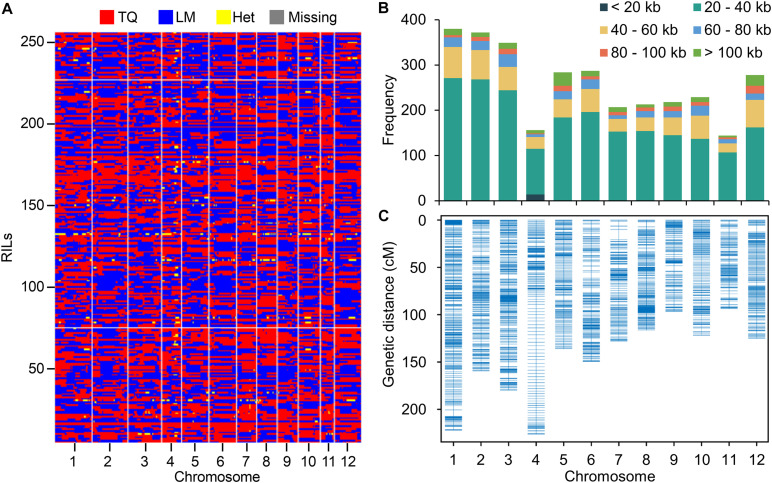
Resequencing RIL population and genetic linkage map construction. **(A)** Recombination bin map of 257 RILs. The red, blue, yellow and gray colors represent the genotypes of TQ and LM, the heterozygous genotype (Het), and the missing data. **(B)** The frequency of bins with different lengths on each chromosome. **(C)** Genetic linkage map of RIL population by using bins as markers.

When mapping the clean reads to the Nipponbare reference genome, a total of 1,472,076 SNPs between LM and TQ were identified with 86% of single nucleotide polymorphisms (SNPs) (1,262,315) being sequenced at 4 × or greater sequencing depth. The SNPs were used for constructing the bin map by a sliding window approach (Huang et al., 2009). In total, 3,117 bins were inferred from 257 RILs (Figure 1A). The physical length of bins ranged from 1.9 Kb to 779.9 Kb, with an average of 42.5 Kb and a median of 30.7 Kb (Supplementary Table 2). The length of 85% bins ranges from 20 Kb to 60 Kb (Figure 1B). To determine the heterozygosity of the RILs, the proportion of genomic regions that were heterozygous in the bin map was calculated. On average, only 0.29% of the genomic regions are heterozygous, establishing the high homozygosity of the LT-RIL population at the F_20_ generation (Figure 1A).

The bins with physical length > 20 kb (except for chromosome 4) were used as markers to construct the genetic map (Figure 1C). The total genetic distance of the genetic map was 1754.87 cM with the mean distance of the bins being 0.61 cM (Supplementary Table 3). The mean genetic distance of chromosome 4 (1.46 cM) is relatively high, possibly due to the relative small number of SNPs and bins identified on this chromosome (Supplementary Tables 2, 3). To assess the accuracy and quality of the bin map, bin markers were mapped to the Nipponbare reference genome. The scatter plot of bin markers in the 12 linkage groups aligned well with the reference chromosomes (Supplementary Figure 1), suggesting good collinearity between bin markers and the reference genome.

### Correlations of Elemental Concentrations in the Ionome

The LT-RIL population was grown in flooded field plots in 2002, 2003, 2006, and 2007, and under both flooded (anaerobic) and unflooded (aerobic) field conditions in 2008 (Supplementary Table 4) (Zhang et al., 2014). The concentrations of 16 elements in dehusked and unmilled rice grain were determined, including As, Ca, Cd, Co, Cu, Fe, K, Mg, Mn, Mo, Ni, P, Rb, S, Sr, and Zn. To minimize the variation in grain elemental concentrations caused by the variation in soil moisture (unflooded) or water depth (flooded), we grew the LT-RIL population in a greenhouse under a semi-flooded condition with an auto-irrigation system (11GGH: 20*11 g*rain *g*rown in *g*reenhouse). All elements exhibited a 2-fold or larger range in grain concentrations, and transgressive segregation was observed among the LT-RILs for all elements (Supplementary Figure 2). Significant differences of grain concentrations of all 16 elements were found in the LT-RIL population as revealed by an analysis of variance (ANOVA) (Supplementary Table 5). We further determined the concentrations of 16 elements in roots (15RTH: 20*15 r*oo*t* grown *h*ydroponically) and shoots (15SHH: 20*15 sh*oot grown *h*ydroponically) of 30-day-old RILs grown hydroponically. The concentrations of most elements in roots or shoots were significantly different in the LT-RIL population (Supplementary Table 5), and similar variation of elemental concentrations existed in the LT-RIL population (Supplementary Figures 3, 4). To determine the whether the frequency distribution of elemental concentrations is normal or skewed in the population, we calculated the skewness and kurtosis values and performed the Shapiro-Wilk test separately for each of 16 elements. The concentrations of most elements were distributed normally except the grain As, shoot As, shoot Ca, grain Fe, root K, shoot Mg, grain Sr and shoot Sr (Supplementary Table 6).

**FIGURE 2:**
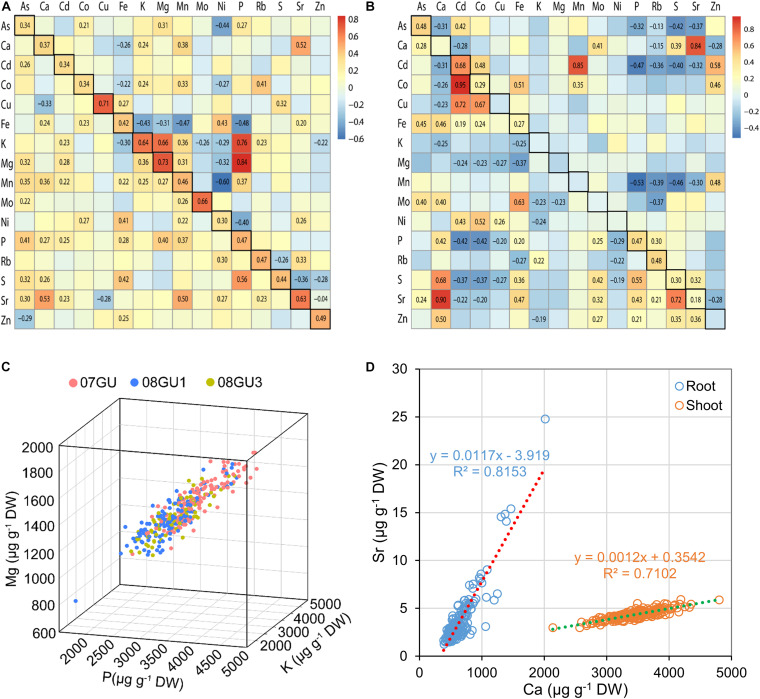
The correlations of elemental concentrations in the RIL population. **(A)** The correlations of elemental concentrations in grains of LT-RILs grown under unflooded condition (upper right triangular matrix) or in a greenhouse (lower left triangular matrix). The squares with black frames on the left diagonals are the correlations of the same elements in grains of RILs grown under flooded and unflooded condition. **(B)** The correlations of elemental concentrations in shoots (upper right triangular matrix) and roots (lower left triangular matrix) of RILs grown hydroponically. The squares with black frames on the left diagonals are the correlations of the same elements between shoots and roots. The Pearson’s correlation coefficients (*r*) were calculated between each pair of all 16 elements based on the mean of elemental concentrations in different tissues. Only the *r* values with significance levels at *p* < 0.01 were shown on the heat maps. **(C)** The correlation of concentrations of P, Mg, K in the grains of LT-RILs grown under unflooded condition. **(D)** The correlation of Ca and Sr concentrations in the shoots and roots of LT-RILs grown hydroponically.

Significant correlations between the concentrations of several elements were previously observed in the grains of LT-RILs grown under flooded conditions, including P-K-Mg, Ca-Sr, P-Fe, and Mn-Cd (Zhang et al., 2014). Similar correlations were also seen in grains of LT-RILs grown in unflooded field plots, particularly for P-K-Mg with Pearson’s correlation coefficients (*r*) of 0.76, 0.84, and 0.66 for P-K, P-Mg and K-Mg, respectively (Figure 2A). However, such positive correlation among P-K-Mg was much weaker in LT-RILs grown in a greenhouse under semi-flooded condition with *r*-values of 0.40 and 0.36 for P-Mg and K-Mg, respectively, and positive but not significant for P-K (Figure 2A). Growth condition specific differences in element correlations were also found for P-Fe, which were negatively correlated in grains from flooded and unflooded field plots (*r* = −0.39 and −0.48, respectively; *p* < 0.01) but were positively correlated when grown in semi-flooded pots (*r* = 0.28; *p* < 0.01) (Figure 2A; Zhang et al., 2014). In contrast, the chemical analogs, Ca-Sr, showed strong positive correlations in the grains of LT-RILs grown under all three different growth conditions (*r* = 0.66, 0.52 and 0.53 for flooded, unflooded and semi-flooded conditions, respectively; *p* < 0.01) (Figure 2A; Zhang et al., 2014). For each of the 16 elements determined, significant positive correlations were observed in the grains of LT-RILs grown in flooded and unflooded field plots with *r*-values ranging from 0.30 to 0.73 (*p* < 0.01) (Figure 2A).

We further calculated the correlation coefficients of all 16 elements in the shoots and roots of LT-RILs grown hydroponically. The positive P-Mg and K-Mg correlations observed in grains, whether produced in fields or pots, did not exist in either shoots or roots, suggesting tissue specific correlation of these three elements (Figures 2B,C). However, a stronger positive correlation between the chemical analogs Ca-Sr was found in both shoots and roots (*r* = 0.84 and 0.90 for shoots and roots, respectively; *p* < 0.01) (Figure 2D). Furthermore, significant correlations between Cd-Co (positive), Fe-Co (positive), Ca-S (positive), P-Cd (negative), S-Cd (negative) were observed in both shoots and roots; whereas, positive correlations between Cd-Mn, Cd-Zn and Zn-Mn were found in shoots but not in roots (Figure 2B). Among the 16 elements, only 8 showed significant correlations between shoots and roots, including As, Cd, Co, Fe, P, Rb, S, and Sr, all of which were positively correlated between shoots and roots (Figure 2B).

### Univariate QTL Mapping Based on the Concentrations of Individual Elements

By using 175 restriction fragment length polymorphism (RFLP) markers, Zhang et al. (2014) previously identified 40 ionomic QTLs affecting the concentration of 16 elements in unmilled rice grains of LT-RIL population grown under flooded condition (Zhang et al., 2014). The QTL analysis was performed based on the least squares (LS) means of each element across the five years’ replications (02GF: 20*02 g*rain grown in *f*looded condition; 03GF: 20*03 g*rain grown in *f*looded condition, 06GF: 20*06 g*rain grown in *f*looded condition, 07GF: 20*07 g*rain grown in *f*looded condition and 08GF2: 20*08 g*rain grown in *f*looded condition, field plot #*2*). In the present study, we took advantage of a high resolution bin map and performed QTL analyses for each year separately. QTL analyses were also performed on elemental concentrations measured in grains from LT-RIL plants grown under unflooded field conditions (07GU: 20*07 g*rain grown in *u*nflooded condition, 08GU1: 20*08 g*rain grown in *u*nflooded condition, field plot #*1*, and 08GU3: 20*08 g*rain grown in *u*nflooded condition, field plot #*3*), semi-flooded greenhouse conditions (11GGH: 20*11 g*rain grown in *g*reen*h*ouse), as well as in roots and shoots of LR-RILs grown hydroponically (Supplementary Table 4). The LT-RILs were planted at staggered times in order to synchronize the heading times among the various LT-RILs in 2002, 2003, and 2006 which synchronized also environmental factors such as temperature and sunlight during grain fill that could potentially affect the accumulation of elements in grains (Zhang et al., 2014). Accordingly, the LS means of grain elemental concentrations of 02GF, 03GF and 06GF (02-06_mean) were calculated and used as traits for QTL analysis. QTL analyses were also conducted on a composite flooded mean (F_mean) calculated as the LS mean across 02GF, 03GF, 06GF, 07GF, and 08GF2, plus an unflooded U_mean calculated the three unflooded replications 07GU, 08GU1, and 08GU3 (Supplementary Table 4).

**FIGURE 3:**
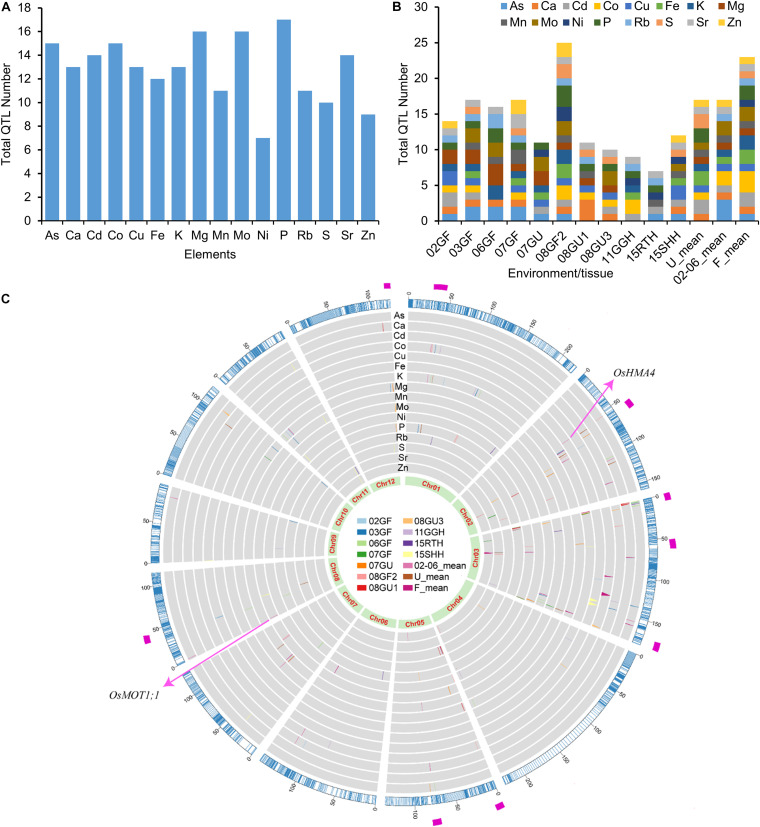
QTL numbers and distribution of elemental QTLs on the genetic linkage map. **(A)** Total QTL numbers identified for each element. **(B)** Total QTL numbers identified in different environments or tissues. **(C)** Distribution of elemental QTLs on the genetic linkage map. The outer ring in blue color is the genetic linkage map scaled with genetic distance (cM). The inner rings in gray background are the distribution of QTLs for the concentrations of 16 elements. QTLs identified from different years or tissues are presented in different colors as shown in the middle. The most inner ring shows the 12 rice chromosomes. The magenta rectangle in the most outer ring highlights the QTL hotpots. 02GF, 03GF, 06GF, 07GF and 07GU are grains (G) of RILs grown under flooded (F) or unflooded (U) conditions in 2002, 2003, 2006 and 2007, respectively; 08GF2, 08GU1 and 08GU3 are grains (G) of RILs grown under flooded (F) or unflooded (U) condition on 2008 in the field site number 2, 1 and 3; 11GGH is the grains (G) of RILs grown in a greenhouse (GH) condition on 2011; 15RTH and 15SHH are roots (RT) or shoots (SH) of RILs grown hydroponically (H) on 2015. 02-06_mean: the LS mean of 02GF, 03GF and 06GF; U_mean: the LS means of 07GU, 08GU1 and 08GU3; F_mean: the LS means of 02GF, 03GF, 06GF, 07GF and 08GF2.

Using the 3,117 bins as genetic markers, a total of 206 elemental QTLs were identified based on meeting or exceeding the logarithm of the odds (LOD) threshold values permuted for each trait (α = 0.05). The number of QTLs detected for each element ranged from 7 (Ni) to 17 (P) (Figure 3A). Among the 206 elemental QTLs, 25 were detected in at least two growth conditions or tissues, resulting in 167 unique QTLs (Supplementary Table 7). The LOD values of QTLs ranged from 3.01 to 24.33, and the QTLs explained up to 30.05% of the phenotypic variance (Supplementary Table 7). The number of QTLs detected varied by environment or tissues (Figure 3B). The smallest number of QTLs was found in the roots of LT-RILs grown hydroponically (15RTH, 7 QTLs) while the largest number was found in grains of LT-RILs grown under flooded condition in 2008 (08GF2; 25 QTLs) (Figure 3B). By aligning all detected QTLs in the genetic bin map, several QTL hotspot regions that contained at least 5 QTLs controlling the accumulation of the same or different elements were identified in the genetic map (Figure 3C). The existence of such QTL hotspots suggest the presence of genes with pleiotropic effects controlling the concentrations of multiple elements in these genomic regions.

### Tissue- and Environment-Specific Ionomic Patterns in the LT-RIL Population

**FIGURE 4:**
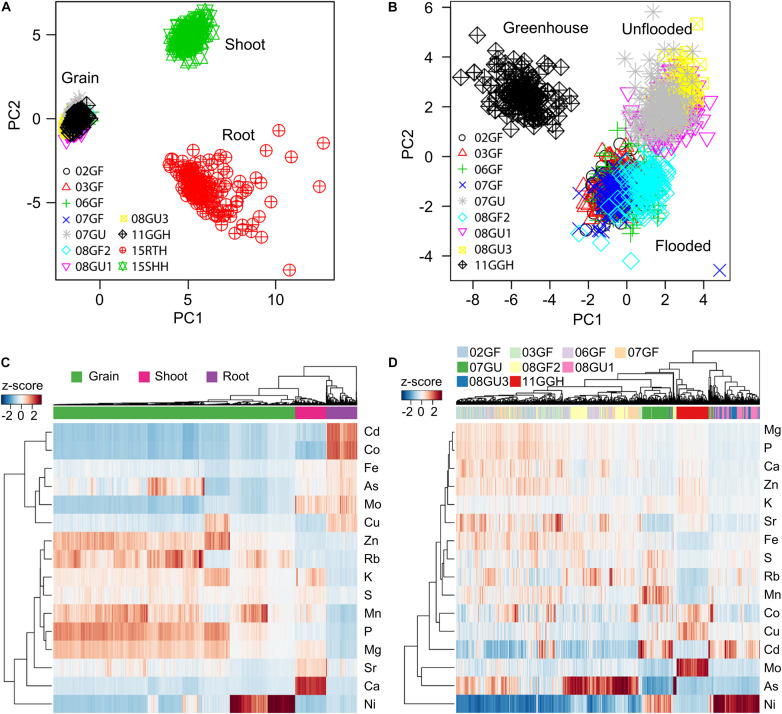
Tissue- and environment-specific ionomic pattern of RIL population. **(A,B)** Principle component analysis (PCA) of elemental concentrations in the grains, shoots and roots **(A)** and grains only **(B)** of RIL population grown in different conditions. PCA was performed based on the concentrations of 16 elements. **(C,D)** Hierarchical clustering of RILs based on the ionome of the grains, shoots and roots **(C)** and grains only **(D)**. The clustering was performed using the z-score values of each of RILs. 02GF, 03GF, 06GF, 07GF and 07GU are grains (G) of RILs grown under flooded (F) or unflooded (U) conditions on 2002, 2003, 2006, and 2007, respectively; 08GF2, 08GU1 and 08GU3 are grains (G) of RILs grown under flooded (F) or unflooded (U) condition on 2008 in the field site number 2, 1 and 3; 11GGH is the grains (G) of RILs grown in a greenhouse (GH) condition on 2011; 15RTH and 15SHH are roots (RT) or shoots (SH) of RILs grown hydroponically (H) on 2015.

To reveal the ionomic patterns of the different tissues obtained under different growth conditions, we performed PCA based on the concentrations of 16 elements in the roots, shoots and grains. A clear separation of principal component 1 (PC1) and PC2 among roots, shoots and grains was observed (Figure 4A), indicating different ionomic patterns for these three tissues. Although the grains were collected from RILs purposely grown under divergent conditions and in different years, the grain ionomic variations along both PC1 and PC2 were much smaller than those of roots and shoots. Further PCA analysis based on only the elemental concentrations in grains revealed a clearly distinct grain ionomic pattern in the RIL population when grown under flooded and unflooded field conditions and in a regularly irrigated and fertilized greenhouse potted-plant condition (Figure 4B).

A hierarchical clustering method was used to further determine the tissue- and environment-specific ionomic pattern of the LT-RIL population. As shown in Figure 4C, the roots, shoots and grains were exclusively clustered separately with each tissue locating in a unique cluster (Figure 4C), consistent with the tissue-specific ionomic pattern revealed by PCA (Figure 4A). Further hierarchical clustering based on grain concentrations of RILs grown under different conditions and in different years showed that LT-RILs grown in greenhouse (11GGH) and unflooded field conditions (07GU, 8U1, and 08GU3) were clustered separately, while RILs grown in flooded fields were generally grouped in a large cluster (Figure 4D). Taken together, PCA and hierarchical clustering revealed a clear tissue- and environment-specific ionomic pattern of the LT-RIL population.

### Principal Component Analysis of Covariance Among Elements in the Ionome Under Different Environments or in Different Tissues

**FIGURE 5:**
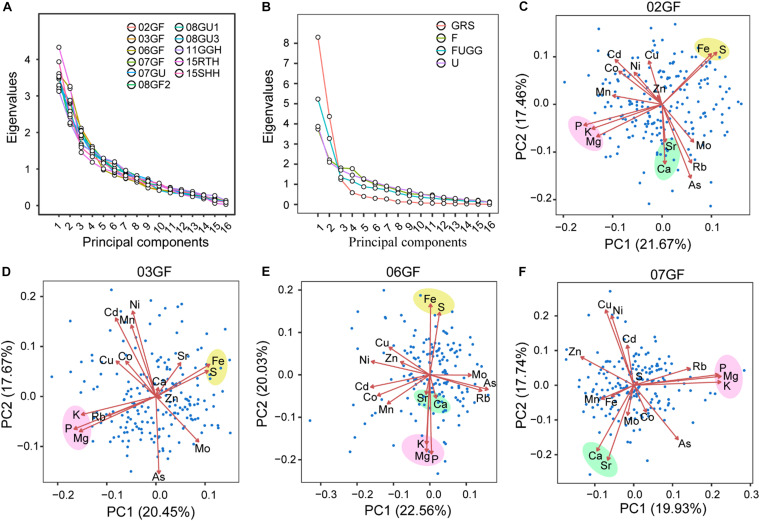
PCA plots in different environments or tissues. **(A,B)** Screeplots of 16 principle components of individual environment or tissue **(A)** or across environments or tissues **(B)**. **(C–F)** Biplots of 02GF **(C)**, 03GF **(D)**, 06GF **(E)** and 07GF **(F)**. The biplots showed the PC1 and PC2 loadings of each elements in indicated environments. The correlated elements with similar PC direction and magnitude were highlighted in color background. 02GF, 03GF, 06GF, 07GF and 07GU are grains (G) of RILs grown under flooded (F) or unflooded (U) conditions on 2002, 2003, 2006 and 2007, respectively; 08GF2, 08GU1 and 08GU3 are grains (G) of RILs grown under flooded (F) or unflooded (U) condition on 2008 in the field site number 2, 1 and 3; 11GGH is the grains (G) of RILs grown in a greenhouse (GH) condition on 2011; 15RTH and 15SHH are roots (RT) or shoots (SH) of RILs grown hydroponically (H) on 2015. F: 02GF+03GF+06GF+07GF+08GF2; U: 07GU+08GU1+08GU3; FUGG: F+U+11GGH; GRS: FUGG+15RTH+15SHH.

To better describe the correlation and covariance among elements, we performed principal component analysis (PCA) separately on the concentrations of 16 elements in grains of LT-RILs produced under different conditions or in shoots and roots of LT-RILs grown hydroponically. For each growth condition or tissue, 16 principal components were produced (Figure 5A). The PC1 explained the largest proportion of the variance of the ionome, ranging from 19.54% (11GGH) to 27.08% (15RTH) (Supplementary Table 8). Although the variance explained by PC1 differed among the growth conditions and tissues, PC1 and PC2 together could explain large proportion of the variance from 34% (11GGH) to 47% (15RTH) (Figure 5A; Supplementary Table 8). In total, the first four PCs explained more than 50% of the variance of the ionome for all growth conditions or tissues, and 90% of variance was accounted for by the first ten PCs (Supplementary Table 8).

The loadings of different elements varied in each PC (Supplementary Table 9). By plotting the loadings of PC1 and PC2 of all 16 elements, it was clear that the elements with the pair wise relationships observed in the element-by-element correlations tended to have similar loadings (Figures 5C,F; Supplementary Figures 5A–G). For example, the well correlated P-K-Mg observed in grains grown under flooded (Zhang et al., 2014), unflooded or semi-flooded conditions (Figure 2A), usually had very similar magnitude and direction of loadings under these conditions (Figures 5C–F; Supplementary Figures 5A–C). Similarly, the chemical analogs Ca and Sr that were strongly correlated in growth condition or tissues (Figures 3A,B) generally loaded PCs in a similar direction with a similar magnitude (Figures 5C,E,F; Supplementary Figures 5A–F). Some correlated element pairs showed similar loadings only in particular growth conditions. For example, the correlated Fe-S only had similar loadings in 02GF, 03GF and 06GF where the LT-RTLs were planted at different times to synchronize the heading time (Figures 5C–E). Such overlap in the loading of some elements in PC1 and PC2 in certain growth conditions suggested a consistent effect of genetic variation on the covariance of elemental accumulation in these conditions or tissues. While common patterns existed across growth condition or tissues for some elements, PC loadings of other elements differed in both magnitude and direction according to growth condition or tissues (Figures 5C–F; Supplementary Figures 5A–G), suggesting different covariance of elemental accumulation across the growth condition or tissues.

We further performed PCA on the elemental concentrations in grains of LT-RILs across different growth environments or across different tissues, including under all flooded conditions (02GF, 03GF, 06GF, 07GF, and 08GF2; F), all unflooded conditions (07GU, 8U1, and 08GU3; U), and combined flooded, unflooded and semi-flooded conditions in greenhouse (F, U and 11GGH; FUGG) as well as across all grains, roots and shoots (FUGG, 15RTH, and 15SHH; GRS). Compared to the single environment or tissue, PC1 generally explained a larger proportion of phenotypic variance across environments and tissues (Figure 5B; Supplementary Table 8). The PC1 explained as much as 51.87% of the variance of the ionome across the grains, shoots and roots (RGS), and PC1 and PC2 together explained approximately 80% of phenotypic variance (Figure 5B; Supplementary Table 8). The correlated element pairs that showed similar PC loadings among the individual growth environments were not observed across different environments. For example, the PC loadings of P-K-Mg were very similar in each flooded or unflooded growth conditions (Figures 5C–F; Supplementary Figures 5A–D); however, their PC loadings were only similar across unflooded conditions but not across flooded conditions (Supplementary Figures 5H–K). The PC loadings of the strongly correlated chemical analogs Ca and Sr which had often been similar among individual environments or tissues also differed in these analyses of combined environments and tissues (Supplementary Figures 5H–K). This suggests that the correlations of the element pairs are not stable within all plants and tissues, but are largely dependent on environment or tissue.

### Multivariate QTL Mapping on Ionomic Covariance Components Within or Across Environments/Tissues

The PC scores from each individual environment/tissue or across different environments/tissues were used as traits for QTL mapping. By using the PC scores of 16 PCs from individual environment/tissue (02GF, 03GF, 06GF, 07GF, 07GU, 08GF2, 08GU1, 08GU3, 11GGH, 15RTH, and 15SHH), 53 QTLs had LOD values above the permuted thresholds (α = 0.05) (Supplementary Table 10). These QTLs were termed as PC-QTLs. For each environment or tissue, at least two PC-QTLs were detected (Supplementary Figure 6A). The largest number of PC-QTL were detected in 06GF with 8 PC-QTLs identified. For each PC, at least one PC-QTLs were detected (Supplementary Figure 6B). PC9 had the largest number of PC-QTL (7 PC-QTLs) while only one PC-QTL was detected for PC12 and PC15 (Supplementary Figure 6B). The LOD values of PC-QTL ranged from 4 to 21, and explained from 5% to 35% of the trait variance. The PC-QTL of PC5 in 15RTH explained the highest phenotypic variance (35%) with LOD value of 20.65 (Supplementary Table 10). There were three genomic regions where at least two PC-QTLs co-localized, including two regions on chromosome 2 (08GU1_PC10 and 07GU_PC9; 03GF_PC5 and 06GF_PC7) and one on chromosome 3 (02GF_PC12, 07GF_PC16, 08GU1_PC15, and 07GU_PC14) (Supplementary Table 10).

**FIGURE 6:**
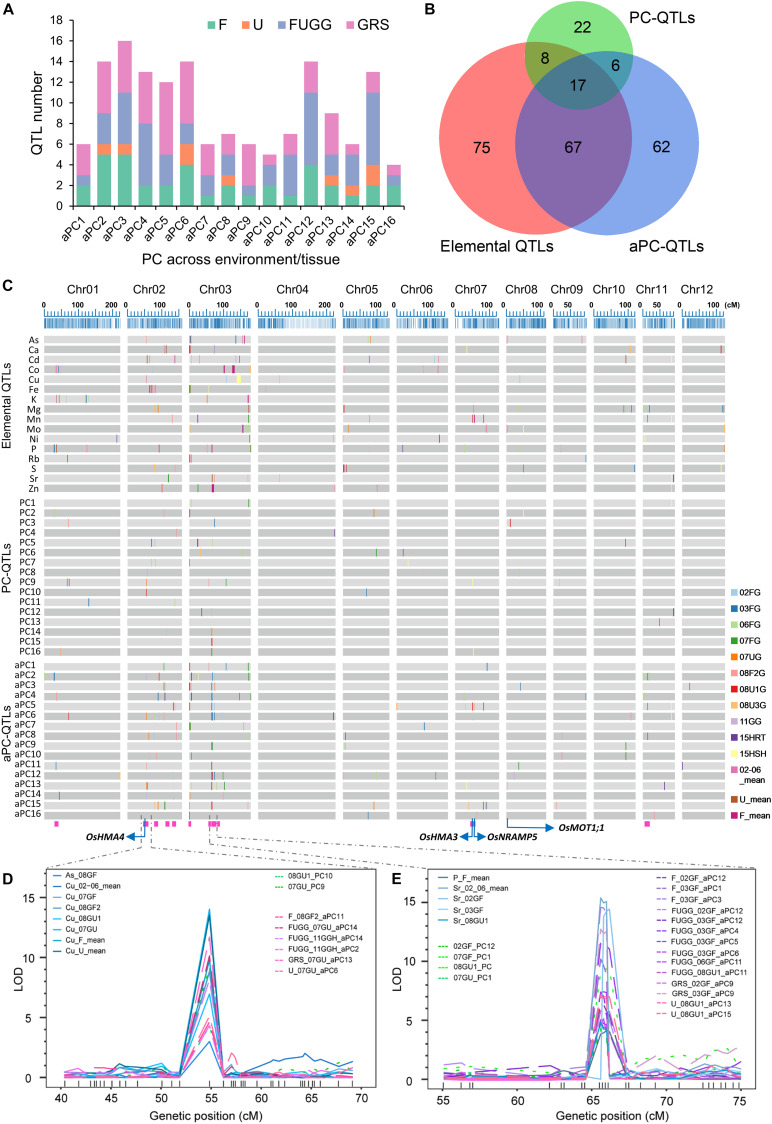
Identification of PC-QTLs and aPC-QTLs. **(A)** Total number of aPC-QTLs identified across different environments or tissues. **(B)** The overlapped number of elemental QTLs, PC-QTLs and aPC-QTLs as revealed by a Venn diagram. **(C)** Distribution of elemental QTLs, PC-QTLs and aPC-QTLs on the 12 linkage groups of the genetic map. Elemental QTLs were separated by elements; PC-QTPs and aPC-QTLs were separated by PCs. QTLs detected in different environments or tissues are shown in different colors. The magenta rectangles at the bottom highlight the positions of the QTL clusters. The blue arrows show the positions of four candidate genes: OsHMA4, OsHMA3, OsNRAMP5 and OsMOT1;1. **(D,E)** The LOD values of super QTLs with co-localization of elemental QTLs, PC-QTLs and aPC-QTLs on chromosome 2 **(D)** and chromosome 3 **(E)**. Elemental QTLs, PC-QTLs and aPC-QTLs are shown as solid lines, dot lines and break lines, respectively. Genetic markers are shown in the bottom of each figures. 02GF, 03GF, 06GF, 07GF and 07GU are grains (G) of RILs grown under flooded (F) or unflooded (U) conditions on 2002, 2003, 2006, and 2007, respectively; 08GF2, 08GU1 and 08GU3 are grains (G) of RILs grown under flooded (F) or unflooded (U) condition on 2008 in the field site number 2, 1 and 3; 11GGH is the grains (G) of RILs grown in a greenhouse (GH) condition on 2011; 15RTH and 15SHH are roots (RT) or shoots (SH) of RILs grown hydroponically (H) on 2015. F: 02GF+03GF+06GF+07GF+08GF2; U: 07GU+08GU1+08GU3; FUGG: F+U+11GGH; GRS: FUGG+15RTH+15SHH.

We further used the PC scores of 16 PCs across environments/tissues as traits for QTL analyses, including the PC scores across all flooded conditions (02GF, 03GF, 06GF, 07GF, and 08GF2; F), unflooded conditions (07GU, 8U1, and 08GU3; U), and combined flooded, unflooded and semi-flooded conditions in greenhouse (F, U and 11GGH; FUGG) and in both grains, roots and shoots (FUGG, 15RTH and 15SHH; GRS). Altogether, 152 QTLs were identified which were termed as across environments/tissues PC-QTLs (aPC-QTLs) (Supplementary Table 11). The largest number of aPC-QTLs were detected across grains, shoots and roots (GRS, 54 aPC-QTLs) while only 9 aPC-QTLs were identified across the unflooded condition (U) (Supplementary Figure 6C). For each across environments/tissues PC (aPC), at least four aPC-QTLs were detected, and aPC3 had the largest number of aPC-QTL (16 aPC-QTLs) (Figure 6A). The highest LOD value of aPC-QTL was 17.48, and explained up to 26.14% of the phenotypic variance (Supplementary Table 11). Twenty-one aPC-QTL clusters occurred among the 152 aPC-QTLs detected with an average of 3.76 aPC-QTLs in each cluster (Supplementary Table 11). A super aPC-QTL cluster was found on chromosome 3 in which 14 aPC-QTLs co-localized (Figure 6C and Supplementary Table 11). The identification of PC-QTLs and aPC-QTLs in the LT-RIL population was helpful in dissection of covariation of multiple elements in the ionome.

### Co-localization of Elemental QTLs With PC-QTLs and aPC-QTLs

To determine whether QTL mapping using PCs or aPCs as traits identifies novel QTLs that were not detected based on the concentration of single element, we compared the genetic locations of elemental QTLs with PC-QTLs or aPC-QTLs. By aligning the QTLs on the genetic map, we found that a substantial number of PC-QTL or aPC-QTL did not co-localize with elemental QTLs (Figures 6B,C). Among the 53 PC-QTLs, 25 of them (47%) co-localized with at least one elemental QTL, and 28 PC-QTLs were not detected based on the elemental concentrations (Figure 6B). Furthermore, the QTL mapping interval of 68 of 152 aPC-QTLs did not overlap with any elemental QTLs, accounting for 45% of the total aPC-QTLs detected. Interestingly, among the 28 novel PC-QTLs and 68 novel aPC-QTLs, 6 of them had identical QTL intervals (Figure 6B). In all, 90 novel QTLs were identified based on the PC scores either within or across environments/tissues.

We further analyzed the overlapping QTLs among elemental QTLs, PC-QTLs and aPC-QTLs. Among the 167 unique elemental QTLs, 25 QTLs (15%) were co-localized with at least one PC-QTL, 84 QTLs (50%) overlapped with at least one aPC-QTL, and 17 QTLs overlapped with both PC-QTLs and aPC-QTLs (Figure 6B and Supplementary Table 12). A total of 92 elemental QTLs co-localized with at least one PC-QTL or aPC-QTL, accounting for 55% of the elemental QTLs detected. We extracted the elements with the top 5 PC loadings for each PC-QTL or aPC-QTL that were co-localized elemental QTLs. For most of the PC-QTL (27/31) and aPC-QTLs (74/101), the elements with the top 5 PC loadings generally included the corresponding element for the co-localized elemental QTLs (Supplementary Table 12). These results suggested that these QTLs could be detected based on either elemental concentrations or PC scores.

### Identification of QTL Clusters and the Candidate Genes

Among the co-localized QTLs, we focused on those loci identified by all three types of QTLs, including elemental QTLs, PC-QTLs and aPC-QTLs. we called these loci QTL clusters. There were a total of 15 such QTL clusters across the genome (Figure 6C and Table 1). For 14 of 15 QTL clusters, the elements corresponding to elemental QTLs were included in the elements with the top 5 PC loadings for the PC-QTLs and/or aPC-QTL. A super QTL cluster (Cluster 2) was found on chromosome 2 from 51.838-56.239 cM (Figure 6D and Table 1). In this region, there was one grain As QTL and seven grain Cu QTLs, two PC-QTLs and 6 aPC-QTLs. The LOD values of these QTLs ranged from 3.01 to 14.40, and explained up to 26.35% of phenotypic variation. The elements with top 5 PC loadings for all PC-QTLs and aPC-QTLs included Cu and/or As. By searching for the putative candidate genes in this genomic region, a gene encoding a heavy metal P-type ATPase OsHMA4 was identified, which had been previously shown to function to sequester Cu into root vacuoles and limited Cu accumulation in rice grain (Huang et al., 2016), suggesting *OsHMA4* may be responsible for this QTL cluster. The second super QTL cluster (Cluster 8) was identified on chromosome 3 which contained four grain Sr QTLs, one grain P QTL, seven PC-QTLs and nine aPC-QTLs (Figure 6E and Table 1). Except for aPC-QTL of FUGG_03GF_aPC6, all other PC-QTLs and aPC-QTLs top PC scores elements contained Sr. In this region, two genes encoding heavy metal-associated domain containing proteins were likely responsible for this QTL cluster (Table 1).

**TABLE 1:** QTL clusters with elemental QTL, PC-QTL and aPC-QTL co-localized.

QTL cluster	Chr^1^	Genetic Position (cM)	Physical position (Kb)	Elemental QTL		PC-QTL		aPC-QTL		Candidate gene	
				Element	Environment/tissue	PC	Top 5 elements^2^	aPC^3^	Top 5 elements^2^	ID	Annotation
1	1	28.704 ∼ 29.094	4151 ∼ 4469	P	03GF_Grain	06GF_PC2	**P** Mg Fe K S	F_03GF_aPC2	Mg Cu **P** As Cd	LOC_Os01g08660	Aquaporin protein (SIP1;1)
2	2	51.838 ∼ 54.839	4987 ∼ 5798	Cu	07GF_Grain	07GU_PC9	**Cu** Ni Fe	F_08GF2_aPC11	Cd **As** Mn Zn Rb	LOC_Os02g10290	Heavy metal-transporting P1B type ATPase (OsHMA4)
				Cu	08GF2_Grain	08GU1_PC10	**As** Cd	U_07GU_aPC6	**Cu** Fe Mo **As** Co		
				Cu	08GU1_Grain		**As** Fe S Ni	FUGG_07GU_aPC14	**Cu** Mo K Mg **As**		
				Cu	07GU_Grain		Zn	FUGG_11GGH_aPC14	**Cu** Mo K Mg **As**		
				Cu	F_mean_Grain			FUGG_11GGH_aPC2	**As Cu** K Ni Cd		
				Cu	U_mean_Grain			GRS_07GU_aPC13	**Cu** Co Cd **As**		
				Cu	02-06_mean_Grain						
				As	08GF2_Grain						
3	2	79.626 ∼ 80.016	15571 ∼ 16509	Mg	08GU3_Grain	02GF_PC5	Mo Zn Mn Fe Rb	FUGG_08GU3_aPC15	Ni Mo P K Mn	LOC_Os02g26700	Cation transport regulator-like protein (OsARP)
4	2	106.137 ∼ 106.529	21244 ∼ 21828	Ca	06GF_Grain	08GF2_PC2	Sr Ca Mn P K	F_07GF_aPC1	Ni S As P K	LOC_Os02g36414	Transporter family protein Transporter family protein Transporter family protein
								FUGG_07GF_aPC3	Ca Mn Sr Cd As	LOC_Os02g36440	
								GRS_08GF2_aPC6	Mn K P Zn Sr	LOC_Os02g36450	
5	2	138.302 ∼ 138.497	28585 ∼ 29657	Cd	02GF_Grain	06GF_PC11	Mn Rb Ni **Cd** Zn	F_08GF2_aPC5	Co Rb Fe Cu S	LOC_Os02g46990	AAA-type ATPase family protein
				Cd	F_mean_Grain						
6	3	0.783 ∼ 1.175	731 ∼ 1013	As	02GF_Grain	08GU1_PC2	Ca Mn Sr	GRS_08GU1_aPC1	Mo Fe S **As** Sr	LOC_Os03g02380	Major facilitator superfamily domain-containing protein (OsCd1)
				Rb	02GF_Grain		P **Rb**	GRS_08GU1_aPC3	Ni **Rb** P Mn		
				Rb	08GU1_Grain			GRS_02GF_aPC4	**Rb** Ni Zn		
								GRS_08GU1_aPC5	Mn Sr Zn Cd Co		
								GRS_08GU1_aPC7	Zn Sr S **Rb** K		
7	3	56.866 ∼ 57.061	13858 ∼ 14577	Fe	15SHH_Shoot	08GF2_PC9	Mn Rb **Fe** Ni Ca	FUGG_08GF2_aPC1	Mo P Mg Zn **Fe**	LOC_Os03g24860	Transporter family protein Transporter family protein
										LOC_Os03g24870	
8	3	65.599 ∼ 66.185	15009 ∼ 15680	Sr	02GF_Grain	02GF_PC12	**Sr** Ca K Zn	F_02GF_aPC12	**Sr** S Ca As Zn	LOC_Os03g26650	Heavy metal-associated domain containing protein Heavy metal-associated domain containing protein
				Sr	03GF_Grain	07GF_PC16	Co	F_03GF_aPC12	**Sr** S Ca As Zn	LOC_Os03g27040	
				Sr	08GU1_Grain	08GU1_PC15	Ca **Sr** As	F_03GF_aPC3	**Sr** Ca Cu **P** Mg		
				Sr	02-06_mean_Grain		Mg Mo	U_08GU1_aPC13	K Mg **Sr** Ni S		
				P	F_mean_Grain	07GU_PC14	Ca **Sr** Mn	U_08GU1_aPC15	Ca Mn **Sr** Ni Fe		
							Mg As **Sr**	FUGG_02GF_aPC12	S **Sr** Ni K Rb		
							K S	FUGG_03GF_aPC12	S **Sr** Ni K Rb		
								FUGG_03GF_aPC4	Mg Rb Co Zn **P**		
								FUGG_03GF_aPC5	Rb **Sr** As K Co		
								FUGG_03GF_aPC6	Co Zn S K Rb		
								FUGG_06GF_aPC11	Ca **Sr** Zn S As		
								FUGG_08GU1_aPC11	Ca **Sr** Zn S As		
								GRS_02GF_aPC9	**Sr** Mo Ka S Cu		
								GRS_03GF_aPC9	**Sr** Mo Ka S Cu		
9	3	67.38 ∼ 67.575	15630 ∼ 16243	Sr	06GF_Grain	06GF_PC5	Rb Cu Mo	F_03GF_aPC6	Mo Fe Mn Zn Co	LOC_Os03g27960	Sodium calcium exchanger protein Potassium channel protein
				Sr	07GF_Grain		Zn Mn	FUGG_02GF_aPC4	Mg Rb Co Zn P	LOC_Os03g28120	
				Sr	F_mean_Grain			FUGG_03GF_aPC2	As Cu K Ni Cd		
				Sr	U_mean_Grain			FUGG_08GU1_aPC12	S **Sr** Ni K Rb		
								GRS_07GF_aPC9	**Sr** Mo K As Cu		
10	3	72.964 ∼ 73.276	16895 ∼ 17416	Ca	03GF_Grain	03GF_PC3	**Ca** Sr S Mn	FUGG_02GF_aPC2	As Cu K Ni Cd	LOC_Os03g29850	Metal cation transporter
				Ca	F_mean_Grain		Cu	FUGG_03GF_aPC3	**Ca** Mn Sr Cd As		
								GRS_03GF_aPC6	Mn K P Zn Sr		
								GRS_03GF_aPC12	S Mo As		
11	3	73.785 ∼ 74.097	17130 ∼ 17534	Ca	02GF_Grain	02GF_PC3	Sr **Ca** Mn Ni P	FUGG_02GF_aPC3	**Ca** Mn Sr Cd As	LOC_Os03g29920	Heavy metal transport/detoxification protein domain containing protein
12	3	173.329 ∼ 173.915	34655 ∼ 35388	Mg Mg	08GF2_Grain	07GF_PC1	**Mg** K P Rb	F_07GF_aPC2	**Mg** Cu P As Cd	LOC_Os03g61290	Cation/proton antiporter MATE efflux family protein
					U_mean_Grain		Zn	FUGG_06GF_aPC8	Fe **Mg** Cd P Mn	LOC_Os03g62270	
13	7	51.187 ∼ 51.382	7007 ∼ 7904	Mn	08GU1_Grain	15SHH_PC9	Rb K Ni As Zn	GRS_08GU1_aPC5	**Mn** Sr Zn Cd Co	LOC_Os07g12900	Heavy metal-transporting P1B type ATPase (OsHMA3)
14	7	52.367 ∼ 52.756	8331 ∼ 9044	Mn	06GF_Grain	15SHH_PC16	**Mn** Cd Sr	F_06GF_aPC6	Mo Fe **Mn** Zn Co	LOC_Os07g15370	Natural resistance associated macrophage protein 5 (OsNRAMP5)
							Ca As	GRS_06GF_aPC6	**Mn** K P Zn Sr		
15	11	83.595 ∼ 83.79	19351 ∼ 19879	Sr Zn	15SHH_Shoot	15SHH_PC1	Cd Mn **Zn**	GRS_15SHH_aPC5	Mn **Sr Zn** Cd Co	LOC_Os11g34350	ATP-binding cassette sub-family E member (OsABCE2)
					15SHH_Shoot		S As	GRS_15SHH_aPC6	Mn K P **Zn Sr**		

*^1^: Chr, chromosome.*

*^2^: The elements with top 5 PC scores for corresponding PC or aPC were listed in order from highest loading to lowest loading. Only elements with the loading > 0.2 or < −0.2 are shown. The elements in bond were those corresponding to the elements controlled by elemental QTLs.*

*^3^: 02GF, 03GF, 06GF, 07GF and 07GU are grains (G) of RILs grown under flooded (F) or unflooded (U) conditions on 2002, 2003, 2006 and 2007, respectively; 08GF2, 08GU1 and 08GU3 are grains (G) of RILs grown under flooded (F) or unflooded (U) condition on 2008 in the field site number 2, 1 and 3; 11GGH is the grains (G) of RILs grown in a greenhouse (GH) condition on 2011; 15RTH and 15SHH are roots (RT) or shoots (SH) of RILs grown hydroponically (H) on 2015. F: 02GF+03GF+06GF+07GF+08GF2; U: 07GU+08GU1+08GU3; FUGG: F+U+11GGH; GRS: FUGG+15RTH+15SHH.*

We further identified the candidate genes for other QTL clusters based on the annotation of genes in the QTL mapping intervals. QTL cluster 14 on chromosome 7 contained a grain Mn QTL, one PC-QTL and two aPC-QTL which all contained Mn in the top 5 elements with highest PC loadings (Table 1). In the QTL mapping interval of this QTL cluster, OsNRAMP5 (Natural Resistance Associated Macrophage Protein 5) which mediates the uptake of Cd and Mn in rice was identified (Ishikawa et al., 2012; Ishimaru et al., 2012; Sasaki et al., 2012; Yang et al., 2014), suggesting *OsNRAMP5* was likely the causal gene for this QTL cluster. Another QTL cluster (Cluster 13) related to Mn was also found on chromosome 7 (Table 1). A heavy metal P-type ATPase OsHMA3 which is responsible for sequestering Cd into the vacuoles in rice (Ueno et al., 2010; Miyadate et al., 2011), was found in this region. On the top of chromosome 3, Cluster 6 contained two grain Rb QTLs, one grain As QTL, one PC-QTL and five aPC-QTLs. A major facilitator superfamily domain-containing protein (OsCd1) which is involved in root Cd uptake and contributes to grain accumulation in rice was identified as the candidate gene for this QTL cluster (Yan et al., 2019). At least one candidate gene was isolated for other QTL clusters; however, their functions are largely unknown.

By mapping the sequencing reads of TQ and LM to the Nipponbare reference genome, we identified the SNPs and indels between TQ and LM. For most of the candidate genes of the QTL clusters, at least one nonsynonymous SNP or indel was identified in the coding sequences of the candidate genes (Supplementary Table 13). For example, five nonsynonymous SNPs was identified for *OsHMA3*, and one nonsynonymous SNP for *OsHMA4* which has previously been shown to change the Cu transporting activity of OsHMA4 (Huang et al., 2016). The indels were also found in the promoter regions of candidate genes (Supplementary Table 13), which may change their expression levels. However, further studies are required to confirm the causality of these candidate genes for corresponding QTLs.

## Discussion

In this study, we resequenced the genomes of 257 LT-RILs as well as the parental lines and constructed a bin map using SNPs polymorphic between the two parents, creating opportunity for high resolution QTL mapping in the LT-RIL population. Using the newly high density linkage map, we performed univariate and multivariate QTL analyses separately on the concentrations of 16 elements in three different tissues (grains, shoots and roots) of LT-RILs grown in seven different years under four growth conditions (flooded, unflooded, semi-flooded in greenhouse, and hydroponic). For the univariate QTL analyses, concentrations of individual elements were used as traits and QTL mapping was performed separately for each environment or tissue. Altogether, 206 QTLs were identified, and 25 of which were repeatedly detected in different environments or tissues, and resulted in 167 unique QTLs (Figures 3A,B; Supplementary Table 7). Compared to the 40 QTLs identified in our previous QTL mapping based on the least squares means of the concentrations of individual elements across five years’ replications (02GF, 03GF, 06GF, 07GF, and 08GF2) (Zhang et al., 2014), more QTLs (89 QTLs for 02GF, 03GF, 06GF, 07GF, and 08GF2; Supplementary Table 7) were detected by performing QTL analysis separately for each year, but potentially including environment-specific QTLs, with small research or breeding value. The allelic effects of parental lines in controlling the elemental concentrations varied among the elemental QTLs. For the elemental QTLs repeated detected in different growth conditions, the TQ allele was generally associated with increased elemental concentrations, such as the grain K QTL on chromosome 1 (35.433 ∼ 35.628 cM), the grain S QTL on chromosome 5 (1.019 ∼ 1.331 cM) as well as the grain Sr QTL on chromosome 3 (67.38∼ 67.575 cM) (Supplementary Table 7). Two QTLs that were cloned based on their discovery in the LT-RIL population were detected again in our current QTL analyses, and now mapped with much smaller mapping intervals. The QTL *qGCu2-1* on chromosome 2 that controls the accumulation of Cu in rice grain was repeatedly detected in four environments (Supplementary Table 7; Huang et al., 2016). Similarly, the grain Mo QTL *qGMo8* with a molybdate transporter gene *OsMOT1;1* as causal gene was detected on the top of chromosome 8 in the current analysis (Figure 3C; Supplementary Table 7; Huang et al., 2019). Furthermore, the QTL mapping precision was much increased by using the high density bin map compared to the use of 176 RFLP markers in previous studies (Zhang et al., 2014). For example, the QTL *qGCu2-1* mapped to an interval of 31.5 cM using RFLP markers but was mapped more precisely to a 4.4 cM interval using 3,117 bins as markers (Supplementary Table 7). The relatively small mapping interval of elemental QTLs identified in this study reduces need for fine-mapping and reduces the number of candidate genes that must be considered to identify and eventually confirm the causal gene through cloning or transgene validation.

The ionome is defined as the composition of the mineral nutrients and trace elements in an organism (Lahner et al., 2003; Salt et al., 2008). One of the typical characteristics of the ionome is its environment and tissue specificity. By using the PCA and hierarchical clustering methods, clear tissue-specific ionomic patterns were revealed among the grains, shoots and roots of LT-RILs (Figures 4A,C). This may be due to the different concentrations of certain elements in different tissues. For example, the concentrations of Cd and Co in roots and Ca and Sr in shoots were much higher than that in grains (Figure 4C). Even in the same tissue, the grains for example, the ionome also varies according to the growth environment. The levels of grain Cd of LT-RILs grown under unflooded condition are generally higher than that under flooded condition; whereas, the level of As shows an opposite trend (Figure 4C). The reasons for these differences can be explained largely by the distinct bioavailability of Cd and As in soils where Cd is less phytoavailable under anaerobic flooded conditions but As is more available [see review Zhao et al., 2015]. Such tissue- and environment-specific ionomic patterns have also been observed in several other studies (Yang et al., 2018; Fikas et al., 2019; Wang et al., 2020).

The elements in the ionome tend to not behave independently but covary depending on the tissue or environment. The covariation of multiple elements in the ionome could be explained in several ways. Firstly, multiple elements could be transported by the same transporter. Although most transporters have specific transport substrates, some transporters appear to transport two or more elements. For example, the iron transporter IRT1 also can transport Mn, Zn, and Cd (Korshunova et al., 1999). Elements with similar chemical properties often share the same transporters, such as S and Se, which are taken up by plants in the forms of sulphate and selenite, respectively, and enter roots via sulfate transporters (Sors et al., 2005). Strong correlation between Ca and Sr was true for grains, shoots and roots (Figures 2A,B), which indicated these two elements share the same transporting system due to their similar chemical properties. Therefore, the alteration of the transporting activity of transporters with non-specific substrates may change the accumulation of multiple elements. Secondly, element concentrations in plant tissues covary in response to environmental factors, particularly the nutritional status in soils. For example, the concentrations of mineral nutrients in the straw or grain of rice plants are generally decreased when grown in low nitrogen or low phosphorus conditions, including Na, Fe, As, Cd, and Pb (Yang et al., 2018). The concentrations of several elements vary under flooded and unflooded watering regimes such as As, Cd, Ni and Cu (Pinson et al., 2015). Such environment dependent covariation of element accumulation may explain the correlation of Fe-S only being observed in 02GF, 03GF and 06GF where LT-RILs were planted to synchronize heading and grain fill (Figures 5C–F). Finally, alterations of particular plant structures essential for blocking or transporting mineral nutrients may have a common effect on the accumulation of multiple elements. The Casparian strip-bearing endodermis in roots is one of such special structure, which acts as a bidirectional barrier to both the free diffusion of mineral nutrients from the soil into the vasculature and prevention of the backflow of nutrients from the stele to the soil (Geldner, 2013; Doblas et al., 2017). Several *A. thaliana* mutants with endodermal barrier defects were found to have higher levels of potassium (K) and sulfur (S) but lower levels of Ca, Mn and Fe in shoots (Hosmani et al., 2013; Kamiya et al., 2015; Li et al., 2017).

To better understand the genetic basis of covariation of multiple elements, we used PCA as a multivariate analysis method and performed QTL analysis using the PC scores as traits. PCA can transform the correlated elements into uncorrelated variables as principal components, which reduces the dimensionality of the ionome while retaining most of the variation in the original data set. The eigenvectors of the eigenvalues of the elemental covariance matrix, or the PCs, could be considered as new phenotypes for QTL mapping (Topp et al., 2013; Fikas et al., 2019). Although the PC scores seem to have little biological meaning, QTL mapping by using PC scores as traits could detect genetic loci that control the variation of complex phenotypes (Topp et al., 2013; Fikas et al., 2019). By using the PC scores from the PCA on elemental concentrations of LT-RILs in individual environment/tissue or across environments/tissues as traits, numerous PC-QTLs and aPC-QTLs were detected with their QTL mapping intervals were not overlapped with elemental QTLs (Figure 6B, Supplementary Table 12). These results suggested that multivariate QTL mapping using PC scores as traits could identify novel PC-specific QTLs that were not detected based on elemental concentration in the univariate QTL mapping. Such PC-specific QTLs may represent the genetic loci controlling the covariation of multiple elements which were not detected by QTL analysis based on single elements.

A considerable proportion of PC-QTLs or aPC-QTLs were co-localized with elemental QTLs. A total of 25 PC-QTLs and 84 aPC-QTLs were found to be co-localized with at least one elemental QTLs, which mean 55% of elemental QTLs (92/167) did not overlapped with PC QTLs (Figure 6B). Therefore, both univariate or multivariate QTL mapping could have identified common genetic loci. The PC loadings could be used to identify phenotypic variables with a substantial association with each selected PC. For 87% of PC-QTLs and 73% of aPC-QTLs, the elements with top 5 PC loadings generally included the corresponding elements for the co-localized elemental QTLs (Supplementary Table 12), suggesting the significant contribution of these elements for corresponding PCs or aPCs.

The literature contains one report where multivariate analysis based on PC scores not only identified the genetic loci controlling the covariation of multiple correlated traits but also aided in cloning the underlying causal genes. A recent GWA mapping based on the PC scores derived from 8 correlated architecture traits has identified the *OsSPY* gene in control of plant architecture in rice (Yano et al., 2019). We have identified the candidate genes for the QTL clusters with both elemental QTLs, PC-QTLs and aPC-QTLs co-localized (Table 1). For the QTL cluster 2 with two PC-QTLs and 6 aPC-QTLs co-localized on chromosome 2, the PC loading of Cu generally ranked in the top 2 elements with highest PC loadings (Figure 6D and Table 1). Therefore, a heavy metal P-type ATPase gene *OsHMA4* in the QTL mapping interval, which controls Cu accumulation in rice grain (Huang et al., 2016), was a strong candidate gene for this QTL cluster (Figure 6D and Table 1). The second obvious candidate gene is the Cd and Mn transporter gene *OsNRAMP5* (Ishikawa et al., 2012; Ishimaru et al., 2012; Sasaki et al., 2012; Yang et al., 2014) for the QTL cluster 14 which Mn was the strong associated element for the PC-QTL and aPC-QTL at this locus (Table 1). Although candidate genes were identified for other QTL clusters, further studies are required to confirm their causalities and underlying mechanisms. By developing the molecular markers, the elemental or PC QTLs identified in this study could be used in breeding rice varieties with enriched essential mineral nutrients and reduced toxic elements in the grains based on molecular marker-assisted selection.

In summary, multivariate QTL analysis based on the PC scores derived from PCA within or across environments/tissues enabled the identification of PC-specific QTLs that were not detected by traditional univariate QTL mapping based on the concentration of individual element. PCA based multivariate QTL analysis was also able to detect the genetic loci captured by QTL mapping with individual elements. Therefore, use of multivariate as well as univariate QTL analyses combined will provide additional knowledge with which to better understand the covariation of elements in the ionome and uncover more of the genes that regulate the accumulation of elements in plants.

## Materials and Methods

### Plant Materials and Growth Conditions

The rice (*O. sativa* L.) recombinant inbred lines (LT-RILs) were derived from a cross between the United States tropical *japonica* rice cultivar ‘Lemont’ (LM) and an *indica* cultivar from China, ‘TeQing’ (TQ) as described previously (Tabien et al., 2000; Zhang et al., 2014). The plants grown in replicated field plots in 2002, 2003, 2006, 2007, and 2008 were in the generations of F_15_, F_16_, F_17_, F_18_, and F_19_, respectively. The potted plants grown to maturity in a greenhouse in 2011, and seedlings grown hydroponically in 2015 were in the F_20_ and F_21_ generations, respectively. Seeds of the LT-RIL population (generation F21) is available in the USDA-ARS Genetic Stocks-Oryza (GSOR) Collection located at the USDA-ARS, Dale Bumpers National Rice Research Center, Stuttgart, AR, United States^1^.

Grains for ionome analysis were harvested from LT-RILs grown in drill-seeded field plots in Beaumont, TX, United States in 2002, 2003, 2006, 2007, and 2008, with details of the field growth conditions and replications per year described in detail in our previous grain ionome study (Zhang et al., 2014). Briefly, LT-RILs were grown under flooded condition, one replication per year using repeated parental plots for an Augmented field design in 2002 (02GF), 2003 (03GF), and 2006 (06GF). In 2007 and 2008, LT-RILs were grown in four separate nearby paddy fields with two fields originally designed to grow one replication each of LT-RILs under flooded condition and two fields to grow one replication each under unflooded condition. However, due to lodging of plants from storm damage, in 2007 we obtained seed free from soil contamination from one flooded (07GF) plus one unflooded replication (07GU), and in 2008 undamaged seed was harvested from one flooded (08GF2) and both unflooded replications (08GU1, 08GU3). Each year fields were fertilized prior to planting with 33.6 Kg/ha P and 73 Kg/ha N^+^ (as urea). In the flooded fields, approximately 8–16 cm depth flood water was applied to the fields at 4 to 5 weeks after planting, while plants were in the active tillering stage, and a minimum of 8 cm flood depth was maintained on the fields continuously until after grains were harvested. To prevent water stress and maintain the health of plants grown under unflooded field condition, fields were flush irrigated as needed with water which was held on the field for only 5–14 h per flush to prevent the alterations in soil chemistry and nutrient availability known to occur in anaerobic, flooded soils. The first flush irrigation was synchronized with the application of continuous flooded to the flooded fields, and flush irrigations were applied as needed to the unflooded fields to prevent drought stress throughout the growing season.

In 2011 (11GGH), the LT-RIL population was grown in a greenhouse at Purdue University as described previously (Huang et al., 2016). Briefly, plants were grown in 10 × 10 × 12 cm plastic pots filled with sandy soil, and the pots were placed in 4 cm deep trays that were automatically irrigated every day with tap water to full capacity (4 cm water depth). Plants were thus grown in a semi-flooded condition in which 1/3 of the pot was submerged in water and the soil above the water depth was kept moist but aerated. Water-soluble fertilizer was applied weekly until seeds were collected. Three biological replicates were included per RIL, with 12 replicates for each parental line arranged in a completely randomized design.

The hydroponic seedling tissue experiment in 2015 was performed according to our previous study (Huang et al., 2016). Briefly, an excess of seeds of each LT-RIL plus the two parents were soaked in tap water at room temperature for 2 days, drained, and allowed to continue germinating in a 37°C incubator (dark, no lights) for 1 day. Seeds selected for uniform germination per RIL or parental line (some merely pipped, others with visibly extended radicles and coleoptiles) were sown 1 seed per well in 96-well plates with the well bottoms removed to allow entry of hydroponic solution. The plates were put in a pipette tip box (12 × 8.5 × 7.5 cm) and floated in water. The boxes were covered with plastic wrap and kept in an incubator at 37°C for 1 day, and then grown with a 12 h day / 12 h night cycle in a growth chamber for the rest of the experiment. Night conditions were dark at 22°C; day conditions provided a light intensity of 700 mmol m^–2^ s^–1^ and 26°C. After 5 day in day/night culture, the seedlings were cultured with half strength Kimura B solution (0.27 mM MgSO_4_, 0.18 mM (NH_4_)_2_SO_4_, 0.18 mM Ca(NO_3_)_2_, 0.09 mM KNO_3_, 0.09 mM KH_2_PO_4_, 20 μM Na-EDTAFe, 3 μM H_3_BO_3_, 0.5 μM MnCl_2_, 0.2 μM CuSO_4_, 0.4 μM ZnSO_4_, 1 μM (NH_4_)_6_Mo_7_O_24_; pH = 5.6). The nutrient solution was renewed every 2 d. After 16 d, the nutrient solution was supplemented with 0.5 μM CoCl_2_, 0.5 μM NiCl_2_, 0.5 μM RbCl_2_, and 0.5 μM SrCl_2_ and plants were further grown for 5 day before harvesting the roots (15RTH) and shoots (15SHH) for element analysis on plants that were 21 days old counting from the initiation of seed soaking. Seedlings were washed three times with 0.5 mM CaCl_2_ solution and rinsed once with Milli-Q water, then divided into roots and shoots by cutting at the top of the crown, placed into paper envelopes, and dried at 88°C overnight. Three biological replicates were set up for each RIL and 12 replicates for each parental line. Tissue digestion occurred the day samples were removed from the drying oven.

### Tissue Elemental Analysis

Shoots and roots of LT-RILs grown hydroponically were dried at 88°C overnight, and then entire roots and shoots were weighed and digested with concentrated nitric acid at 118°C for 4 h. The elemental concentrations in the digested samples were determined using an inductively coupled plasma mass spectrometer (ICP-MS) (NexION 300D; PerkinElmer, United States). The concentrations of 16 elements in grains of LT-RILs grown on 2002, 2003, 2006, 2007, and 2008 were determined using an ICP-MS (Elan DRCe; PerkinElmer, United States) as described previously (Zhang et al., 2014), using three grains per seed harvest digested completely in nitric acid, similar to the method described below. To determine the elemental concentrations in grain of LT-RILs grown in greenhouse pots, seeds were harvested and dehusked manually. Three mature and nondiseased grains were weighed then digested with concentrated nitric acid at 118°C for 4 h and the elemental concentrations were determined by an ICP-MS (NexION 300D; PerkinElmer, United States) according to previous study (Huang et al., 2016).

### Resequencing and SNP Identification in the LT-RIL Population

The LT-RILs used for whole genome resequencing were in the F_20_ generation. The whole set of 257 LT-RILs and two parental lines TQ and LM transplanted into flooded paddies at Lingshui city, Hainan province, China in January 2017. Young leaves from one plant of each LT-RIL and parents were harvested and kept cold on dry ice until DNA was extracted. Total genomic DNA was extracted by using the cetyltrimethylammonium bromide (CTAB) method (Murray and Thompson, 1980). The integrity and quality of the genomic DNA was evaluated by electrophoresis, and the nucleic acid concentration of each sample was determined using a Qubit Fluorometer (Invitrogen, Carlsbad, CA, United States) and NanoDrop 2000 (Thermo Scientific, MA, United States). The genomic DNAs were then sheared into 500 bp fragments using an Ultrasonicator (S2/E210; Covaris, United States). The sequencing libraries were constructed from these samples and sequenced on the Illumina HiSeq 2500 platform according to the manufacturer’s instructions (Illunima, United States).

After filtered to remove adaptor and barcode sequences, clean reads were aligned to the Nipponbare reference genome (Os-Nipponbare-Reference-IRGSP-1.0^2^) using BWA software (Li and Durbin, 2009). Duplicated reads were removed using Picard^3^. SNPs and small InDels between LR-RILs and two parental lines were identified by using GATK software (McKenna et al., 2010). A total of 241.84 Gb of clean data were generated for 257 RILs and the two parents. The two pareants LM and TQ were sequenced at 65 × and 57 × coverage, respectively. The RILs were sequenced at 2 × coverage on average.

### SNP Selection and Bin Map Construction

Single nucleotide polymorphisms underwent several filterings before being used for bin map construction. SNPs that were not homozygous in both parents were discarded. SNPs in 5 bp distance or 5 bp from an InDel were discarded for a subsequent bin map construction. Additionally, the SNPs with less than 10 × sequencing depth in the parents or 4 × sequencing depth in LT-RILs were eliminated, resulting in 1,262,315 high quality SNPs. A slide window method was adopted for the bin map construction with slight modifications (Huang et al., 2009). Briefly, the genotype in each window was determined with a window size of 15 SNPs and a step size of 1 SNP. The windows containing more than 11 SNPs from TQ or LM were then defined as TQ or LM genotypes, respectively. Windows with the same continuous genotype across the entire LT-RIL population were considered as a recombination bin. Except for chromosome 4 which lacked enough bin markers in the long arm, bins with physical length < 20 kb were not included in the genetic map construction. The final set of 3,117 bin markers was used to construct the linkage map used for QTL analysis by using the HighMap program (Liu et al., 2014). Twelve linkage groups were constructed in the linkage map, corresponding to the 12 rice chromosomes.

### Element Correlation Analysis

The correlation of concentrations among 16 elements were calculated using the package *psych* in R^4^. The elemental correlation heat maps were generated by using a R package *pheatmap*^5^.

### PCA of Ionome Variation Within or Across Environments/Tissues

Principal component analysis was performed separately within or across environments/tissues. For the PCA within environment/tissue, the concentrations of 16 elements of LT-RILs in 11 environments/tissues were used, including 02GF, 03GF, 06GF, 07GF, 07GU, 08GF2, 08GU1, 08GU3, 11GGH, 15RTH, and 15SHH. PCA was performed separately on these 11 environments/tissues by using the prcomp function in the R package *psych* with the settings of retx = TRUE, center = TRUE, and scale = TRUE. In total, 16 PCs were generated from each environment/tissue. The PC scores, PC loadings, the values of biplot and screeplot were extracted from the PCA matrix results. The R packages of *ggplot2*^6^ and *ggfortify*^7^ were used to generated the biplots in Figure 5 and Supplementary Figure 5. The eigen function in the *ggfortify* package was used to generate the screeplots in Figures 5A,B.

For the PCA across environments/tissues, the concentrations of 16 elements in grains, shoots or roots of LT-RILs grown under different environments were used, including elemental concentrations in grains of LT-RILs grown under the flooded condition (02GF, 03GF, 06GF, 07GF, and 08GF2; F), unflooded condition (07GU, 08GU1, and 08GU3; U), the combined flooded, unflooded and semi-flooded conditions in greenhouse (F, U and 11GGH; FUGG) and the concentrations 16 elements in greenhouse-grown grains, roots and shoots (FUGG, 15RTH and 15SHH; GRS). PCA was performed separately on the environments of F, U, FUGG and GRS by using the prcomp function with the same settings as above. The extraction of PC scores and the generation of biplots and screeplots were conducted as above.

### QTL Analysis

To detect the elemental QTLs, the concentrations of 16 elements of LT-RILs in 11 environments/tissues were used as phenotype data, including 02GF, 03GF, 06GF, 07GF, 07GU, 08GF2, 08GU1, 08GU3, 11GGH, 15RTH, and 15SHH. The LS means of grain elemental concentrations of 02GF, 03GF and 06GF (02-06_mean), LS means of 07GU, 8U1, and 08GU3 (U_mean), and LS means of 02GF, 03GF, 06GF, 07GF and 08GF2 (F_mean) were also used as traits for detection of elemental QTLs. The PC scores of 16 PCs in each individual environment/tissue were used as phenotype values for QTL analysis to detect the PC-QTLs. For the detection of aPC-QTLs, PC scores derived from PCA across environments/tissues were used as traits for QTL analysis, including across the flooded condition (02GF, 03GF, 06GF, 07GF, and 08GF2; F), unflooded condition (07GU, 8U1, and 08GU3; U), the combined flooded, unflooded and semi-flooded conditions in greenhouse (F, U and 11GGH; FUGG) and across grain, root and shoot tissues (FUGG, 15RTH and 15SHH; GRS). QTL analysis was performed using the R/qtl package with a 1-cM step length and a 10-cM window size^8^ (Broman et al., 2003). The imputation method in the R/qtl package was used by the setting of method = “imp”. The LOD threshold value was estimated by 1,000 permutations with a significance level of *p* = 0.05. The distribution of QTLs in the genetic linkage map in Figure 3C was generated by using the TBtools software (Chen et al., 2020).

### Identification of Candidate Genes

To identify the candidate genes for the QTLs detected, the functional annotations of rice genes were retrieved from Rice Annotation Project Database^9^ or Rice Genome Annotation Project^10^. The candidate genes were selected from the genes in the QTL mapping interval based on gene annotations. To identify the sequence polymorphisms of the candidate genes for the QTL clusters, the reads of TQ and LM were mapped the Nipponbare reference genome to the SNPs and indels were called. The nonsynonymous SNPs and the indels in the coding region or the indels in the promoter regions were considered as potential functional polymorphisms.

## Data Availability Statement

The datasets presented in this study can be found in online repositories. The names of the repository/repositories and accession number(s) can be found below: https://www.ncbi.nlm.nih.gov/, PRJNA687339.

## Author Contributions

X-YH designed the research and wrote the manuscript with contributions from SP, MG, DS, and F-JZ. X-YH, HL, S-XL, and SP performed the experiments. X-YH, HL, and ZT analyzed the data. X-YH, F-JZ, DS, MG, and SP provided resources. All authors have read and agreed to the published version of the manuscript.

## Conflict of Interest

The authors declare that the research was conducted in the absence of any commercial or financial relationships that could be construed as a potential conflict of interest.

## Footnotes

1 www.ars.usda.gov/GSOR

2 https://rapdb.dna.affrc.go.jp/download/irgsp1.html

3 http://sourceforge.net/projects/picard/

4 https://cran.r-project.org/web/packages/psych

5 https://cran.r-project.org/web/packages/pheatmap/index.html

6 https://cran.r-project.org/web/packages/ggplot2/index.html

7 https://cran.r-project.org/web/packages/ggfortify/index.html

8 https://rqtl.org/

9 https://rapdb.dna.affrc.go.jp/

10 http://rice.plantbiology.msu.edu/

## References

[B1] Baxter I. (2015). Should we treat the ionome as a combination of individual elements, or should we be deriving novel combined traits? *J. Exp. Bot.* 66 2127–2131. 10.1093/jxb/erv040 25711709 PMC4986723

[B2] Bouchet S. Bertin P. Presterl T. Jamin P. Coubriche D. Gouesnard B. (2017). Association mapping for phenology and plant architecture in maize shows higher power for developmental traits compared with growth influenced traits. *Heredity* 118 249–259. 10.1038/hdy.2016.88 27876803 PMC5315527

[B3] Broman K. W. Wu H. Sen S. Churchill G. A. (2003). R/qtl: QTL mapping in experimental crosses. *Bioinformatics* 19 889–890. 10.1093/bioinformatics/btg112 12724300

[B4] Chen C. Chen H. Zhang Y. Thomas H. R. Frank M. H. He Y. (2020). TBtools: an integrative toolkit developed for interactive analyses of big biological data. *Mol. Plant* 13 1194–1202. 10.1016/j.molp.2020.06.009 32585190

[B5] Chen J. Zou W. Meng L. Fan X. Xu G. Ye G. (2019). Advances in the uptake and transport mechanisms and QTLs mapping of cadmium in rice. *Int. J. Mol. Sci.* 20 3417. 10.3390/ijms20143417 31336794 PMC6678204

[B6] Choe E. Rocheford T. R. (2012). Genetic and QTL analysis of pericarp thickness and ear architecture traits of Korean waxy corn germplasm. *Euphytica* 183 243–260. 10.1007/s10681-011-0452-8

[B7] Doblas V. G. Geldner N. Barberon M. (2017). The endodermis, a tightly controlled barrier for nutrients. *Curr. Opin. Plant Biol.* 39 136–143. 10.1016/j.pbi.2017.06.010 28750257

[B8] Fikas A. A. Dilkes B. P. Baxter I. (2019). Multivariate analysis reveals environmental and genetic determinants of element covariation in the maize grain ionome. *Plant Direct* 3 e00139. 10.1002/pld3.139 31245778 PMC6589523

[B9] Frossard E. Bucher M. Machler F. Mozafar A. Hurrell R. (2000). Potential for increasing the content and bioavailability of Fe, Zn and Ca in plants for human nutrition. *J. Sci. Food Agric.* 80 861–879. 10.1002/(sici)1097-0010(20000515)80:7<861::aid-jsfa601<3.0.co;2-p

[B10] Geldner N. (2013). The endodermis. *Annu. Rev. Plant Biol.* 64 531–558. 10.1146/annurev-arplant-050312-120050 23451777

[B11] Hosmani P. S. Kamiya T. Danku J. Naseer S. Geldner N. Guerinot M. L. (2013). Dirigent domain-containing protein is part of the machinery required for formation of the lignin-based Casparian strip in the root. *Proc. Natl. Acad. Sci. U.S.A.* 110 14498–14503. 10.1073/pnas.1308412110 23940370 PMC3761638

[B12] Huang X. Feng Q. Qian Q. Zhao Q. Wang L. Wang A. (2009). High-throughput genotyping by whole-genome resequencing. *Genome Res.* 19 1068–1076. 10.1101/gr.089516.108 19420380 PMC2694477

[B13] Huang X. Y. Deng F. Yamaji N. Pinson S. R. Fujii-Kashino M. Danku J. (2016). A heavy metal P-type ATPase OsHMA4 prevents copper accumulation in rice grain. *Nat. Commun.* 7 12138. 10.1038/ncomms12138 27387148 PMC4941113

[B14] Huang X. Y. Liu H. Zhu Y. F. Pinson S. R. M. Lin H. X. Guerinot M. L. (2019). Natural variation in a molybdate transporter controls grain molybdenum concentration in rice. *New Phytol.* 221 1983–1997. 10.1111/nph.15546 30339276

[B15] Huang Y. Sun C. Min J. Chen Y. Tong C. Bao J. (2015). Association mapping of quantitative trait loci for mineral element contents in whole grain rice (*Oryza sativa* L.). *J. Agric. Food Chem.* 63 10885–10892. 10.1021/acs.jafc.5b04932 26641542

[B16] Ishikawa A. Namikawa T. (2004). Mapping major quantitative trait loci for postnatal growth in an intersubspecific backcross between C57BL/6J and Philippine wild mice by using principal component analysis. *Genes Genet. Syst.* 79 27–39. 10.1266/ggs.79.27 15056934

[B17] Ishikawa S. Ishimaru Y. Igura M. Kuramata M. Abe T. Senoura T. (2012). Ion-beam irradiation, gene identification, and marker-assisted breeding in the development of low-cadmium rice. *Proc. Natl. Acad. Sci. U.S.A.* 109 19166–19171. 10.1073/pnas.1211132109 23132948 PMC3511095

[B18] Ishimaru Y. Takahashi R. Bashir K. Shimo H. Senoura T. Sugimoto K. (2012). Characterizing the role of rice NRAMP5 in manganese, iron and cadmium transport. *Sci. Rep.* 2 286. 10.1038/srep00286 22368778 PMC3285952

[B19] Kamiya T. Borghi M. Wang P. Danku J. M. Kalmbach L. Hosmani P. S. (2015). The MYB36 transcription factor orchestrates Casparian strip formation. *Proc. Natl. Acad. Sci. U.S.A.* 112 10533–10538. 10.1073/pnas.1507691112 26124109 PMC4547244

[B20] Korshunova Y. O. Eide D. Clark W. G. Guerinot M. L. Pakrasi H. B. (1999). The IRT1 protein from *Arabidopsis thaliana* is a metal transporter with a broad substrate range. *Plant Mol. Biol.* 40 37–44. 10.1023/a:1026438615520 10394943

[B21] Lahner B. Gong J. Mahmoudian M. Smith E. L. Abid K. B. Rogers E. E. (2003). Genomic scale profiling of nutrient and trace elements in *Arabidopsis thaliana*. *Nat. Biotechnol.* 21 1215–1221. 10.1038/nbt865 12949535

[B22] Li B. Kamiya T. Kalmbach L. Yamagami M. Yamaguchi K. Shigenobu S. (2017). Role of LOTR1 in nutrient transport through organization of spatial distribution of root endodermal barriers. *Curr. Biol.* 27 758–765. 10.1016/j.cub.2017.01.030 28238658

[B23] Li G. Sun G. X. Williams P. N. Nunes L. Zhu Y. G. (2011). Inorganic arsenic in Chinese food and its cancer risk. *Environ. Int.* 37 1219–1225. 10.1016/j.envint.2011.05.007 21632110

[B24] Li H. Durbin R. (2009). Fast and accurate short read alignment with Burrows-Wheeler transform. *Bioinformatics* 25 1754–1760. 10.1093/bioinformatics/btp324 19451168 PMC2705234

[B25] Liu D. Ma C. Hong W. Huang L. Liu M. Liu H. (2014). Construction and analysis of high-density linkage map using high-throughput sequencing data. *PLoS One* 9:e98855. 10.1371/journal.pone.0098855 24905985 PMC4048240

[B26] Liu X. Fan F. Liu M. Long W. Yu Y. Yuan H. (2020). Quantitative trait loci mapping of mineral element contents in brown rice using backcross inbred lines derived from *Oryza longistaminata*. *Front. Plant Sci.* 11:1229. 10.3389/fpls.2020.01229 32903403 PMC7434966

[B27] Liu Z. Garcia A. Mcmullen M. Flint-Garcia S. (2016). Genetic analysis of kernel traits in maize-teosinte introgression populations. *G3* 6 2523–2530. 10.1534/g3.116.030155 27317774 PMC4978905

[B28] Luo J. S. Huang J. Zeng D. L. Peng J. S. Zhang G. B. Ma H. L. (2018). A defensin-like protein drives cadmium efflux and allocation in rice. *Nat. Commun.* 9 645. 10.1038/s41467-018-03088-0 29440679 PMC5811569

[B29] McKenna A. Hanna M. Banks E. Sivachenko A. Cibulskis K. Kernytsky A. (2010). The genome analysis toolkit: a MapReduce framework for analyzing next-generation DNA sequencing data. *Genome Res.* 20 1297–1303. 10.1101/gr.107524.110 20644199 PMC2928508

[B30] Miyadate H. Adachi S. Hiraizumi A. Tezuka K. Nakazawa N. Kawamoto T. (2011). OsHMA3, a P1B-type of ATPase affects root-to-shoot cadmium translocation in rice by mediating efflux into vacuoles. *New Phytol.* 189 190–199. 10.1111/j.1469-8137.2010.03459.x 20840506

[B31] Murray M. G. Thompson W. F. (1980). Rapid isolation of high molecular weight plant DNA. *Nucleic Acids Res.* 8 4321–4325. 10.1093/nar/8.19.4321 7433111 PMC324241

[B32] Norton G. J. Deacon C. M. Xiong L. Z. Huang S. Y. Meharg A. A. Price A. H. (2010). Genetic mapping of the rice ionome in leaves and grain: identification of QTLs for 17 elements including arsenic, cadmium, iron and selenium. *Plant Soil* 329 139–153. 10.1007/s11104-009-0141-8

[B33] Norton G. J. Douglas A. Lahner B. Yakubova E. Guerinot M. L. Pinson S. R. (2014). Genome wide association mapping of grain arsenic, copper, molybdenum and zinc in rice (*Oryza sativa* L.) grown at four international field sites. *PLoS One* 9:e89685. 10.1371/journal.pone.0089685 24586963 PMC3934919

[B34] Norton G. J. Travis A. J. Talukdar P. Hossain M. Islam M. R. Douglas A. (2019). Genetic loci regulating arsenic content in rice grains when grown flooded or under alternative wetting and drying irrigation. *Rice* 12 54. 10.1186/s12284-019-0307-9 31332547 PMC6646650

[B35] Pinson S. R. M. Capdevielle F. M. Oard J. H. (2005). Confirming QTLs and finding additional loci conditioning sheath blight resistance in rice using recombinant inbred lines. *Crop Sci.* 45 503–510. 10.2135/cropsci2005.0503

[B36] Pinson S. R. M. Shahjahan A. K. M. Rush M. C. Groth D. E. (2010). Bacterial panicle blight resistance QTLs in rice and their association with other disease resistance loci and heading date. *Crop Sci.* 50 1287–1297. 10.2135/cropsci2008.07.0447

[B37] Pinson S. R. M. Tarpley L. Yan W. G. Yeater K. Lahner B. Yakubova E. (2015). Worldwide genetic diversity for mineral element concentrations in rice grain. *Crop Sci.* 55 294–311. 10.2135/cropsci2013.10.0656

[B38] Raza Q. Riaz A. Sabar M. Atif R. M. Bashir K. (2019). Meta-analysis of grain iron and zinc associated QTLs identified hotspot chromosomal regions and positional candidate genes for breeding biofortified rice. *Plant Sci.* 288 110214. 10.1016/j.plantsci.2019.110214 31521222

[B39] Ringner M. (2008). What is principal component analysis? *Nat. Biotechnol.* 26 303–304. 10.1038/nbt0308-303 18327243

[B40] Rude R. Gruber H. (2004). Magnesium deficiency and osteoporosis: animal and human observations. *J. Nutr. Biochem.* 15 710–716. 15607643 10.1016/j.jnutbio.2004.08.001

[B41] Salt D. E. Baxter I. Lahner B. (2008). Ionomics and the study of the plant ionome. *Annu. Rev. Plant Biol.* 59 709–733. 10.1146/annurev.arplant.59.032607.092942 18251712

[B42] Sasaki A. Yamaji N. Yokosho K. Ma J. F. (2012). Nramp5 is a major transporter responsible for manganese and cadmium uptake in rice. *Plant Cell* 24 2155–2167. 10.1105/tpc.112.096925 22589467 PMC3442593

[B43] Song Y. Wang Y. Mao W. Sui H. Yong L. Yang D. (2017). Dietary cadmium exposure assessment among the Chinese population. *PLoS One* 12:e0177978. 10.1371/journal.pone.0177978 28542445 PMC5436861

[B44] Sors T. G. Ellis D. R. Salt D. E. (2005). Selenium uptake, translocation, assimilation and metabolic fate in plants. *Photosynth Res.* 86 373–389. 10.1007/s11120-005-5222-9 16307305

[B45] Sui F. Zhao D. Zhu H. Gong Y. Tang Z. Huang X. Y. (2019). Map-based cloning of a new total loss-of-function allele of OsHMA3 causes high cadmium accumulation in rice grain. *J. Exp. Bot.* 70 2857–2871. 10.1093/jxb/erz093 30840768

[B46] Tabien E. Li Z. Paterson H. Marchetti A. Stansel W. Pinson M. (2002). Mapping QTLs for field resistance to the rice blast pathogen and evaluating their individual and combined utility in improved varieties. *Theor. Appl. Genet.* 105 313–324. 10.1007/s00122-002-0940-2 12582534

[B47] Tabien R. E. Li Z. Paterson A. H. Marchetti M. A. Stansel J. W. Pinson S. R. M. (2000). Mapping of four major rice blast resistance genes from ‘Lemont’ and ‘Teqing’ and evaluation of their combinatorial effect for field resistance. *Theor. Appl. Genet.* 101 1215–1225. 10.1007/s001220051600

[B48] Tan Y. Sun L. Song Q. Mao D. Zhou J. Jiang Y. (2020). Genetic architecture of subspecies divergence in trace mineral accumulation and elemental correlations in the rice grain. *Theor. Appl. Genet.* 133 529–545. 10.1007/s00122-019-03485-z 31734869

[B49] Thacher T. Fischer P. Strand M. Pettifor J. (2006). Nutritional rickets around the world: causes and future directions. *Ann. Trop. Paediatr.* 26 1–16. 16494699 10.1179/146532806X90556

[B50] Topp C. N. Iyer-Pascuzzi A. S. Anderson J. T. Lee C. R. Zurek P. R. Symonova O. (2013). 3D phenotyping and quantitative trait locus mapping identify core regions of the rice genome controlling root architecture. *Proc. Natl. Acad. Sci. U.S.A.* 110 E1695–E1704. 10.1073/pnas.1304354110 23580618 PMC3645568

[B51] Ueno D. Yamaji N. Kono I. Huang C. F. Ando T. Yano M. (2010). Gene limiting cadmium accumulation in rice. *Proc. Natl. Acad. Sci. U.S.A.* 107 16500–16505. 10.1073/pnas.1005396107 20823253 PMC2944702

[B52] Wang C. Tang Z. Zhuang J. Y. Tang Z. Huang X. Y. Zhao F. J. (2020). Genetic mapping of ionomic quantitative trait loci in rice grain and straw reveals OsMOT1;1 as the putative causal gene for a molybdenum QTL qMo8. *Mol. Genet. Genomics* 295 391–407. 10.1007/s00438-019-01632-1 31797032

[B53] Welch R. M. Graham R. D. (2005). Agriculture: the real nexus for enhancing bioavailable micronutrients in food crops. *J. Trace Elem. Med. Biol.* 18 299–307. 10.1016/j.jtemb.2005.03.001 16028491

[B54] White P. J. Broadley M. R. (2009). Biofortification of crops with seven mineral elements often lacking in human diets–iron, zinc, copper, calcium, magnesium, selenium and iodine. *New Phytol.* 182 49–84. 10.1111/j.1469-8137.2008.02738.x 19192191

[B55] Yan H. Xu W. Xie J. Gao Y. Wu L. Sun L. (2019). Variation of a major facilitator superfamily gene contributes to differential cadmium accumulation between rice subspecies. *Nat. Commun.* 10 2562. 10.1038/s41467-019-10544-y 31189898 PMC6561962

[B56] Yang M. Lu K. Zhao F. J. Xie W. Ramakrishna P. Wang G. (2018). Genome-wide association studies reveal the genetic basis of ionomic variation in rice. *Plant Cell* 30 2720–2740. 10.1105/tpc.18.00375 30373760 PMC6305983

[B57] Yang M. Zhang Y. Zhang L. Hu J. Zhang X. Lu K. (2014). OsNRAMP5 contributes to manganese translocation and distribution in rice shoots. *J. Exp. Bot.* 65 4849–4861. 10.1093/jxb/eru259 24963001 PMC4144776

[B58] Yano K. Morinaka Y. Wang F. Huang P. Takehara S. Hirai T. (2019). GWAS with principal component analysis identifies a gene comprehensively controlling rice architecture. *Proc. Natl. Acad. Sci. U.S.A.* 116 21262–21267. 10.1073/pnas.1904964116 31570620 PMC6800328

[B59] Yu Y. H. Shao Y. F. Liu J. Fan Y. Y. Sun C. X. Cao Z. Y. (2015). Mapping of quantitative trait loci for contents of macro- and microelements in milled rice (*Oryza sativa* L.). *J. Agric. Food Chem.* 63 7813–7818. 10.1021/acs.jafc.5b02882 26301991

[B60] Zhang M. Pinson S. R. Tarpley L. Huang X. Y. Lahner B. Yakubova E. (2014). Mapping and validation of quantitative trait loci associated with concentrations of 16 elements in unmilled rice grain. *Theor. Appl. Genet.* 127 137–165. 10.1007/s00122-013-2207-5 24231918 PMC4544570

[B61] Zhang N. Gibon Y. Gur A. Chen C. Lepak N. Hohne M. (2010). Fine quantitative trait loci mapping of carbon and nitrogen metabolism enzyme activities and seedling biomass in the maize IBM mapping population. *Plant Physiol.* 154 1753–1765. 10.1104/pp.110.165787 20971858 PMC2996021

[B62] Zhao F. J. Ma Y. Zhu Y. G. Tang Z. Mcgrath S. P. (2015). Soil contamination in China: current status and mitigation strategies. *Environ. Sci. Technol.* 49 750–759. 10.1021/es5047099 25514502

