# Supplementary material for: Univariate and Multivariate QTL Analyses Reveal Covariance Among Mineral Elements in the Rice Ionome
Source: Front Genet. 2021 Jan 25;12:638555. doi: 10.3389/fgene.2021.638555 (PMC7868434; doi:10.3389/fgene.2021.638555)
Supplement: Supplementary file 1 [file Data_Sheet_1.PDF]

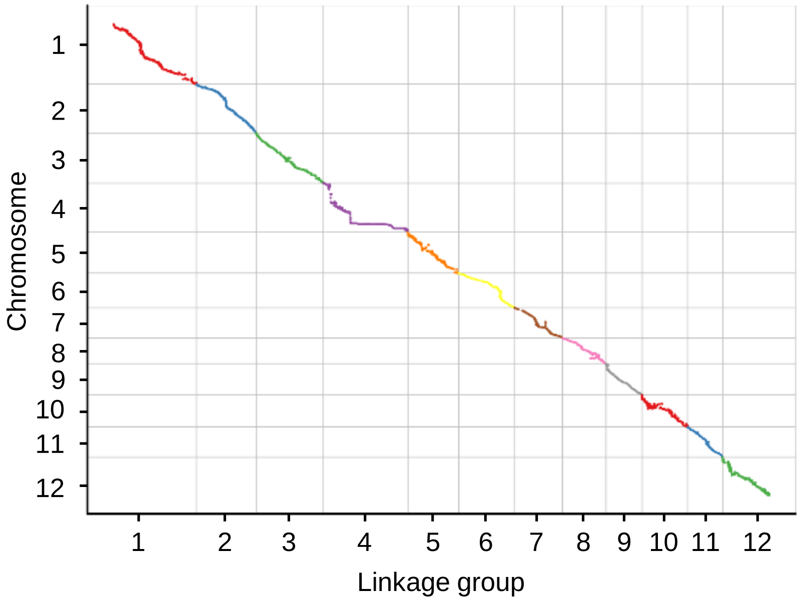

Supplementary Figure 1. Collinearity analysis of linkage groups with rice reference genome. The x-axis shows the linear order and genetic distances of bin markers on each linkage groups, while the y-axis represents the physical position of rice reference genome. Different colors indicate different chromosomes.

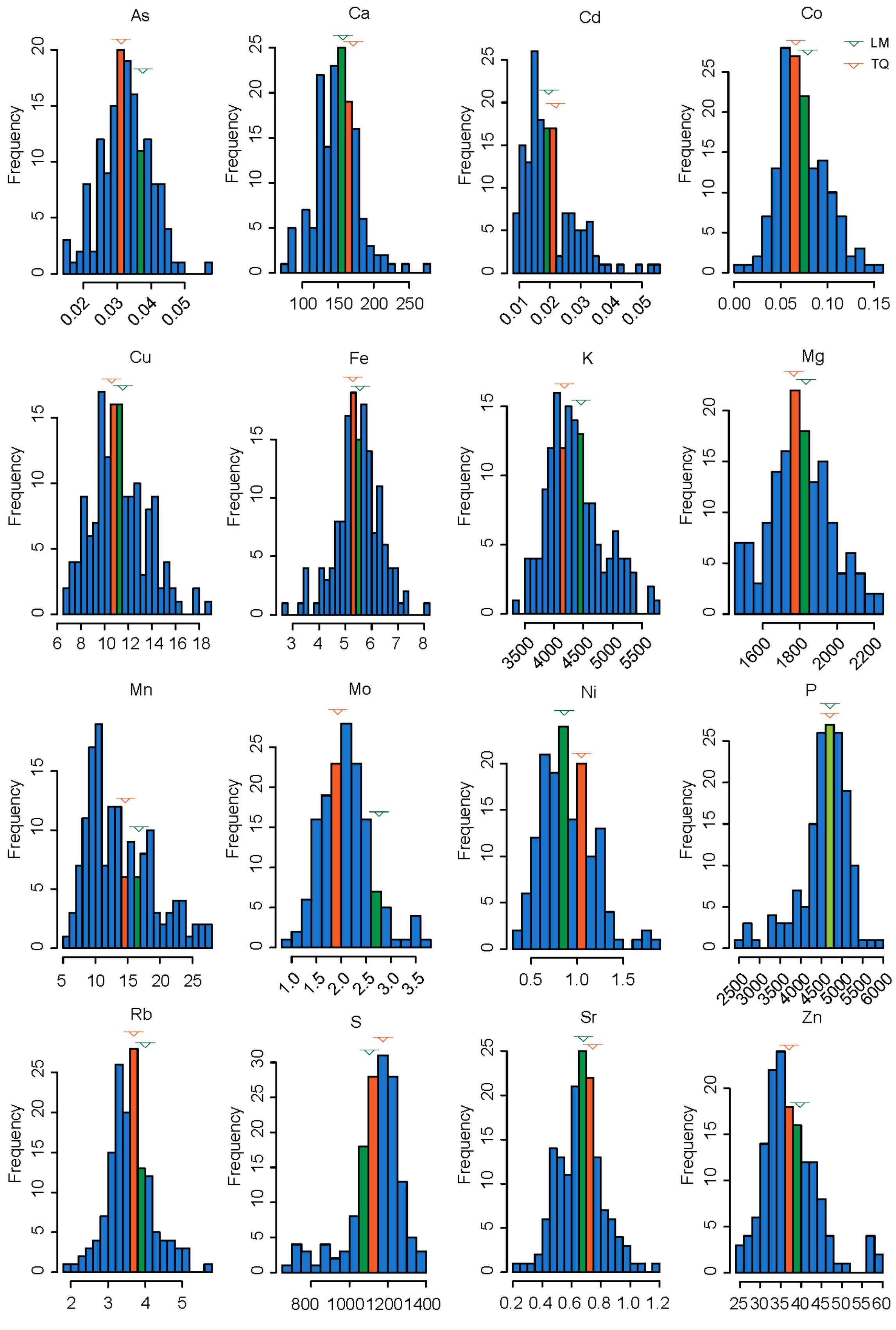

Supplementary Figure 2. Frequency distribution of 16 elements in grains of LT-RIL population grown in a greenhouse under semi-flooded condition (11GGH). The inverted triangles represent the range of elemental concentrations of two parents. The units for all elements are µg g^{-1} dry weight. TQ, TeQing; LM, Lemont.

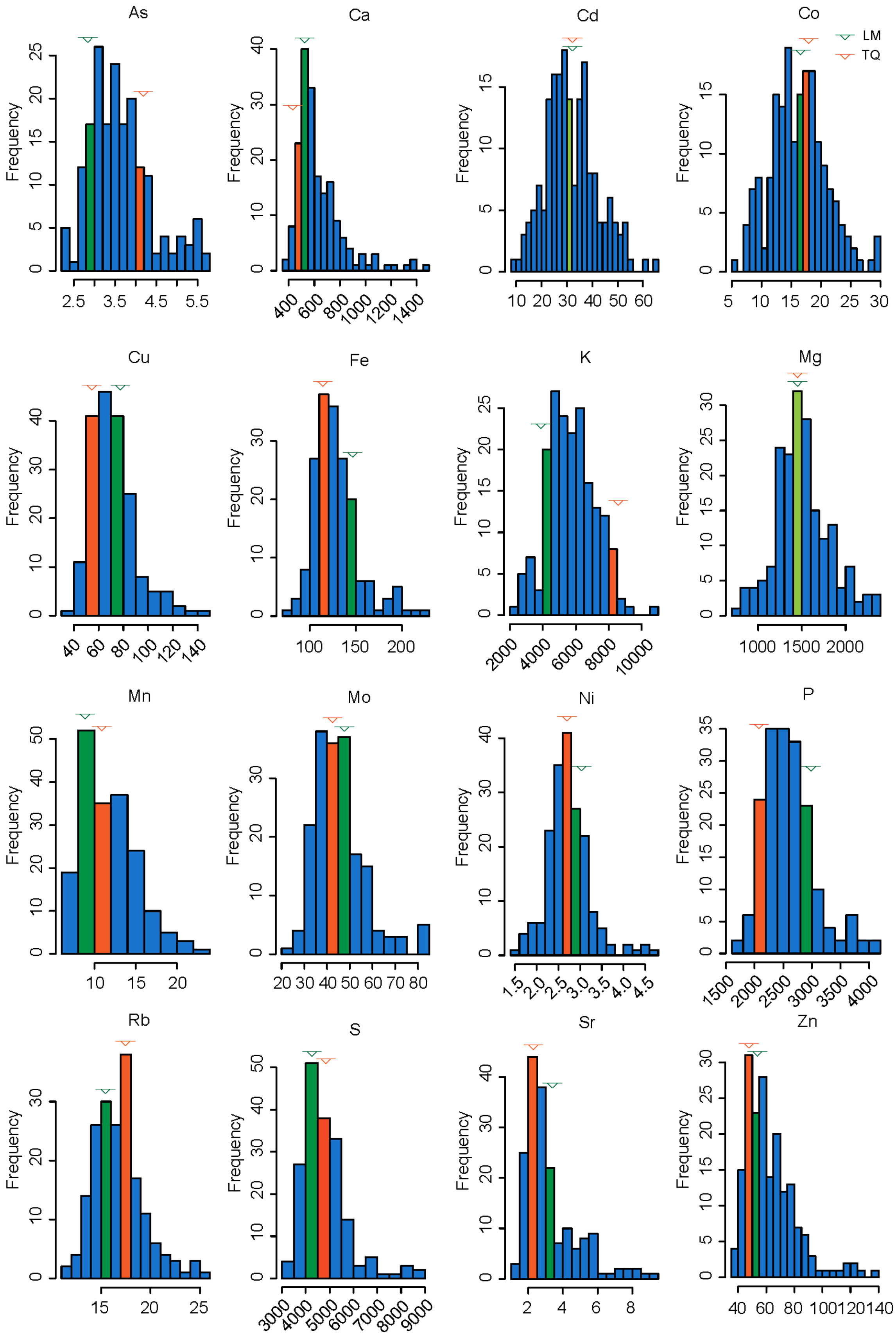

Supplementary Figure 3. Frequency distribution of 16 elements in roots of LT-RIL population grown hydroponically (15RTH). The inverted triangles represent the range of elemental concentrations of two parents. The units for all elements are μg g^{-1} dry weight. TQ, TeQing; LM, Lemont.

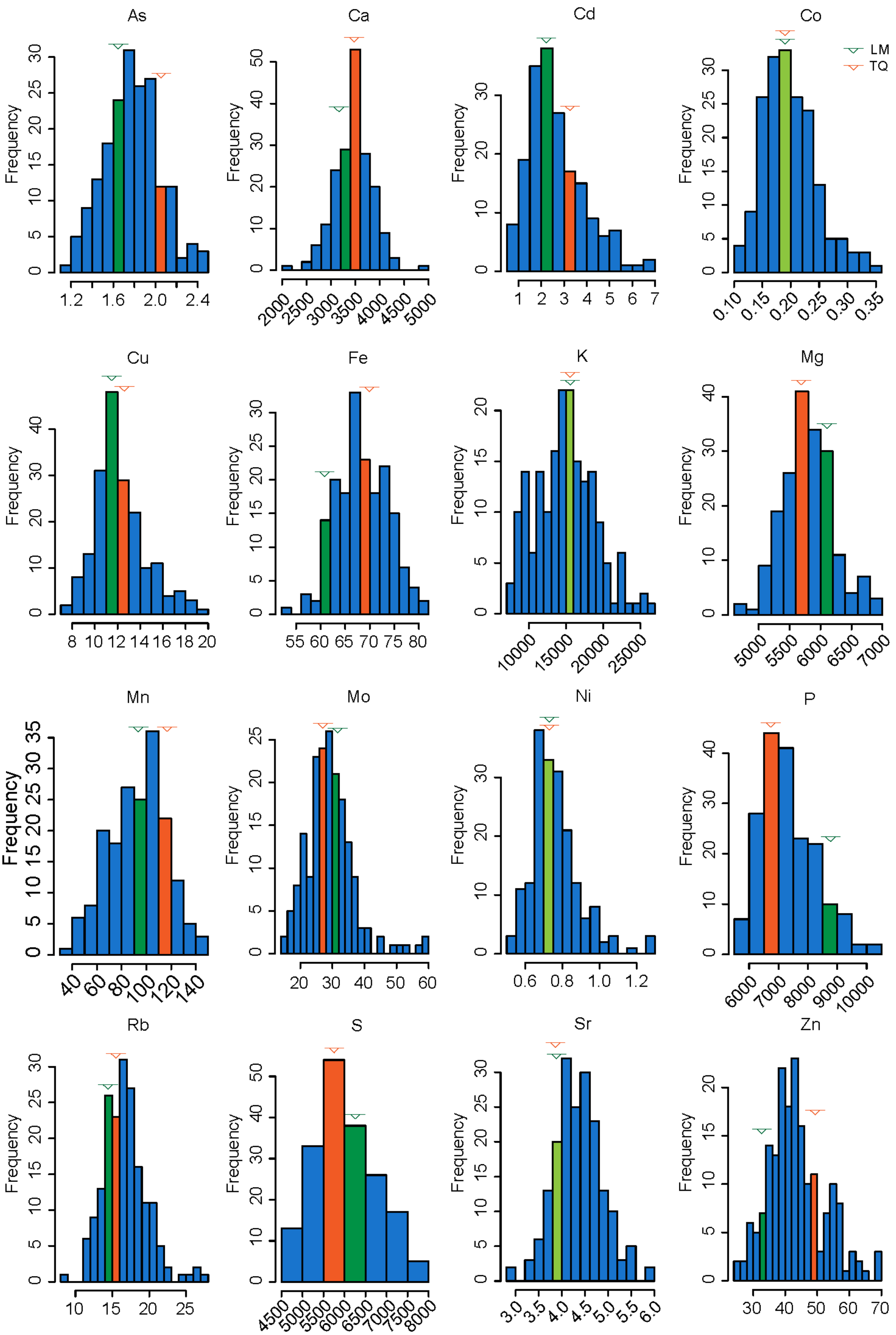

Supplementary Figure 4. Frequency distribution of 16 elements in shoots of LT-RIL population grown hydroponically (15SHH). The inverted triangles represent the range of elemental concentrations of two parents. The units for all elements are μg g^{-1} dry weight. TQ, TeQing; LM, Lemont.

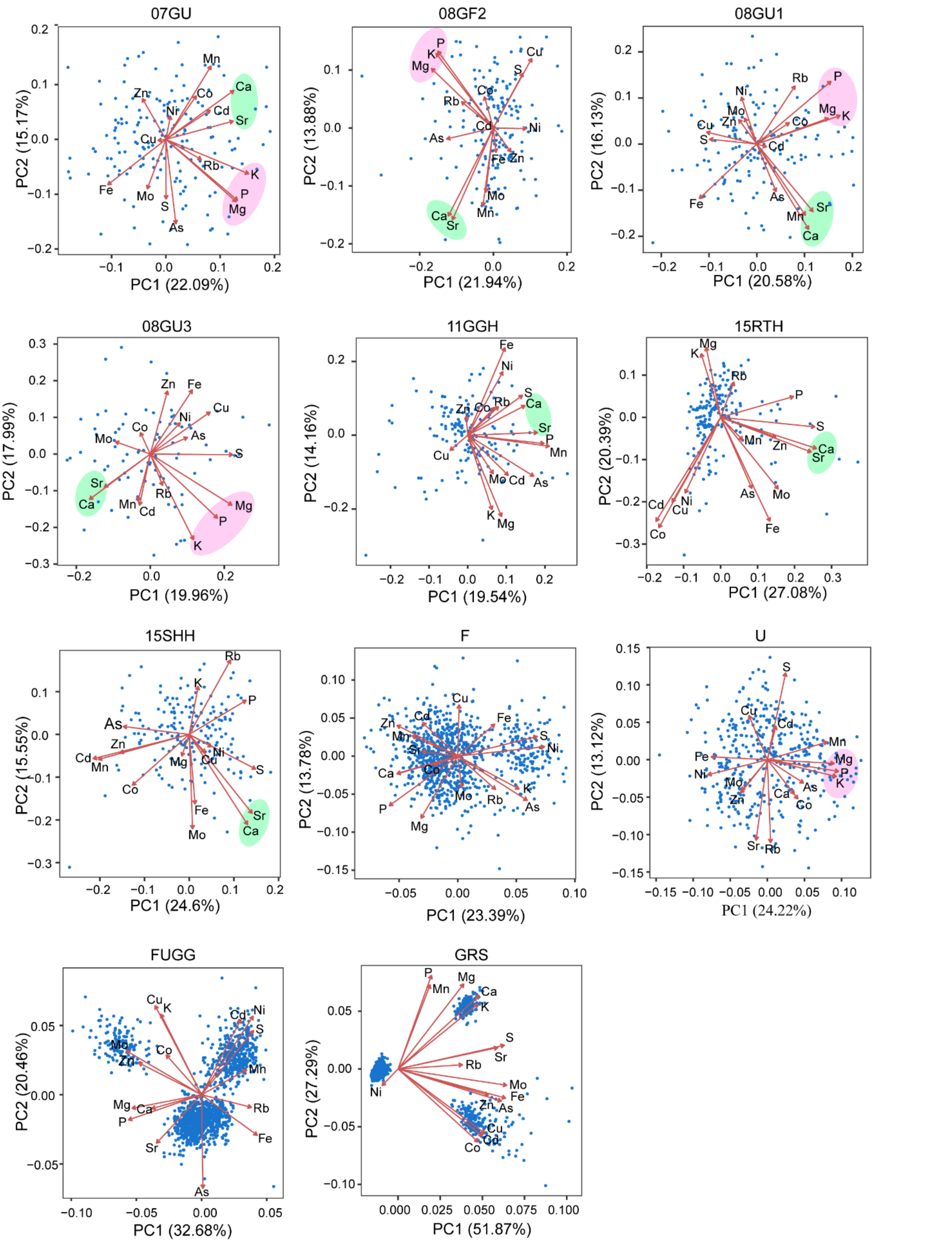

Supplementary Figure 5. Biplots of PC1 and PC2 loadings of each element in different environments/tissues. The element pairs The correlated elements with similar PC direction and magnitude were highlighted in color background. 07GU is grains (G) of RILs grown under unflooded (U) condition on 2007; 08GF2, 08GU1 and 08GU3 are grains (G) of RILs grown under flooded (F) or unflooded (U) condition on 2008 in the field site number 2, 1 and 3; 11GGH is the grains (G) of RILs grown in a greenhouse (GH) condition on 2011; 15RTH and 15SHH are roots (RT) or shoots (SH) of RILs grown hydroponically (H) on 2015. F: 02GF+03GF+06GF+07GF+08GF2; U: 07GU+08GU1+08GU3; FUGG: F+U+11GGH; GRS: FUGG+15RTH+15SHH.

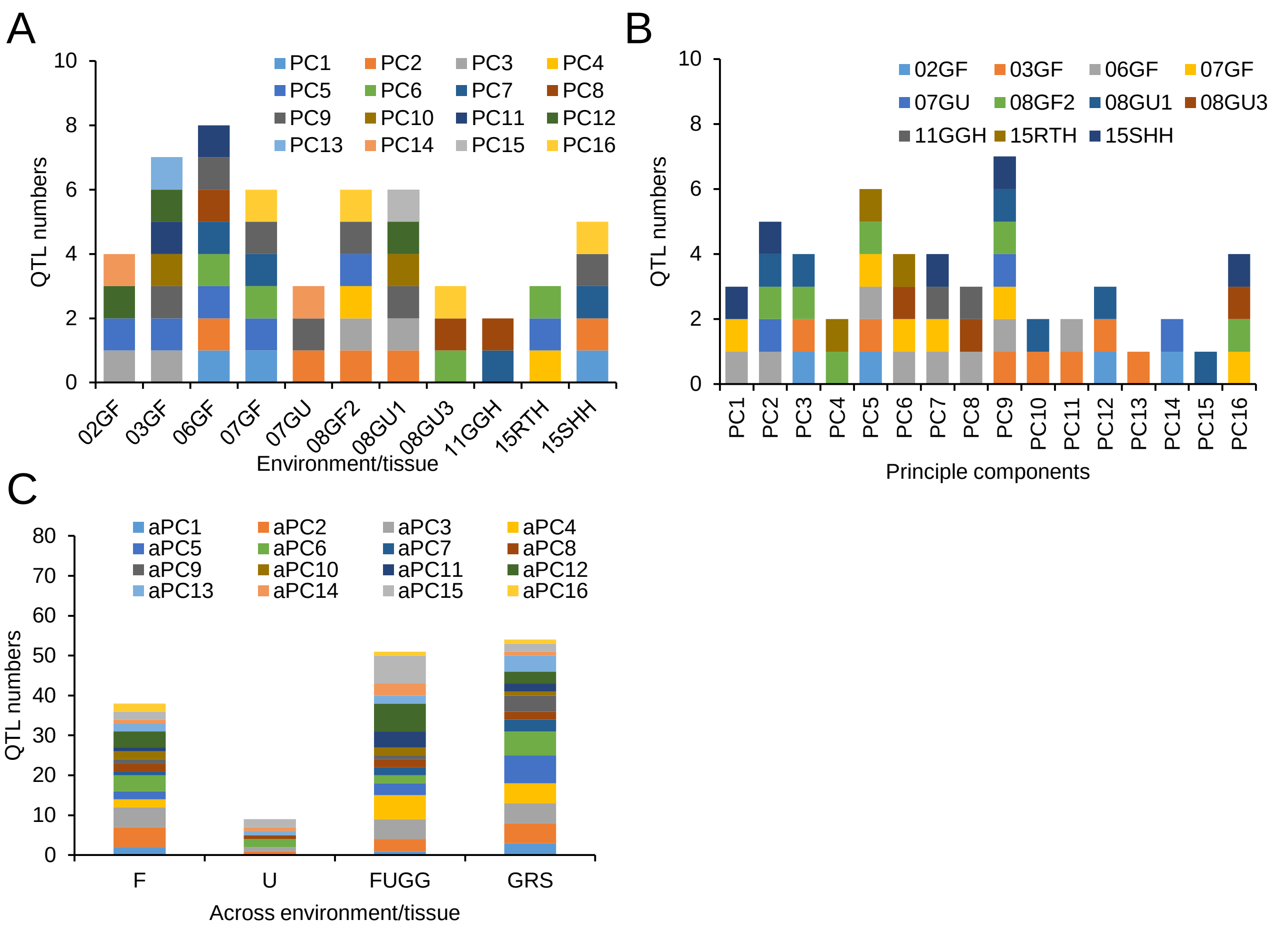

Supplementary Figure 6. Total numbers of PC-QTLs and aPC-QTLs. (a) Total number of PC-QTLs detected in each individual environment or tissue. (b) Total number of PC-QTLs detected for each PCs. (c) Total number of aPC-QTLs detected in grains of LT-RILs across different environments or tissues. 02GF, 03GF, 06GF, 07GF and 07GU are grains (G) of RILs grown under flooded (F) or unflooded (U) conditions on 2002, 2003, 2006 and 2007, respectively; 08GF2, 08GU1 and 08GU3 are grains (G) of RILs grown under flooded (F) or unflooded (U) condition on 2008 in the field site number 2, 1 and 3; 11GGH is the grains (G) of RILs grown in a greenhouse (GH) condition on 2011; 15RTH and 15SHH are roots (RT) or shoots (SH) of RILs grown hydroponically (H) on 2015.

Supplementary Table 1. Sequencing information of RIL population and two parental lines

Sample ID	Total clean reads	Total clean bases	Mapped (%)[a]	Sequencing depth	Cov_ratio_1X (%)[b]
LM	81,598,379	24,447,555,372	97.61	65.5	95.59
TQ	71,300,065	21,361,553,496	96.9	57.23	92.19
RILs	654,348,320	196,030,343,786	96.91	2.04	64.22

[a] Percentage of clean reads being mapped to the Nipponbare reference genome.

[b] Percentage of genomic region that is covered at least at 1× sequencing depth.

Supplementary Table 2. Physical distance of bins on each chromosome

Chr.	No. of SNPs	No. of bins	Average physical distance (Kb)
1	34,587	380	39
2	48,524	372	37
3	36,447	349	40
4	9,877	156	27
5	33,811	284	58
6	43,610	287	42
7	21,732	207	41
8	17,004	213	40
9	29,745	218	42
10	23,615	229	44
11	11,367	144	39
12	28,348	278	49

Supplementary Table 3. Genetic distance of bins on each chromosome

Chr.	No. of bins	Total distance(cM)	Average distance(cM)
1	380	222.06	0.59
2	372	159.3	0.43
3	349	179.9	0.52
4	156	226.43	1.46
5	284	135.94	0.48
6	287	149.54	0.52
7	207	128.13	0.62
8	213	116.36	0.55
9	218	96.58	0.45
10	229	121.76	0.53
11	144	93.82	0.66
12	278	125.05	0.45
Total	3,117	1754.87	0.61

Supplementary Table 4. Information of traits used for QTL analysis on elemental concentrations.

Symbol	Year	Tissue	Growth condition	Note
02GF	2002	Grain	Field grown, flooded	Staggered planting
03GF	2003	Grain	Field grown, flooded	Staggered planting
06GF	2006	Grain	Field grown, flooded	Staggered planting
07GF	2007	Grain	Field grown, flooded	-
07GU	2007	Grain	Field grown, unflooded	-
08GF2	2008	Grain	Field grown, flooded	Field plot #2
08GU1	2008	Grain	Field grown, unflooded	Field plot #1
08GU3	2008	Grain	Field grown, unflooded	Field plot #3
11GGH	2011	Grain	Greenhouse, semi-flooded	-
15RTH	2015	Root	Grown hydroponically	-
15SHH	2015	Shoot	Grown hydroponically	-
U_mean	-	Grain	Field grown, unflooded	Least squares mean of 07GU, 08GU1 and 08GU3
02-06_mean	-	Grain	Field grown, flooded	Least squares mean of 02GF, 03GF and 06GF
F_mean	-	Grain	Field grown, flooded	Least squares mean of 02GF, 03GF, 06GF, 07GF and 08GF2

Supplementary Table 5. Analysis of variance (ANOVA) of concentrations of 16 elements in the grains, roots and shoots of LT-RIL population.

Element	Source of variation	11GGH[1]					15RTH[2]					15SHH[3]				
		SS	DF	MS	F	*p* -value	SS	DF	MS	F	*p* -value	SS	DF	MS	F	*p* -value
As	Group	0.023	150	0.000152	2.556	3.22E-12	187.035	150	1.247	2.139	4.04E-08	22.79	150	0.152	4.305	1.85E-23
	Error	0.018	298	0.00006			134.063	230	0.583			8.117	230	0.035		
	Total	0.041	448				321.097	380				30.908	380			
Ca	Group	392452.413	150	2616.349	1.28	0.038	7204632.491	150	48030.88	1.204516	0.102079	48512501.51	150	323416.7	2.159	6.97E-08
	Error	609122.983	298	2044.037			9171401.777	230	39875.66			34459694.41	230	149824.8		
	Total	1001575.395	448				16376034.27	380				82972195.92	380			
Cd	Group	0.031	150	0.00021	3.932	4.33E-24	46149.822	150	307.665	4.589	2.61E-25	637.104	150	4.247	5.77	1.88E-32
	Error	0.016	298	0.000053			15421.709	230	67.051			169.318	230	0.736		
	Total	0.047	448				61571.531	380				806.422	380			
Co	Group	0.399	150	0.003	3.095	5.60E-17	10473.224	150	69.821	3.802	4.85E-20	0.907	150	0.006	3.03	1.82E-14
	Error	0.256	298	0.001			4223.919	230	18.365			0.459	230	0.002		
	Total	0.656	448				14697.143	380				1.366	380			
Cu	Group	2443.406	150	16.289	2.637	6.17E-13	134317.421	150	895.449	2.963	5.75E-14	1843.575	150	12.291	2.457	3.88E-10
	Error	1841.056	298	6.178			69509.017	230	302.213			1150.721	230	5.003		
	Total	4284.462	448				203826.437	380				2994.296	380			
Fe	Group	299.049	150	1.994	1.303	0.028	330209.856	150	2201.399	1.392	0.012	13100.626	150	87.338	1.997	1.00E-06
	Error	456.108	298	1.531			363738.077	230	1581.47			10059.193	230	43.736		
	Total	755.156	448				693947.933	380				23159.818	380			
K	Group	107867342.4	150	719115.6	1.676	9.00E-05	752653641.8	150	5017691	1.301	0.036	5142689325	150	34284595	1.183	0.126169
	Error	127839188.1	298	428990.6			886919699.8	230	3856173			6668136502	230	28991898		
	Total	235706530.5	448				1639573342	380				11810825826	380			
Mg	Group	16044405.59	150	106962.7	3.156	1.66E-17	33647792.88	150	224318.6	2.19	4.04E-08	55217989.75	150	368119.9	2.288	7.33E-09
	Error	10100191.96	298	33893.26			23556466.56	230	102419.4			36998765.16	230	160864.2		
	Total	26144597.55	448				57204259.44	380				92216754.91	380			
Mn	Group	14328.888	150	95.526	1.974	3.66E-07	4208.248	150	28.055	3.041	1.51E-14	247681.977	150	1651.213	3.313	1.49E-16
	Error	14422.388	298	48.397			2122.008	230	9.226			114639.278	230	498.432		
	Total	28751.277	448				6330.256	380				362321.255	380			
Mo	Group	108.24	150	0.722	2.655	4.28E-13	48315.036	150	322.1	1.381	0.014	18541.136	150	123.608	2.45	4.38E-10
	Error	81.007	298	0.272			53649.824	230	233.26			11605.658	230	50.459		
	Total	189.247	448				101964.86	380				30146.794	380			
Ni	Group	53.323	150	0.355	3.555	6.07E-21	203.065	150	1.354	1.205	0.102	3.657576284	150	0.024384	2.459	5.47E-10
	Error	29.795	298	0.1			258.379	230	1.123			2.201788826	230	0.009918		
	Total	83.118	448				461.444	380				5.85936511	380			
P	Group	74566795.33	150	497112	2.361	2.88E-10	76413047	150	509420.3	1.586	0.001	300554558.7	150	2003697	2.489	2.18E-10
	Error	59383832.35	298	210581			73895058.44	230	321282.9			185123386.1	230	804884.3		
	Total	133950627.7	448				150308105.4	380				485677944.8	380			
Rb	Group	171.519	150	1.143	2.8	2.20E-14	2342.141	150	15.614	2.941	8.43E-14	3670.783	150	24.472	2.239	1.74E-08
	Error	121.686	298	0.408			1221.212	230	5.31			2514.35	230	10.932		
	Total	293.206	448				3563.354	380				6185.132	380			
S	Group	3800629.457	150	25337.53	1.561	7.16E-04	513174741.7	150	3421165	1.581	0.001	189470482.1	150	1263137	2.302	5.75E-09
	Error	4576301.862	298	16228.02			497715250.1	230	2163979			126184040.3	230	548626.3		
	Total	8376931.319	448				1010889992	380				315654522.4	380			
Sr	Group	10.447	150	0.07	2.736	8.10E-14	283.5013393	150	1.890009	1.216509	0.097	89.954	150	0.6	1.528	0.002
	Error	7.585	298	0.025			315.3875648	230	1.553633			90.281	230	0.393		
	Total	18.032	448				598.8889041	380				180.235	380			

Element	Source of variation	11GGH[1]					15RTH[2]					15SHH[3]				
		SS	DF	MS	F	*p* -value	SS	DF	MS	F	*p* -value	SS	DF	MS	F	*p* -value
Zn	Group	22964.701	150	153.098	2.582	1.89E-12	178330.297	150	1188.869	1.253	0.062	24785.34	150	165.236	1.641	3.58E-04
	Error	17672.563	298	59.304			218170.172	230	948.566			23162.987	230	100.709		
	Total	40637.264	448				396500.47	380				47948.327	380			

[1]: Grains of RILs grown under semi-flooded condition in a greenhouse in 2011

[2]: Roots of RILs grown hydroponically in 2015

[3]: Shoots of RILs grown hydroponically in 2015

Supplementary Table 6. The concentrations of 16 elements in the grains, roots and shoots of two parental lines and LT-RIL population. The units for all elements are μg g-1 dry weight.

Element	LM			TQ			LR-RIL population						Skewness			Kurtosis			Shapiro-Wilk test		
	11GGH[1]	15RTH[2]	15SHH[3]	11GGH	15RTH	15SHH	11GGH		15RTH		15SHH		11GGH	15RTH	15SHH	11GGH	15RTH	15SHH	*p* -value		
	mean ± SD	mean ± SD	mean ± SD	mean ± SD	mean ± SD	mean ± SD	mean ± SD	Range	mean ± SD	Range	mean ± SD	Range							11GGH	15RTH	15SHH
As	0.037 ± 0.0056	2.97 ± 0.5	1.61 ± 0.19	0.032 ± 0.0045	4.11 ± 0.41	2.11 ± 0.21	0.033 ± 0.0072	0.015 ~ 0.056	3.65 ± 0.80	2.23 ~ 6.80	1.78 ± 0.26	1.16 ~ 2.50	0.0051	1.08	0.14	0.23	1.31	0.0036	0.72	8.40E-08	0.57
Ca	155 ± 20	508 ± 61	3300 ± 542	161 ± 24	481 ± 49	3597 ± 592	149 ± 29	75 ~ 272	643 ± 211	387 ~ 2014	3459 ± 379	2137 ~ 4801	0.47	2.8	-0.054	1.98	11.53	0.88	0.0029	1.4E-16	0.44
Cd	0.018 ± 0.0064	30.46 ± 5.69	2.23 ± 0.68	0.02 ± 0.0036	31.62 ± 3.68	3.06 ± 1.07	0.020 ± 0.0084	0.0087 ~ 0.054	32.07 ± 10.78	9.48 ~ 83.55	2.71 ± 1.35	0.69 ~ 8.35	1.55	0.91	1.38	3.21	2.3	2.71	5.1E-10	3.80E-05	1.5E-09
Co	0.073 ± 0.017	16.96 ± 3.22	0.2 ± 0.032	0.067 ± 0.019	17.88 ± 6.33	0.19 ± 0.043	0.074 ± 0.030	0.0030 ~ 0.26	16.52 ± 5.09	5.79 ~ 40.93	0.20 ± 0.054	0.12 ~ 0.43	1.85	0.94	1.32	9.2	2.76	2.95	3.1E-09	1.20E-05	9.4E-09
Cu	11.08 ± 2.13	77.4 ± 11.26	11.57 ± 1.54	10.87 ± 1.83	59.65 ± 6.26	12.95 ± 1.21	11.26 ± 2.32	6.58 ~ 18.52	71.25 ± 18.28	37.26 ~ 145.52	12.23 ± 2.29	7.65 ~ 19.96	0.49	1.22	0.84	0.13	2.08	0.84	0.04	1.80E-08	1.00E-05
Fe	5.54 ± 0.43	143.24 ± 16.65	61.32 ± 4.28	5.23 ± 0.31	112.57 ± 28.23	68.5 ± 4.16	5.48 ± 0.83	2.76 ~ 8.01	130.72 ± 31.72	78.97 ~ 307.87	69.34 ± 7.97	52.05 ~ 114.34	-0.27	2.44	2.69	0.94	8.98	12.31	0.094	9.9E-15	3E-15
K	4409 ± 424	4199 ± 1731	15387 ± 3708	4296 ± 250	8378 ± 2857	15046 ± 947	4372 ± 493	3330 ~ 6113	5721 ± 1452	2289 ~ 10524	15057 ± 4301	7004 ~ 34351	0.76	0.19	0.82	0.67	-0.0016	1.86	4.50E-04	0.61	4.50E-05
Mg	1846 ± 93	1443 ± 440	6092 ± 432	1785 ± 93	1454 ± 153	5776 ± 255	1813 ± 191	1455 ~ 2747	1513 ± 319	797 ~ 2730	5801 ± 410	4677 ~ 6871	1	0.6	0.3	3.39	0.93	0.27	2.00E-05	0.0012	0.082
Mn	16.03 ± 5.97	8.57 ± 1.53	90.32 ± 33.28	14.51 ± 2.27	12.63 ± 1.77	115.22 ± 27.58	14.20 ± 5.63	5.88 ~ 35.46	11.95 ± 4.12	6.23 ~ 44.48	94.50 ± 26.082	36.39 ~ 221.58	1.15	3.01	0.69	1.5	20.02	2.61	9.40E-08	1.6E-14	1.10E-04
Mo	2.62 ± 0.45	45.54 ± 7.56	30.34 ± 5.76	2 ± 0.2	41.67 ± 4.27	27.2 ± 2.98	2.09 ± 0.49	0.95 ~ 3.71	46.31 ± 13.99	20.80 ~ 146.64	29.52 ± 8.14	14.60 ~ 73.44	0.67	2.73	1.71	1.11	14.44	6.02	0.0014	2.5E-14	8.9E-11
Ni	0.85 ± 0.18	2.94 ± 0.38	0.71 ± 0.071	1.07 ± 0.46	2.64 ± 0.75	0.74 ± 0.12	0.92 ± 0.34	0.36 ~ 2.74	2.76 ± 0.67	1.57 ~ 8.95	0.79 ± 0.29	0.54 ~ 3.88	1.74	4.52	7.26	5.77	38.11	69.3	1.7E-09	6.1E-18	1.2E-23
P	4706 ± 325	2964 ± 497	8597 ± 486	4673 ± 250	2167 ± 192	6585 ± 370	4567 ± 581	2595 ~ 5826	2625 ± 586	440 ~ 6756	7333 ± 936	5579 ~ 10453	-1.21	2.31	0.76	2.03	14.24	0.33	7.40E-08	5.9E-14	3.10E-05
Rb	3.87 ± 0.6	15.92 ± 0.98	14.64 ± 2.09	3.63 ± 0.39	17.95 ± 1.35	15.8 ± 1.4	3.58 ± 0.65	0.74 ~ 5.61	16.83 ± 2.64	11.38 ~ 28.04	16.77 ± 3.44	8.27 ~ 41.12	-0.19	1.06	2.27	2.57	2.21	13.36	4.20E-04	9.40E-07	5.5E-12
S	1135 ± 79	4433 ± 710	6058 ± 340	1181 ± 62	4553 ± 292	5645 ± 332	1129 ± 148	442 ~ 1385	5001 ± 1555	3200 ~ 14563	6036 ± 716	4658 ~ 8056	-1.6	3.45	0.29	3.66	15.87	-0.44	4.6E-10	6.3E-19	0.041
Sr	0.68 ± 0.12	3.12 ± 1.52	4 ± 0.86	0.75 ± 0.11	2.25 ± 0.38	3.99 ± 1.01	0.66 ± 0.15	0.23 ~ 1.20	3.62 ± 2.74	1.25 ~ 24.77	4.35 ± 0.52	2.98 ~ 5.93	0.22	4.08	0.19	0.75	23.01	0.35	0.43	1.4E-20	0.64
Zn	39.1 ± 2.42	53.85 ± 3.41	33.65 ± 7.8	36.47 ± 3.21	49.72 ± 5.42	49.45 ± 8.63	37.83 ± 7.35	24.21 ~ 66.94	63.97 ± 21.59	35.70 ~ 217.18	43.33 ± 8.78	25.73 ~ 69.49	1.33	2.76	0.57	2.68	13.85	0.24	4.10E-08	4.4E-15	0.0012

[1]: Grains of RILs grown under semi-flooded condition in a greenhouse in 2011

[2]: Roots of RILs grown hydroponically in 2015

[3]: Shoots of RILs grown hydroponically in 2015

Supplementary Table 7. QTLs controlling the elemental concentrations in grains, shoots or roots of LT-RILs grown in different environments. The QTLs in blue or orange background have the same QTL mapping interval.

Element	Environment[1]	Tissue	Chromosome	Start (cM)	End (cM)	Physical position (Kb)	LOD	ADD[2]	PVE[3]
As	08GF2	Grain	2	51.838	54.839	4987 ~ 5798	3.098	0.059	5.02
As	15HSH	Shoot	3	0	0.783	82 ~ 858	6.542	-0.1	13.259
As	02GF	Grain	3	0.783	1.175	731 ~ 1013	3.507	-0.03	5.96
As	07GF	Grain	3	1.965	2.161	757 ~ 1496	6.531	0.054	6.791
As	07GF	Grain	3	3.539	3.734	1720 ~ 2071	5.883	0.053	6.661
As	15HRT	Root	3	3.539	3.734	1720 ~ 2071	3.548	-0.24	9.173
As	06GF	Grain	3	5.904	6.49	2150 ~ 2964	3.597	-0.066	8.765
As	03GF	Grain	3	136.011	136.322	27110 ~ 27985	5.256	0.029	7.462
As	02-06_mean	Grain	3	154.893	155.205	30365 ~ 30750	3.341	0.012	1.942
As	F_mean	Grain	3	160.317	160.707	32945 ~ 33220	3.468	0.029	3.403
As	06GF	Grain	5	73.667	73.667	17432 ~ 17675	4.813	-0.054	5.656
As	07GU	Grain	5	79.827	79.827	19868 ~ 20262	4.022	-0.006	6.874
As	02-06_mean	Grain	8	1.793	1.988	40 ~ 737	3.558	-0.019	4.778
As	03GF	Grain	9	83.078	83.39	20581 ~ 20921	5.194	-0.029	6.572
As	02-06_mean	Grain	9	83.078	83.39	20581 ~ 20921	3.903	-0.02	5.386
Ca	06GF	Grain	2	106.137	106.529	21244 ~ 21828	4.896	5.005	6.411
Ca	02-06_mean	Grain	2	109.099	109.294	22418 ~ 22919	6.957	4.44	5.632
Ca	U_mean	Grain	2	114.022	114.411	23325 ~ 23873	9.323	7.02	9.651
Ca	08GU1	Grain	3	0	0.39	82 ~ 751	3.749	3.307	2.095
Ca	08GU1	Grain	3	1.175	2.161	698 ~ 1496	3.4	3.16	1.913
Ca	07GF	Grain	3	72.572	73.276	16795 ~ 17263	3.623	-6.301	4.899
Ca	08GF2	Grain	3	72.572	73.276	16795 ~ 17263	7.33	-6.734	13.97
Ca	03GF	Grain	3	72.964	73.276	16895 ~ 17416	9.422	-7.272	11.356
Ca	F_mean	Grain	3	72.964	73.276	16895 ~ 17416	5.71	-5.477	8.328
Ca	02GF	Grain	3	73.785	74.097	17130 ~ 17534	6.552	-7.581	11.008
Ca	15HSH	Shoot	7	33.839	34.035	4014 ~ 4330	3.863	-98.295	6.458
Ca	08GU3	Grain	10	105.16	106.755	19532 ~ 19828	21.817	-9.481	2.942
Ca	08GU1	Grain	12	113.623	113.818	25482 ~ 26203	4.352	-5.821	6.49
Cd	U_mean	Grain	2	57.03	57.42	5672 ~ 5923	3.192	0.001	2.936
Cd	07GU	Grain	2	64.764	64.764	8194 ~ 8419	5.27	0.001	0.118
Cd	02GF	Grain	2	138.302	138.497	28585 ~ 29657	3.627	-0.002	6.212
Cd	F_mean	Grain	2	138.302	138.497	28585 ~ 29657	5.122	-0.002	6.294
Cd	08GU3	Grain	3	5.904	6.49	2150 ~ 2964	4.244	-0.035	11.191

Element	Environment[1]	Tissue	Chromosome	Start (cM)	End (cM)	Physical position (Kb)	LOD	ADD[2]	PVE[3]
Cd	08GF2	Grain	3	29.1	29.493	8102 ~ 8634	5.241	-0.001	2.775
Cd	11GG	Grain	3	136.011	136.322	27110 ~ 27985	3.951	-0.003	10.181
Cd	03GF	Grain	3	146.673	146.984	29007 ~ 29488	4.538	-0.006	6.013
Cd	F_mean	Grain	5	76.703	76.703	16965 ~ 17194	4.702	0.002	2.933
Cd	02GF	Grain	6	110.491	110.88	13728 ~ 16296	3.768	-0.003	6.861
Cd	02-06_mean	Grain	6	122.746	122.942	20927 ~ 21271	3.829	-0.005	6.072
Cd	U_mean	Grain	10	93.641	93.952	17262 ~ 17851	4.28	-0.001	8.102
Cd	15HRT	Root	11	83.79	84.18	19608 ~ 19944	6.287	3.972	12.73
Cd	15HSH	Shoot	11	83.79	84.18	19608 ~ 19944	4.294	0.513	11.294
Co	08GU1	Grain	1	34.052	34.247	4924 ~ 5443	3.331	-0.012	7.298
Co	U_mean	Grain	1	34.052	34.247	4924 ~ 5443	7.485	-0.005	12.536
Co	02-06_mean	Grain	1	34.052	34.247	4924 ~ 5443	3.704	-0.004	5.958
Co	08GF2	Grain	1	35.628	36.212	4411 ~ 5699	5.674	-0.004	11.776
Co	03GF	Grain	1	40.72	40.72	7268 ~ 7497	4.303	-0.004	4.71
Co	11GG	Grain	1	48.182	48.572	8813 ~ 9111	5.006	-0.008	6.734
Co	02GF	Grain	2	74.093	74.288	10459 ~ 10672	4.364	0.004	7.993
Co	F_mean	Grain	3	101.223	102.634	23148 ~ 24369	3.698	0.002	1.793
Co	F_mean	Grain	3	126.517	131.43	26439 ~ 27472	3.859	0.002	2.145
Co	08GU3	Grain	3	177.284	179.309	35422 ~ 35967	4.54	-0.002	0.162
Co	08GF2	Grain	5	4.214	4.214	1927 ~ 2149	4.716	-0.003	9.274
Co	11GG	Grain	6	77.648	77.842	6891 ~ 7095	3.404	0.007	5.618
Co	07GF	Grain	6	120.343	120.736	20102 ~ 20456	6.262	-0.007	14.201
Co	F_mean	Grain	6	120.343	120.736	20102 ~ 20456	4.161	-0.004	8.527
Co	02-06_mean	Grain	6	120.343	120.736	20102 ~ 20456	4.666	-0.004	7.727
Cu	07GF	Grain	2	51.838	54.839	4987 ~ 5798	9.111	-0.206	10.123
Cu	08GF2	Grain	2	51.838	54.839	4987 ~ 5798	9.043	-0.313	15.664
Cu	08GU1	Grain	2	51.838	54.839	4987 ~ 5798	7.16	-0.435	12.137
Cu	07GU	Grain	2	51.838	54.839	4987 ~ 5798	14.342	-0.807	26.35
Cu	F_mean	Grain	2	51.838	54.839	4987 ~ 5798	10.043	-0.203	13.364
Cu	U_mean	Grain	2	51.838	54.839	4987 ~ 5798	13.879	-0.252	15.698
Cu	02-06_mean	Grain	2	51.838	54.839	4987 ~ 5798	14.398	-0.318	20.196
Cu	02GF	Grain	3	107.901	108.096	25061 ~ 25359	3.566	0.147	4.98
Cu	15HSH	Shoot	3	139.244	143.351	28517 ~ 28965	3.758	0.477	4.009
Cu	15HSH	Shoot	3	145.266	149.845	28932 ~ 30643	4.188	0.462	3.757
Cu	08GU3	Grain	4	62.146	62.457	20459 ~ 20886	5.427	0.554	11.801

Element	Environment[1]	Tissue	Chromosome	Start (cM)	End (cM)	Physical position (Kb)	LOD	ADD[2]	PVE[3]
Cu	03GF	Grain	8	38.724	39.508	3914 ~ 4481	6.565	-0.206	9.481
Cu	02GF	Grain	8	39.116	39.508	4110 ~ 4481	3.505	-0.171	6.761
Fe	03GF	Grain	2	63.981	64.176	7426 ~ 8010	3.518	0.379	5.276
Fe	F_mean	Grain	2	65.156	65.351	8184 ~ 8521	4.752	0.432	10.775
Fe	08GF2	Grain	2	70.542	71.129	8983 ~ 9931	6.221	0.478	11.919
Fe	U_mean	Grain	2	70.542	71.129	8983 ~ 9931	4.054	0.583	7.404
Fe	08GF2	Grain	2	74.093	74.288	10459 ~ 10672	4.369	0.397	8.028
Fe	02-06_mean	Grain	2	80.016	80.992	16363 ~ 16704	24.327	0.713	2.15
Fe	U_mean	Grain	3	0	0.39	82 ~ 751	11.469	0.536	6.243
Fe	F_mean	Grain	3	0.195	0.39	10 ~ 751	5.243	0.342	6.744
Fe	07GF	Grain	3	1.965	3.147	757 ~ 1733	9.87	0.152	0.131
Fe	07GU	Grain	3	1.965	2.161	757 ~ 1496	7.916	0.746	21.529
Fe	15HSH	Shoot	3	56.866	57.061	13858 ~ 14577	4.146	-1.48	3.35
Fe	11GG	Grain	4	22.085	22.085	7803 ~ 8065	4.331	0.219	6.972
K	08GF2	Grain	1	35.433	35.628	4441 ~ 5565	5.156	-129.614	13.164
K	F_mean	Grain	1	35.433	35.628	4441 ~ 5565	5.072	-88.211	13.89
K	U_mean	Grain	1	35.433	35.628	4441 ~ 5565	4.481	-107.642	14.757
K	02-06_mean	Grain	1	35.433	35.628	4441 ~ 5565	6.634	-92.566	13.722
K	06GF	Grain	1	40.72	40.72	7268 ~ 7497	3.743	-102.376	14.403
K	07GF	Grain	1	43.751	44.063	6487 ~ 7945	6.75	-95.89	14.003
K	08GF2	Grain	1	43.751	43.751	6487 ~ 6735	3.196	-106.079	8.823
K	02GF	Grain	1	63.879	64.191	12322 ~ 12743	4.945	-74.328	8.619
K	11GG	Grain	1	119.806	120.196	27893 ~ 28357	3.925	-138.792	7.972
K	03GF	Grain	1	123.474	123.866	28388 ~ 29992	3.832	-79.53	10.886
K	06GF	Grain	1	129.77	129.77	30534 ~ 30758	3.238	-92.426	11.739
K	07GU	Grain	3	51.738	51.933	11873 ~ 12572	4.167	98.753	5.327
K	F_mean	Grain	3	171.565	172.348	34016 ~ 34411	5.316	-82.443	12.132
Mg	08GU3	Grain	2	79.626	80.016	15571 ~ 16509	7.604	-20.056	1.448
Mg	07GU	Grain	2	89.172	89.561	18039 ~ 18477	3.195	-39.702	8.57
Mg	08GF2	Grain	3	173.525	173.915	34655 ~ 35388	5.75	-47.435	11.807
Mg	U_mean	Grain	3	173.525	173.915	34655 ~ 35388	7.846	-50.146	16.643
Mg	06GF	Grain	4	219.996	219.996	32593 ~ 32825	3.31	-39.786	9.881
Mg	08GU1	Grain	5	2.644	3.036	1461 ~ 2660	3.162	21.27	2.326
Mg	02-06_mean	Grain	7	53.847	54.159	8265 ~ 8937	4.107	-35.119	10.927
Mg	06GF	Grain	8	35.397	35.591	3396 ~ 3608	3.071	30.251	5.713

Element	Environment[1]	Tissue	Chromosome	Start (cM)	End (cM)	Physical position (Kb)	LOD	ADD[2]	PVE[3]
Mg	07GF	Grain	10	87.752	87.752	13975 ~ 14209	4.207	31.029	5.895
Mg	03GF	Grain	10	109.525	109.525	20819 ~ 21071	4.173	31.05	6.013
Mg	06GF	Grain	11	4.262	4.457	1104 ~ 1362	4.869	-43.989	11.002
Mg	02GF	Grain	11	12.889	13.084	2667 ~ 2976	4.77	-37.921	8.223
Mg	02GF	Grain	11	17.871	18.263	3588 ~ 3894	4.303	-33.591	6.578
Mg	F_mean	Grain	11	19.458	19.458	3833 ~ 4055	4.073	-23.985	5.465
Mg	03GF	Grain	12	117.197	117.197	25394 ~ 25616	5.28	-37.56	9.801
Mg	07GU	Grain	12	121.465	121.857	25622 ~ 26855	4.421	-42.14	9.655
Mn	08GF2	Grain	2	130.814	131.126	26561 ~ 26832	3.426	1.963	11.489
Mn	15HRT	Root	3	24.332	24.723	6477 ~ 6963	10.949	-2.113	28.198
Mn	07GF	Grain	3	172.939	173.329	34394 ~ 34776	3.701	1.75	9.517
Mn	02-06_mean	Grain	5	78.298	78.298	17682 ~ 17908	4.113	1.695	9.792
Mn	08GU1	Grain	7	51.187	51.382	7007 ~ 7904	3.122	2.075	8.407
Mn	06GF	Grain	7	52.367	52.756	8331 ~ 9044	5.38	2.715	13.594
Mn	07GF	Grain	7	56.316	56.628	9001 ~ 10330	3.589	1.729	9.29
Mn	F_mean	Grain	7	56.316	56.628	9001 ~ 10330	6.881	1.973	14.06
Mn	U_mean	Grain	7	83.113	83.308	14625 ~ 14955	4.717	2.246	15.333
Mn	03GF	Grain	11	14.279	14.279	2796 ~ 3031	4.434	-1.848	5.222
Mn	15HSH	Shoot	11	83.79	84.18	19608 ~ 19944	6.924	10.941	14.777
Mo	08GU3	Grain	3	3.734	5.314	1690 ~ 2515	9.982	-0.071	2.837
Mo	03GF	Grain	3	154.893	155.205	30365 ~ 30750	6.475	0.028	10.782
Mo	F_mean	Grain	3	154.893	155.205	30365 ~ 30750	7.709	0.025	11.015
Mo	03GF	Grain	3	157.123	157.435	30456 ~ 30845	5.089	0.026	8.534
Mo	06GF	Grain	3	167.019	167.214	32479 ~ 33122	7.115	0.048	19.514
Mo	06GF	Grain	3	171.956	172.348	33992 ~ 34411	7.233	0.046	17.3
Mo	07GU	Grain	5	16.782	17.094	4908 ~ 5244	3.795	-0.016	5.603
Mo	08GF2	Grain	7	90.678	90.678	16645 ~ 16866	6.578	0.037	16.518
Mo	07GU	Grain	7	90.678	90.678	16645 ~ 16866	3.541	0.021	9.974
Mo	F_mean	Grain	7	90.678	90.678	16645 ~ 16866	8.35	0.027	12.89
Mo	U_mean	Grain	7	90.678	90.678	16645 ~ 16866	9.998	0.034	14.77
Mo	02-06_mean	Grain	7	90.678	90.678	16645 ~ 16866	3.466	0.028	7.952
Mo	08GF2	Grain	7	92.078	92.47	16902 ~ 17246	7.946	0.038	17.239
Mo	02-06_mean	Grain	8	1.793	1.988	40 ~ 737	3.494	0.026	6.809
Mo	15HSH	Shoot	8	50.006	50.707	6478 ~ 7234	3.822	2.115	7.09
Mo	08GU3	Grain	12	122.849	125.047	26689 ~ 27152	11.267	0.058	1.901

Element	Environment[1]	Tissue	Chromosome	Start (cM)	End (cM)	Physical position (Kb)	LOD	ADD[2]	PVE[3]
Ni	15HRT	Root	1	211.996	212.385	42038 ~ 42512	4.024	0.2	5.643
Ni	07GU	Grain	3	175.701	176.693	35367 ~ 35720	3.796	-0.542	10.384
Ni	11GG	Grain	4	219.8	220.191	33503 ~ 34084	3.788	-0.074	4.653
Ni	08GF2	Grain	5	0	0.312	26 ~ 183	3.252	-0.1	3.256
Ni	08GF2	Grain	6	124.137	124.449	21243 ~ 21675	5.546	-0.127	5.404
Ni	F_mean	Grain	6	124.137	124.449	21243 ~ 21675	5.615	-0.039	4.931
Ni	15HSH	Shoot	11	5.652	6.437	1343 ~ 2237	3.435	0.046	2.889
P	03GF	Grain	1	28.704	29.094	4151 ~ 4469	6.099	-77.026	7.744
P	U_mean	Grain	1	35.628	36.212	4411 ~ 5699	3.854	-63.955	6.324
P	08GF2	Grain	1	123.474	123.866	28388 ~ 29992	4.728	-73.893	6.734
P	08GF2	Grain	1	125.846	125.846	28836 ~ 29068	3.18	-44.273	2.418
P	08GU1	Grain	2	92.161	92.473	18706 ~ 19096	3.57	-102.2	7.777
P	08GF2	Grain	3	51.738	52.129	11873 ~ 12720	3.517	42.074	2.163
P	F_mean	Grain	3	65.794	66.185	15186 ~ 15814	4.119	36.271	2.658
P	F_mean	Grain	3	179.309	179.309	35715 ~ 35967	4.191	-57.769	6.742
P	U_mean	Grain	3	179.309	179.309	35715 ~ 35967	3.944	-73.251	8.296
P	07GF	Grain	3	179.9	179.9	35596 ~ 35836	5.533	-82.364	7.573
P	08GU3	Grain	5	76.703	76.898	16965 ~ 17377	5.366	-119.264	1.712
P	15HRT	Root	6	17.423	17.617	1748 ~ 2031	5.646	218.824	13.964
P	02GF	Grain	7	26.183	26.495	2745 ~ 3219	3.099	76.841	6.5
P	06GF	Grain	7	26.495	27.402	2934 ~ 3285	3.031	59.629	3.698
P	06GF	Grain	8	35.397	35.591	3396 ~ 3608	3.153	78.171	7.066
P	11GG	Grain	9	21.795	21.991	8638 ~ 9061	3.623	150.184	6.123
P	07GU	Grain	12	122.849	122.849	26951 ~ 27194	3.674	-90.206	7.023
Rb	15HRT	Root	1	67.711	67.711	12414 ~ 12650	4.142	-0.821	9.499
Rb	07GF	Grain	3	0	0.39	82 ~ 751	5.255	-0.824	11.025
Rb	02GF	Grain	3	0.783	1.175	731 ~ 1013	9.515	-1.21	14.981
Rb	08GU1	Grain	3	0.783	1.175	731 ~ 1013	8.467	-2.501	22.84
Rb	02-06_mean	Grain	3	1.965	2.161	757 ~ 1496	13.717	-0.975	21.945
Rb	08GF2	Grain	3	2.951	3.147	1477 ~ 1733	3.034	-0.879	10.2
Rb	11GG	Grain	3	5.118	5.314	2211 ~ 2515	4.61	-0.175	7.448
Rb	06GF	Grain	3	5.904	6.49	2150 ~ 2964	6.993	-2.304	19.17
Rb	F_mean	Grain	3	5.904	6.49	2150 ~ 2964	7.833	-0.89	15.705
Rb	06GF	Grain	3	6.1	6.1	2335 ~ 2568	5.257	-2.346	18.344
Rb	03GF	Grain	9	95.087	95.283	22149 ~ 22591	6.295	-0.731	12.116

Element	Environment[1]	Tissue	Chromosome	Start (cM)	End (cM)	Physical position (Kb)	LOD	ADD[2]	PVE[3]
S	08GU3	Grain	2	78.919	80.211	15471 ~ 16319	10.803	-101.379	3.193
S	08GF2	Grain	2	139.088	139.088	29516 ~ 29758	4.508	-64.371	9.87
S	07GF	Grain	5	1.019	1.331	2258 ~ 2547	6.88	-47.237	13.247
S	08GF2	Grain	5	1.019	1.331	2258 ~ 2547	6.688	-74.081	12.947
S	U_mean	Grain	5	1.019	1.331	2258 ~ 2547	8.145	-62.772	16.505
S	F_mean	Grain	5	2.644	3.231	1461 ~ 2759	8.008	-50.696	12.698
S	08GU1	Grain	5	9.577	9.577	2797 ~ 3089	4.059	-66.13	11.626
S	U_mean	Grain	8	49.811	50.006	6403 ~ 6794	5.393	-47.078	9.283
S	03GF	Grain	10	118.967	118.967	21215 ~ 21439	3.18	-49.514	5.201
S	15HSH	Shoot	12	113.623	113.818	25482 ~ 26203	5.326	162.836	5.182
Sr	07GF	Grain	2	119.339	120.04	24308 ~ 24723	7.951	0.041	9.976
Sr	03GF	Grain	3	65.599	65.989	15009 ~ 15680	22.666	-0.11	30.047
Sr	08GU1	Grain	3	65.599	65.989	15009 ~ 15680	4.839	-0.057	18.953
Sr	02-06_mean	Grain	3	65.599	65.989	15009 ~ 15680	15.376	-0.055	26.091
Sr	02GF	Grain	3	65.794	66.185	15186 ~ 15814	14.432	-0.074	19.508
Sr	06GF	Grain	3	67.38	67.575	15630 ~ 16243	9.622	-0.081	28.274
Sr	07GF	Grain	3	67.38	67.575	15630 ~ 16243	4.453	-0.034	6.984
Sr	F_mean	Grain	3	67.38	67.575	15630 ~ 16243	22.104	-0.064	23.994
Sr	U_mean	Grain	3	67.38	67.575	15630 ~ 16243	5.578	-0.041	11.535
Sr	08GF2	Grain	3	72.572	72.964	16795 ~ 17302	8.803	-0.053	15.659
Sr	11GG	Grain	3	171.37	171.761	33876 ~ 34377	3.523	0.051	10.246
Sr	08GU3	Grain	4	62.146	62.457	20459 ~ 20886	4.281	-0.012	4.297
Sr	15HSH	Shoot	11	83.595	83.79	19351 ~ 19879	4.03	-0.129	6.032
Sr	15HRT	Root	11	89.46	89.655	20598 ~ 20934	3.009	-0.613	4.977
Zn	02GF	Grain	2	85.093	85.288	17512 ~ 17936	4.797	1.14	11.163
Zn	U_mean	Grain	2	100.645	101.428	20098 ~ 20585	5.015	0.972	12.163
Zn	07GF	Grain	2	101.037	101.233	20274 ~ 21435	4.051	1.086	10.23
Zn	08GF2	Grain	2	101.037	101.233	20274 ~ 21435	5.918	0.953	10.67
Zn	07GF	Grain	3	25.115	25.31	6921 ~ 7503	5.337	1.15	11.771
Zn	F_mean	Grain	3	66.185	70.965	15310 ~ 16650	3.054	-0.139	0.308
Zn	08GF2	Grain	4	222.842	222.842	33160 ~ 33413	3.744	0.813	7.264
Zn	02-06_mean	Grain	5	100.027	100.027	23019 ~ 23246	3.239	0.749	9.558
Zn	15HSH	Shoot	11	83.595	83.79	19351 ~ 19879	4.62	2.861	10.328

Element	Environment[1]	Tissue	Chromosome	Start (cM)	End (cM)	Physical position (Kb)	LOD	ADD[2]	PVE[3]

[1]: 02GF: grains (G) of RILs grown under flooded (F) on 2002;
03GF: grains (G) of RILs grown under flooded (F) on 2003;
06GF: grains (G) of RILs grown under flooded (F) on 2006;
07GF: grains (G) of RILs grown under flooded (F) on 2007;
07GU: grains (G) of RILs grown under unflooded (U) on 2007;
08GF2: grains (G) of RILs grown under flooded (F) in the field site number 2 on 2008;
08GU1: grains (G) of RILs grown under unflooded (U) in the field site number 1 on 2008;
08GU3: grains (G) of RILs grown under unflooded (U) in the field site number 3 on 2008;
11GGH: grains (G) of RILs grown in a greenhouse (GH) condition on 2011;
15RTH: roots (RT) of RILs grown hydroponically (H) on 2015;
15SHH: shoots (SH) of RILs grown hydroponically (H) on 2015;
02-06_mean: the LS mean of 02GF, 03GF and 06GF;
U_mean: the LS means of 07GU, 08GU1 and 08GU3;
F_mean: the LS means of 02GF, 03GF, 06GF, 07GF and 08GF2.

[2]ADD: additive effect

[3]PVE: percentage of variance explained

Supplementary Table 8. The variance of the ionome explained by each principle components.

Traits[1]		PC1	PC2	PC3	PC4	PC5	PC6	PC7	PC8	PC9	PC10	PC11	PC12	PC13	PC14	PC15	PC16
02GF	Eigenvalue	0.21674	0.17461	0.11971	0.09224	0.07682	0.0701	0.05999	0.04042	0.03635	0.02749	0.02247	0.02021	0.01611	0.01228	0.00787	0.0066
	Cumulative proportion	0.21674	0.39135	0.51107	0.60331	0.68012	0.75022	0.81021	0.85063	0.88698	0.91446	0.93693	0.95714	0.97325	0.98553	0.9934	1
03GF	Eigenvalue	0.20448	0.17675	0.13223	0.10087	0.07378	0.06293	0.04801	0.04357	0.03059	0.02624	0.02559	0.02198	0.0209	0.01714	0.00945	0.00548
	Cumulative proportion	0.20448	0.38123	0.51346	0.61433	0.68811	0.75105	0.79906	0.84263	0.87322	0.89946	0.92505	0.94703	0.96793	0.98507	0.99452	1
06GF	Eigenvalue	0.22557	0.20026	0.12874	0.09443	0.06101	0.05189	0.046	0.04008	0.03013	0.02837	0.02294	0.02091	0.01573	0.01395	0.01273	0.00725
	Cumulative proportion	0.22557	0.42583	0.55458	0.649	0.71001	0.7619	0.8079	0.84798	0.87812	0.90649	0.92943	0.95034	0.96607	0.98002	0.99275	1
07GF	Eigenvalue	0.19934	0.17739	0.11588	0.101	0.078	0.0605	0.05068	0.04789	0.03599	0.034	0.02986	0.02096	0.01925	0.01489	0.00804	0.00632
	Cumulative proportion	0.19934	0.37674	0.49262	0.59362	0.67162	0.73213	0.78281	0.8307	0.86668	0.90068	0.93054	0.9515	0.97075	0.98565	0.99368	1
07GU	Eigenvalue	0.22087	0.15175	0.11997	0.08871	0.07596	0.05624	0.05248	0.05122	0.04535	0.03425	0.02908	0.0213	0.0195	0.01634	0.01039	0.0066
	Cumulative proportion	0.22087	0.37262	0.49259	0.5813	0.65726	0.7135	0.76597	0.81719	0.86254	0.8968	0.92587	0.94717	0.96667	0.98301	0.9934	1
08GF2	Eigenvalue	0.2194	0.13885	0.12763	0.09405	0.07689	0.06178	0.06019	0.05048	0.03785	0.03438	0.03094	0.02124	0.01755	0.01505	0.00787	0.00588
	Cumulative proportion	0.2194	0.35825	0.48587	0.57992	0.65682	0.71859	0.77878	0.82926	0.86711	0.90149	0.93242	0.95366	0.97121	0.98626	0.99412	1
08GU1	Eigenvalue	0.2058	0.16129	0.10534	0.09194	0.07789	0.06333	0.0554	0.04757	0.04319	0.03663	0.02897	0.02649	0.02226	0.01625	0.0113	0.00637
	Cumulative proportion	0.2058	0.36709	0.47243	0.56437	0.64226	0.70559	0.76099	0.80856	0.85175	0.88837	0.91734	0.94383	0.96609	0.98233	0.99363	1
08GU3	Eigenvalue	0.19961	0.17995	0.11914	0.09822	0.07893	0.07406	0.05335	0.04743	0.04004	0.02588	0.02174	0.02005	0.01521	0.01149	0.0097	0.0052
	Cumulative proportion	0.19961	0.37956	0.4987	0.59692	0.67585	0.74991	0.80327	0.8507	0.89074	0.91661	0.93835	0.9584	0.97361	0.9851	0.9948	1
11GGH	Eigenvalue	0.1954	0.14156	0.10303	0.09363	0.08101	0.07484	0.06014	0.04687	0.04294	0.03543	0.03015	0.0273	0.02382	0.01845	0.01697	0.00847
	Cumulative proportion	0.1954	0.33696	0.43999	0.53362	0.61463	0.68946	0.74961	0.79648	0.83941	0.87485	0.905	0.9323	0.95612	0.97456	0.99153	1
15RTH	Eigenvalue	0.27083	0.20387	0.09078	0.07409	0.06272	0.05735	0.04672	0.04338	0.03525	0.0338	0.02746	0.01867	0.01528	0.01404	0.00383	0.00191
	Cumulative proportion	0.27083	0.47471	0.56549	0.63958	0.7023	0.75966	0.80638	0.84976	0.88501	0.91881	0.94627	0.96494	0.98022	0.99426	0.99809	1
15SHH	Eigenvalue	0.24598	0.15553	0.09938	0.08351	0.07253	0.06036	0.05111	0.04651	0.04156	0.03815	0.02795	0.02489	0.02049	0.01776	0.00877	0.00552
	Cumulative proportion	0.24598	0.40151	0.50089	0.5844	0.65693	0.71729	0.7684	0.81491	0.85647	0.89462	0.92257	0.94746	0.96795	0.98571	0.99448	1
F	Eigenvalue	0.23393	0.13784	0.11321	0.11114	0.07768	0.06325	0.05519	0.04426	0.03304	0.03132	0.02948	0.01879	0.01627	0.01495	0.01313	0.00654
	Cumulative proportion	0.23393	0.37177	0.48498	0.59612	0.6738	0.73704	0.79223	0.83649	0.86953	0.90085	0.93033	0.94912	0.96538	0.98033	0.99346	1
U	Eigenvalue	0.24216	0.13121	0.1065	0.09096	0.07953	0.06875	0.05685	0.049	0.04311	0.03211	0.02629	0.02221	0.01876	0.01448	0.01195	0.00613
	Cumulative proportion	0.24216	0.37336	0.47986	0.57082	0.65035	0.7191	0.77595	0.82495	0.86806	0.90017	0.92646	0.94867	0.96743	0.98191	0.99387	1
FUGG	Eigenvalue	0.32683	0.20463	0.08399	0.0724	0.05496	0.0507	0.04539	0.03519	0.02703	0.02226	0.01969	0.01604	0.01327	0.01068	0.00886	0.00811
	Cumulative proportion	0.32683	0.53145	0.61544	0.68784	0.74279	0.79349	0.83888	0.87407	0.9011	0.92336	0.94305	0.95908	0.97235	0.98303	0.99189	1
GRS	Eigenvalue	0.51868	0.27289	0.07766	0.0366	0.02504	0.01901	0.0171	0.00848	0.00767	0.00457	0.00399	0.00321	0.00208	0.00178	0.00092	0.00032
	Cumulative proportion	0.51868	0.79157	0.86923	0.90583	0.93087	0.94989	0.96699	0.97547	0.98314	0.98771	0.9917	0.99491	0.99699	0.99877	0.99968	1

[1]: 02GF: grains (G) of RILs grown under flooded (F) on 2002;
03GF: grains (G) of RILs grown under flooded (F) on 2003;
06GF: grains (G) of RILs grown under flooded (F) on 2006;
07GF: grains (G) of RILs grown under flooded (F) on 2007;
07GU: grains (G) of RILs grown under unflooded (U) on 2007;
08GF2: grains (G) of RILs grown under flooded (F) in the field site number 2 on 2008;
08GU1: grains (G) of RILs grown under unflooded (U) in the field site number 1 on 2008;
08GU3: grains (G) of RILs grown under unflooded (U) in the field site number 3 on 2008;
11GGH: grains (G) of RILs grown in a greenhouse (GH) condition on 2011;
15RTH: roots (RT) of RILs grown hydroponically (H) on 2015;
15SHH: shoots (SH) of RILs grown hydroponically (H) on 2015;
F: 02GF+03GF+06GF+07GF+08GF2;
U: 07GU+08GU1+08GU3;
FUGG: F+U+11GGH;
GRS: FUGG+15RTH+15SHH.

Supplementary Table 9. PC loadings of 16 PCs within each individual environment/tissue or aPCs across environments/tissues

02GF

Element	PC1	PC2	PC3	PC4	PC5	PC6	PC7	PC8	PC9	PC10	PC11	PC12	PC13	PC14	PC15	PC16
As	0.164112	-0.43512	0.09458	-0.24254	0.080732	-0.10808	0.245717	-0.20396	0.150105	-0.07548	0.311247	-0.17624	0.593957	-0.27967	0.055021	0.074836
Ca	0.01661	-0.3506	-0.47492	-0.02059	0.08546	0.060095	-0.06155	0.158464	-0.32395	-0.25234	-0.07162	-0.45445	-0.15563	-0.11494	-0.39439	-0.194334
Cd	-0.26642	0.263868	-0.19099	-0.31133	-0.1973	-0.08336	-0.15049	-0.26424	-0.03245	-0.58473	0.380535	0.163638	0.03233	0.20954	-0.12406	0.11293
Co	-0.24573	0.196052	-0.12298	-0.09834	0.021933	-0.19399	0.666708	-0.35515	0.210974	0.015941	-0.18914	-0.23837	-0.3267	-0.13732	0.023285	-0.064495
Cu	-0.07456	0.25623	0.20835	0.399287	0.155977	-0.48795	0.212613	0.322091	-0.09809	-0.05911	0.320243	0.019845	0.154884	-0.02372	-0.3602	-0.210266
Fe	0.280874	0.299094	-0.13618	-0.37158	0.228589	0.067838	-0.00666	0.018671	0.273893	0.30661	-0.05927	-0.16373	0.209911	0.44866	-0.41305	-0.049763
K	-0.3991	-0.14385	0.16116	-0.28244	-0.00847	-0.08396	0.040888	-0.0765	-0.32809	0.057097	-0.43568	0.280073	0.325706	0.116582	-0.07551	-0.441699
Mg	-0.37182	-0.1902	0.165443	-0.16804	0.201845	0.06258	-0.24171	0.152172	0.411318	0.073952	0.352709	-0.17815	-0.25664	0.063323	0.180109	-0.458929
Mn	-0.28091	0.052339	-0.36419	0.141997	-0.3576	0.113611	0.068973	-0.08946	-0.25977	0.572238	0.387647	-0.1209	0.180697	0.094796	0.0981	-0.030373
Mo	0.179172	-0.22191	0.09291	0.307581	-0.58177	-0.0124	-0.08578	-0.30077	0.376497	-0.03263	-0.07736	0.052605	-0.02603	0.080347	-0.35578	-0.306119
Ni	-0.15822	0.188674	-0.29389	0.11136	0.116789	-0.44073	-0.53495	-0.18687	0.223293	0.112468	-0.2518	-0.12211	0.215366	-0.3439	0.064861	0.044037
P	-0.44466	-0.12255	0.240478	-0.07752	0.003156	0.197257	-0.05439	0.047752	0.107283	0.196785	-0.00315	0.065824	-0.07787	-0.26508	-0.55806	0.486743
Rb	0.167038	-0.34545	0.060606	-0.08173	0.220258	-0.46809	-0.12418	-0.38061	-0.25319	0.265899	0.174876	0.173238	-0.38167	0.224692	-0.08267	0.126923
S	0.311075	0.307347	0.090676	-0.36962	-0.1358	0.081148	-0.08944	-0.05116	-0.18982	0.159073	0.184804	0.154003	-0.18562	-0.59701	-0.12562	-0.320028
Sr	0.018542	-0.22058	-0.52818	-0.09567	-0.00041	-0.12529	0.195508	0.35304	0.307819	0.058946	0.024163	0.611456	-0.04725	-0.09465	-0.01855	-0.009479
Zn	-0.02564	0.051549	-0.14921	0.378173	0.531449	0.445933	0.021261	-0.44324	0.002385	-0.05347	0.110187	0.27786	0.084143	-0.08065	-0.11044	-0.187648

03GF

Element	PC1	PC2	PC3	PC4	PC5	PC6	PC7	PC8	PC9	PC10	PC11	PC12	PC13	PC14	PC15	PC16
As	0.016541	-0.41289	0.05132	-0.2899	0.058947	-0.35608	0.035796	-0.15483	0.272002	-0.56766	0.080026	-0.21162	-0.13546	0.315065	0.136171	0.05899
Ca	0.012739	0.046645	0.601272	-0.16958	-0.01861	-0.07692	0.120482	0.082482	-0.11583	0.258618	-0.05984	-0.05065	-0.55671	0.126763	0.017215	-0.408728
Cd	-0.21913	0.419585	-0.0555	-0.10578	-0.07103	-0.04179	-0.25226	-0.30401	0.260015	-0.01178	0.426802	-0.26676	-0.18061	-0.39589	0.281108	-0.096816
Co	-0.16341	0.188392	-0.20323	-0.06759	0.052174	-0.69244	-0.22454	0.422722	0.143447	0.289196	0.067366	0.161594	0.03454	0.198238	-0.0707	0.016428
Cu	-0.21571	0.189404	-0.20894	0.383303	0.047273	-0.06704	0.645359	0.217047	0.065706	-0.27556	0.117594	-0.10505	-0.00843	0.012467	-0.00442	-0.389013
Fe	0.292965	0.171778	-0.18291	-0.3509	0.418959	0.055648	-0.01698	-0.04274	0.160961	-0.17667	-0.22665	0.485953	0.016333	-0.17125	0.139834	-0.391858
K	-0.40522	-0.09726	-0.04018	-0.28584	0.003377	0.092629	-0.20542	0.296063	-0.02261	-0.13327	-0.4671	-0.42097	0.134307	-0.287	-0.20458	-0.220568
Mg	-0.41621	-0.18735	0.087436	-0.07101	0.162647	0.234716	0.001605	-0.21215	0.389315	0.141802	0.278901	0.266117	0.060316	0.138197	-0.54162	-0.126704
Mn	-0.13404	0.381564	0.242564	-0.1119	-0.31325	-0.02774	-0.1504	0.064971	-0.34367	-0.55836	0.123331	0.339715	0.043844	0.051027	-0.266	0.059733
Mo	0.23285	-0.24292	0.012907	0.141662	-0.65418	-0.14111	-0.14071	-0.09891	0.214754	0.043949	-0.0258	0.10901	0.266679	-0.08992	-0.00097	-0.499288
Ni	-0.12681	0.457007	-0.00678	0.041476	-0.16112	0.033635	0.056178	-0.37347	0.279842	0.053881	-0.54584	-0.0762	0.056072	0.455733	0.047371	0.074314
P	-0.44546	-0.17435	-0.03602	0.086143	0.03264	0.24462	-0.25517	0.084066	-0.2122	0.001273	0.073366	0.198785	0.106262	0.346978	0.601448	-0.214508
Rb	-0.26151	-0.10271	-0.10734	-0.368	-0.07772	-0.32036	0.397846	-0.44101	-0.43227	0.215922	-0.01977	0.091553	0.213153	-0.14923	0.04937	-0.016848
S	0.284588	0.137422	-0.39581	-0.27034	-0.00979	0.188381	-0.11391	-0.0355	-0.28607	0.076491	0.276855	-0.35026	0.023398	0.436946	-0.2344	-0.295178
Sr	0.134803	0.180389	0.498869	-0.18051	0.172473	0.019635	0.143117	0.162123	0.132646	0.058422	0.198958	-0.19494	0.681491	0.074413	0.14939	-0.007536
Zn	0.02807	-0.00624	0.16637	0.481484	0.441813	-0.30765	-0.32977	-0.36596	-0.26125	-0.09797	-0.07955	-0.13595	0.134214	-0.01339	-0.1671	-0.241594

06GF

Element	PC1	PC2	PC3	PC4	PC5	PC6	PC7	PC8	PC9	PC10	PC11	PC12	PC13	PC14	PC15	PC16
As	0.393265	-0.08438	0.055309	-0.08569	0.236231	-0.44252	0.236565	-0.06649	0.072166	0.106793	-0.20715	-0.35947	-0.36833	0.194334	-0.39196	0.000831
Ca	0.041988	-0.13377	0.582725	0.024767	0.215068	0.169264	-0.05229	0.198828	0.273596	0.126567	-0.24494	-0.01485	-0.25293	0.034449	0.503182	-0.231393
Cd	-0.39958	-0.07058	0.028698	-0.29746	-0.00664	-0.26879	0.043342	0.073942	0.232888	-0.20102	-0.29664	-0.42768	0.384199	-0.36867	-0.01055	-0.131219
Co	-0.35375	-0.12005	0.075489	-0.07999	-0.15839	-0.28398	0.588064	-0.3188	-0.01589	0.001023	0.132179	0.167281	0.00861	0.39868	0.296765	-0.048618
Cu	-0.27482	0.159925	-0.27712	0.152308	0.395316	-0.11412	-0.35831	-0.384	-0.15235	-0.00079	0.150554	-0.34885	-0.26403	0.033076	0.309747	-0.139406
Fe	0.004401	0.410459	0.087438	-0.33728	0.030704	-0.09318	0.31272	0.203544	-0.32412	0.110712	0.232519	0.04171	-0.32487	-0.51191	0.14094	0.026106
K	-0.02234	-0.40141	-0.16836	-0.25796	0.046743	0.111634	-0.03644	-0.33512	0.161331	0.653967	-0.03949	0.099754	-0.01019	-0.29587	0.034297	0.247321
Mg	-0.02212	-0.44556	-0.18279	-0.11274	0.215686	-0.02322	0.020502	0.394311	-0.28859	-0.25865	-0.036	-0.09831	-0.06085	0.116962	0.276177	0.545071
Mn	-0.28618	-0.1675	0.33904	-0.0841	-0.27197	-0.18497	-0.34268	0.101235	0.238471	-0.03618	0.535062	-0.09329	-0.29539	0.034857	-0.23183	0.191771
Mo	0.278644	-0.00142	0.099505	0.314429	-0.3941	-0.58346	-0.24098	-0.02029	-0.18922	0.170108	-0.1151	0.006082	0.160254	-0.15837	0.342279	0.115869
Ni	-0.39451	0.077693	0.014538	-0.07685	0.24717	-0.32528	-0.26072	0.193621	-0.16916	0.151632	-0.35346	0.549982	-0.08509	0.113015	-0.23926	-0.059723
P	0.010745	-0.45826	-0.23423	-0.03556	-0.18582	0.001296	-0.01109	0.296977	-0.29715	0.164467	0.163384	-0.08525	-0.06935	0.006226	-0.03761	-0.674787
Rb	0.350628	-0.09211	-0.01664	-0.23427	0.442456	-0.29479	-0.0929	-0.02458	0.233965	-0.18199	0.430681	0.314973	0.323592	-0.05454	0.113605	-0.168431
S	0.065748	0.362053	-0.09112	-0.45229	-0.10481	0.025318	-0.18193	0.247546	0.029822	0.382158	0.050127	-0.23557	0.24386	0.506296	0.151628	0.044549
Sr	-0.02592	-0.11188	0.55926	-0.01842	0.22682	0.115399	-0.03161	-0.21429	-0.57224	0.11638	0.10809	-0.16751	0.371564	0.038622	-0.20239	0.02752
Zn	-0.19752	0.075552	-0.05811	0.555025	0.28945	-0.08141	0.281689	0.38486	0.18761	0.390307	0.247365	-0.12393	0.215381	-0.04369	-0.07294	0.090949

07GF

Element	PC1	PC2	PC3	PC4	PC5	PC6	PC7	PC8	PC9	PC10	PC11	PC12	PC13	PC14	PC15	PC16
As	0.236193	-0.32348	0.192944	-0.0728	0.078555	-0.40099	0.130522	-0.32771	0.217747	0.189198	0.242631	-0.55639	0.13597	0.004471	-0.02056	-0.187285
Ca	-0.19524	-0.39135	-0.34848	-0.22878	-0.01331	-0.10672	-0.03988	0.150426	0.085326	-0.07629	0.173078	0.289221	-0.176	0.003932	0.087751	-0.658608
Cd	-0.03389	0.237367	-0.12581	-0.42779	-0.41696	-0.0614	0.304978	-0.15797	-0.47187	-0.16776	-0.09052	-0.24827	-0.03254	-0.3283	0.082243	-0.108818
Co	0.066399	-0.15924	0.271777	-0.36301	0.09508	0.463814	0.296057	-0.4673	0.290242	-0.14264	-0.16823	0.301144	0.080544	0.025591	0.058656	-0.015895
Cu	-0.14864	0.448401	-0.08019	-0.08614	0.107211	-0.18041	-0.09814	-0.04907	0.476189	0.318117	-0.10588	0.124671	0.146172	-0.56042	0.068789	-0.109704
Fe	-0.11753	-0.06603	0.277199	-0.4031	0.375851	0.26535	-0.1163	0.312971	-0.26616	0.495009	-0.12509	-0.22212	-0.16879	-0.01237	0.067371	-0.072856
K	0.462983	0.019139	-0.19742	-0.07789	0.08422	0.173599	-0.10372	-0.00438	0.166992	-0.1126	0.062702	-0.11233	-0.61411	-0.27247	-0.42351	0.066225
Mg	0.464335	0.053146	-0.14026	-0.09801	0.066689	-0.00031	0.19613	0.209738	-0.19119	0.20366	-0.01883	0.252686	0.527836	0.041985	-0.46212	-0.170957
Mn	-0.1717	-0.08145	-0.47404	-0.11115	-0.18789	0.480941	-0.16993	0.073898	0.279274	0.007775	0.003613	-0.45712	0.342631	0.102808	-0.07044	0.076918
Mo	-0.03216	-0.1779	0.1133	0.341859	-0.56839	0.252993	0.032312	-0.1769	-0.06143	0.578728	0.047627	0.130819	-0.15275	-0.11652	-0.10137	-0.113734
Ni	-0.11761	0.414165	-0.07299	-0.33769	-0.15567	-0.14803	0.157691	-0.0562	0.187111	0.249519	0.297919	0.08793	-0.23099	0.585393	-0.13993	0.108261
P	0.450473	0.063216	-0.21932	0.085588	0.036563	0.169718	0.296727	0.199443	0.057327	0.144416	0.262303	0.023025	-0.04128	-0.0246	0.690153	0.087133
Rb	0.299175	0.100984	-0.04403	-0.19215	-0.05465	0.010498	-0.73967	-0.39271	-0.22472	0.044277	0.127059	0.140349	0.117129	0.094097	0.209745	-0.046162
S	0.020578	0.01907	0.507735	-0.18433	-0.31359	0.103722	-0.14712	0.402364	0.159731	-0.24631	0.511723	0.037936	0.146044	-0.20216	-0.05156	0.021551
Sr	-0.1375	-0.44596	-0.22528	-0.27502	0.010706	-0.22242	0.015987	-0.0319	-0.07715	0.168085	0.173884	0.239905	0.052437	-0.24013	-0.0406	0.648783
Zn	-0.28029	0.169222	-0.09092	0.211837	0.401349	0.263559	0.12837	-0.29234	-0.27265	-0.02286	0.612744	-0.00886	0.061736	-0.16273	-0.14164	-0.080194

07GU

Element	PC1	PC2	PC3	PC4	PC5	PC6	PC7	PC8	PC9	PC10	PC11	PC12	PC13	PC14	PC15	PC16
As	0.052203	-0.43641	0.161681	-0.03385	0.191062	-0.15963	0.537824	-0.04698	0.309705	-0.10159	0.024068	-0.06672	-0.31994	-0.45839	-0.01445	0.046062
Ca	0.354763	0.253104	0.296653	-0.13177	-0.06489	-0.25108	0.112227	0.046727	0.035329	0.072129	0.222939	0.151973	0.229244	-0.09976	0.672313	-0.17009
Cd	0.231301	0.150405	-0.41093	-0.13235	0.327622	0.153532	-0.02011	-0.18019	0.232326	-0.27434	0.201481	-0.55161	0.262346	-0.06533	0.016521	-0.161979
Co	0.157982	0.224609	-0.34304	0.216713	0.449689	0.004309	0.260484	-0.12383	0.157554	-0.00207	-0.32197	0.559604	0.002317	0.177757	0.035207	0.005348
Cu	-0.04026	-0.0055	-0.4168	-0.12975	-0.13989	-0.46936	0.341635	-0.05605	-0.64342	-0.14086	0.022466	-0.03134	0.04269	-0.05795	-0.0523	-0.069495
Fe	-0.30292	-0.23448	-0.1321	-0.30177	-0.11771	-0.23622	0.062501	0.003545	0.334854	0.308547	-0.4908	-0.14502	0.386844	0.137708	0.141066	-0.115814
K	0.432274	-0.17788	-0.09539	-0.00063	-0.08764	0.175123	-0.01847	0.059502	-0.11568	0.293252	-0.03976	0.127751	0.489701	-0.39826	-0.22852	0.402805
Mg	0.36673	-0.32234	-0.11159	0.113444	-0.22504	0.074436	0.056966	-0.1268	-0.03006	-0.1393	-0.1535	-0.21473	-0.1969	0.463767	0.365036	0.4286
Mn	0.235703	0.379286	0.000356	-0.30674	0.085414	-0.05952	-0.09697	-0.18114	-0.09384	0.480439	-0.28895	-0.24939	-0.48802	-0.14954	-0.04378	0.075385
Mo	-0.09692	-0.25515	0.187071	0.056256	0.341515	-0.30979	-0.30084	-0.6839	-0.11037	0.140785	0.209016	0.064589	0.128213	0.079356	-0.00464	0.117854
Ni	0.023732	0.119566	-0.37074	-0.02441	-0.52463	-0.25218	-0.21647	-0.23829	0.460484	-0.08276	0.222488	0.254868	-0.17	-0.16619	-0.08147	0.090029
P	0.374556	-0.31606	-0.0386	0.236465	-0.20494	0.123779	-0.03003	-0.16726	-0.06023	0.209566	-0.05799	0.028641	-0.07762	0.056032	-0.12963	-0.733832
Rb	0.184233	-0.10384	-0.06351	0.33901	0.220132	-0.51225	-0.47595	0.428307	0.034635	-0.1326	-0.22468	-0.12768	-0.06299	-0.15836	0.026501	-0.012734
S	0.001163	-0.30771	-0.33364	-0.31453	0.25399	0.024334	-0.09278	0.375057	0.016118	0.370876	0.475588	0.160775	-0.19564	0.212394	0.086288	-0.004242
Sr	0.351515	0.09441	0.307927	-0.26213	-0.0025	-0.32611	0.148138	0.111147	0.173596	-0.11171	0.082003	0.002254	0.098359	0.449836	-0.54912	0.020793
Zn	-0.12213	0.210545	-0.04977	0.59992	-0.06188	-0.16676	0.317696	0.057206	0.123994	0.468768	0.274755	-0.31028	0.069033	0.126866	-0.05568	0.108121

08GF2

Element	PC1	PC2	PC3	PC4	PC5	PC6	PC7	PC8	PC9	PC10	PC11	PC12	PC13	PC14	PC15	PC16
As	-0.32102	-0.04799	0.267042	-0.15473	0.068567	-0.08738	0.222684	-0.53419	0.02523	-0.19425	0.139684	0.601471	-0.06366	0.113874	-0.12193	0.023908
Ca	-0.31037	-0.38573	-0.20028	0.081499	-0.14269	0.03838	0.293705	0.022385	0.27068	0.143968	-0.04625	0.078431	0.111724	-0.2297	0.65526	0.051289
Cd	-0.00931	0.002403	-0.46865	0.105016	0.39256	-0.23737	-0.10777	-0.0661	0.014704	-0.56042	0.430765	-0.10887	-0.03305	-0.12595	0.108858	0.048151
Co	-0.06119	0.135894	-0.13623	0.387729	-0.14888	-0.65799	-0.08907	-0.38394	-0.10847	0.384633	-0.04812	-0.11901	0.090377	0.108483	0.010405	0.046939
Cu	0.264344	0.304177	-0.24326	-0.19369	-0.05005	0.2503	-0.09862	-0.2273	0.207967	0.400818	0.417162	0.112108	-0.36545	0.141254	0.242676	-0.107068
Fe	0.017912	-0.10614	0.263668	0.488175	0.145932	0.409612	-0.28273	-0.32972	-0.3615	-0.09908	-0.09532	-0.08761	-0.20455	0.003238	0.322026	0.006398
K	-0.39461	0.326588	-0.12332	0.023824	-0.09474	-0.00399	0.015057	0.04893	-0.14921	0.022463	-0.14644	0.029139	-0.40564	-0.59973	-0.13491	-0.350365
Mg	-0.42654	0.260354	-0.04728	0.093889	0.10174	0.110924	-0.13842	0.095984	0.195751	-0.09262	-0.08078	-0.063	0.259168	0.504303	0.116018	-0.543924
Mn	-0.07172	-0.34167	-0.40545	-0.01171	0.050709	0.051068	-0.25341	0.243239	-0.50026	0.244086	0.05405	0.478043	0.081898	0.121762	-0.10786	-0.102848
Mo	-0.05158	-0.28085	0.231773	-0.42535	0.306135	-0.40706	-0.22917	0.090997	-0.01961	0.063499	-0.18564	-0.14226	-0.44534	0.141902	0.258674	-0.138202
Ni	0.226702	-0.00292	-0.41552	-0.05543	0.225504	0.109771	-0.10092	-0.31092	0.289092	-0.06417	-0.69284	0.153298	-0.00388	-0.02554	-0.0996	0.036092
P	-0.38218	0.336748	-0.04505	-0.03976	-0.10081	0.029903	-0.32839	0.220997	0.053743	-0.05554	-0.10597	0.123019	-0.14238	0.149798	0.114625	0.693543
Rb	-0.21825	0.114427	-0.21803	-0.40464	0.156737	0.184325	0.367084	-0.25807	-0.475	0.103355	-0.06751	-0.4092	0.132538	0.123855	0.041409	0.158665
S	0.204237	0.239303	-0.03573	0.310967	0.211397	-0.11783	0.588951	0.320283	-0.15358	-0.04694	-0.15659	0.221367	-0.31221	0.274384	0.149986	0.021275
Sr	-0.28243	-0.40245	-0.15793	0.250922	0.009104	0.162159	0.1384	-0.01336	0.256773	0.119361	0.099208	-0.27241	-0.42138	0.243623	-0.46241	0.098452
Zn	0.120621	-0.10417	-0.19416	-0.11717	-0.72725	-0.05859	0.004291	-0.08977	-0.16536	-0.44948	-0.10879	-0.05432	-0.22175	0.25426	0.099614	-0.121905

08GU1

Element	PC1	PC2	PC3	PC4	PC5	PC6	PC7	PC8	PC9	PC10	PC11	PC12	PC13	PC14	PC15	PC16
As	0.0995	-0.25369	0.087243	-0.04635	0.508353	-0.35408	0.058547	-0.33569	0.420974	-0.43257	0.111601	-0.09027	-0.08369	0.083351	0.02199	-0.129192
Ca	0.26779	-0.45575	-0.1425	0.101708	0.025695	0.098455	-0.15706	0.126326	0.078935	0.14814	0.062138	-0.01507	0.157249	-0.12626	-0.74754	-0.084852
Cd	0.049318	-0.01445	0.190613	0.392884	0.280404	-0.06376	0.715613	0.395896	-0.00225	0.180536	-0.12138	-0.05475	-0.00651	-0.02651	-0.00172	-0.063745
Co	0.167291	0.113785	-0.16787	0.461875	-0.11869	-0.44055	-0.03017	-0.34664	-0.45599	0.028287	0.192812	-0.21429	-0.14479	-0.25874	-0.02786	-0.088345
Cu	-0.25868	0.06377	0.061268	0.305928	0.395295	0.349715	-0.22951	-0.36194	0.077462	0.30541	-0.22502	-0.25219	0.347265	-0.14817	0.104003	-0.050608
Fe	-0.29615	-0.29132	0.183149	0.170241	0.028062	0.216667	-0.17901	0.123381	-0.27823	-0.38992	-0.3722	-0.00742	-0.50016	-0.1403	-0.05178	-0.171298
K	0.430651	0.152418	0.200881	0.054812	0.033355	-0.0508	-0.14944	0.035854	0.204335	-0.06176	-0.27396	0.366582	-0.00334	-0.6203	0.116209	0.259515
Mg	0.369421	0.137876	0.320336	-0.04339	0.205888	0.327769	0.0039	-0.14309	-0.21827	-0.01829	0.144248	-0.26321	-0.27815	0.260184	-0.16315	0.509868
Mn	0.250809	-0.38045	-0.18646	0.202777	0.013791	0.027843	0.039318	-0.27787	-0.17938	0.151483	-0.28555	0.499483	0.016556	0.435805	0.231708	0.081874
Mo	-0.06264	0.137898	-0.52551	-0.2032	0.003829	0.238656	0.401519	-0.3388	0.187674	0.102482	-0.17701	0.002878	-0.41365	-0.21793	-0.17271	0.033715
Ni	-0.07914	0.250429	-0.26624	0.444691	0.13772	0.354015	-0.0435	0.112253	0.086385	-0.36618	0.466498	0.375681	0.029591	0.038542	-0.02484	0.017708
P	0.38332	0.335469	0.173007	-0.15846	0.10724	0.183633	-0.05321	-0.05899	-0.09837	0.095331	-0.01474	0.130057	-0.09978	0.107836	-0.04115	-0.758141
Rb	0.19748	0.310505	-0.21338	0.321457	-0.21209	-0.10393	-0.1854	0.162001	0.350925	-0.17237	-0.45569	-0.31519	-0.03156	0.361562	-0.08677	0.014468
S	-0.24354	0.026033	0.404949	0.257285	-0.25671	-0.10821	-0.09005	-0.18124	0.408911	0.38921	0.181122	0.223446	-0.39205	0.116011	-0.15037	-0.008126
Sr	0.290786	-0.36124	-0.15717	0.085615	-0.12223	0.255564	-0.10313	0.188105	0.231559	0.173222	0.274854	-0.32843	-0.2459	-0.12713	0.519767	-0.113243
Zn	-0.09232	0.130402	-0.28519	-0.10116	0.539596	-0.28795	-0.36224	0.352024	-0.08544	0.346779	0.005226	0.103485	-0.31899	0.066902	0.013136	0.094833

08GU3

Element	PC1	PC2	PC3	PC4	PC5	PC6	PC7	PC8	PC9	PC10	PC11	PC12	PC13	PC14	PC15	PC16
As	0.196996	0.089345	0.412184	-0.06982	0.246092	-0.17214	0.375088	-0.37325	0.43694	-0.18534	0.288319	0.0047	-0.08431	-0.30225	-0.02698	-0.025138
Ca	-0.324	-0.24373	0.250367	-0.17521	-0.32575	-0.24822	-0.08747	0.117025	0.159799	0.119774	0.155029	-0.00501	-0.17277	0.15384	-0.57113	-0.330944
Cd	-0.05713	-0.2745	0.062844	0.374344	-0.28705	-0.00682	0.442063	-0.27583	-0.52137	0.198885	0.135915	0.132027	0.094548	-0.22769	-0.09762	0.075916
Co	-0.05231	0.114318	0.400572	0.504994	0.23704	-0.14719	0.177115	0.17274	0.003903	0.124043	-0.1796	-0.33669	-0.09473	0.479767	-0.02096	0.170438
Cu	0.318675	0.225691	0.130048	-0.10616	-0.38227	0.087745	0.166778	0.377341	-0.1693	-0.31036	-0.19007	-0.39542	-0.15979	-0.32946	-0.19722	0.069685
Fe	0.223897	0.344614	0.274001	-0.18889	0.016454	0.129825	-0.09215	-0.15063	-0.03706	0.664147	-0.382	0.199067	0.001976	-0.12763	-0.15552	-0.059644
K	0.230102	-0.45934	0.003423	0.066438	0.117492	-0.12999	-0.03852	0.036607	-0.04221	-0.02239	-0.32523	0.195103	-0.69054	-0.08848	0.213486	-0.147447
Mg	0.434679	-0.27422	0.056472	0.053715	0.002316	0.141268	-0.03397	0.094697	0.010728	0.230296	0.226506	-0.3687	0.277368	0.064292	0.222303	-0.573428
Mn	-0.06526	-0.25099	0.425248	0.252517	-0.02708	0.355002	-0.17942	0.109289	0.164934	-0.33879	-0.32111	0.327612	0.37501	-0.12559	-0.05595	-0.072733
Mo	-0.18525	0.066207	0.207596	-0.1406	0.518532	0.41351	-0.02638	0.235274	-0.42647	-0.0362	0.355478	0.055058	-0.2253	-0.09241	-0.13612	-0.110043
Ni	0.158544	0.167274	0.289011	0.092828	-0.11635	-0.25965	-0.61714	-0.3956	-0.37336	-0.24966	0.144566	-0.06576	-0.04823	0.061657	0.054723	-0.01893
P	0.35795	-0.34449	-0.06889	0.108599	0.121022	0.041164	-0.31147	0.119646	0.157687	0.207029	0.252897	-0.06191	0.026494	-0.12913	-0.3537	0.575321
Rb	0.065031	-0.16734	0.059763	-0.30191	0.353166	-0.60442	0.087407	0.237399	-0.29345	-0.05644	-0.20393	0.062928	0.412499	-0.10259	-0.05809	0.009803
S	0.4387	-0.00356	0.097584	-0.26483	-0.17008	0.112333	0.245318	0.047005	-0.10597	-0.16197	0.149235	0.421393	0.008109	0.609287	-0.03673	0.11824
Sr	-0.24441	-0.17885	0.42711	-0.32949	-0.26223	0.003087	-0.03998	0.170549	0.020572	0.205978	0.146852	-0.07852	-0.01346	-0.06246	0.571533	0.341347
Zn	0.091549	0.338038	-0.0101	0.375816	-0.11966	-0.28557	-0.0758	0.489818	0.093136	0.110838	0.321281	0.43927	-0.03152	-0.189	0.15462	-0.129275

11GGH

Element	PC1	PC2	PC3	PC4	PC5	PC6	PC7	PC8	PC9	PC10	PC11	PC12	PC13	PC14	PC15	PC16
As	0.345197	-0.22447	0.116189	-0.3162	0.209158	-0.12298	0.044528	-0.0589	0.195023	-0.05068	0.015871	-0.43083	-0.58659	-0.08056	0.269873	-0.052615
Ca	0.29906	0.161931	-0.4178	0.021968	-0.2925	0.133611	-0.0669	-0.27726	-0.03154	-0.07934	0.322458	0.27508	-0.10355	-0.4269	0.313431	0.21541
Cd	0.21132	-0.21939	0.164367	0.12872	0.345019	0.304143	-0.27491	0.319302	-0.54425	-0.01221	0.199518	-0.16812	0.185905	-0.2271	0.08581	0.128947
Co	0.140404	0.151067	0.361459	0.040164	0.404991	0.058443	0.457741	-0.34264	0.160724	0.337883	0.335947	0.125033	0.179728	-0.09323	-0.08722	0.117249
Cu	-0.09127	-0.08252	0.538823	0.088252	-0.05227	-0.20763	-0.21651	-0.45057	-0.14073	-0.57048	0.004322	0.183381	-0.00621	-0.08248	0.039962	-0.060508
Fe	0.194683	0.478536	0.210974	0.049348	-0.16959	0.16312	0.025095	0.075582	0.027532	-0.02692	-0.36464	-0.24532	-0.04926	-0.52757	-0.35511	-0.151521
K	0.126912	-0.4066	0.024795	0.264907	-0.14898	-0.14843	-0.27014	-0.30405	-0.13747	0.599497	-0.20089	0.126904	-0.19064	-0.11048	-0.21423	-0.094489
Mg	0.17691	-0.44462	0.104721	0.131019	-0.10046	0.276407	0.024755	0.20334	0.512457	-0.23226	-0.07458	0.177839	0.008877	-0.05757	-0.28536	0.419356
Mn	0.426934	-0.06071	-0.07189	0.187668	-0.01323	0.017007	0.247613	-0.20766	-0.07569	-0.07353	-0.55073	-0.12192	0.384805	0.210053	0.377383	0.069544
Mo	0.133112	-0.2101	-0.07085	0.015091	-0.12888	-0.50975	0.567995	0.282431	-0.35427	-0.14948	0.061346	0.14032	-0.05971	-0.19764	-0.20015	0.008176
Ni	0.184001	0.347418	0.051562	0.384742	0.320444	-0.03693	-0.06464	0.25127	-0.032	-0.03348	-0.16712	0.457566	-0.46996	0.228517	0.094813	0.043355
P	0.398859	-0.04562	0.277201	-0.15493	-0.31163	0.082481	-0.05696	0.273312	0.140101	0.088705	0.219281	0.271478	0.175941	0.140343	0.14659	-0.57854
Rb	0.160662	0.155816	-0.01163	0.075781	0.133008	-0.65767	-0.39377	0.183454	0.327381	0.075766	0.074614	-0.09806	0.338082	-0.15502	0.061799	0.186668
S	0.286077	0.218295	0.179504	-0.47048	-0.2427	-0.04288	-0.14021	-0.04922	-0.27883	0.103517	-0.0005	0.028069	-0.0419	0.38172	-0.24864	0.485365
Sr	0.364353	0.014345	-0.39044	0.12895	0.229101	0.004243	-0.1067	-0.24317	-0.00045	-0.28797	0.219293	-0.14652	0.04726	0.252797	-0.5241	-0.28572
Zn	-0.00746	0.10429	0.182657	0.576708	-0.42214	-0.01618	0.099489	0.042904	0.006811	0.034314	0.357016	-0.45142	-0.133	0.248188	0.075241	0.124486

15RTH

Element	PC1	PC2	PC3	PC4	PC5	PC6	PC7	PC8	PC9	PC10	PC11	PC12	PC13	PC14	PC15	PC16
As	0.133952	-0.26691	0.279072	-0.29484	0.328305	-0.43669	0.116212	-0.11808	0.195391	-0.005	0.531561	-0.13784	0.261129	0.109867	0.004049	0.006524
Ca	0.411746	-0.1172	-0.09486	0.143057	-0.1223	-0.22044	-0.09206	0.008737	0.251296	0.100213	-0.14547	-0.18906	-0.06694	-0.35019	-0.66374	-0.131901
Cd	-0.27799	-0.39139	-0.20153	-0.01068	0.033112	-0.00488	-0.11933	0.105196	0.193148	-0.07689	0.139413	0.282069	-0.22315	-0.10738	0.105183	-0.698411
Co	-0.26549	-0.4152	-0.12766	0.01917	-0.04502	-0.05601	-0.10169	0.11816	0.129368	-0.05544	0.168045	0.180061	-0.3688	-0.14291	-0.09683	0.681694
Cu	-0.20852	-0.31916	-0.36124	0.09757	-0.08951	0.352198	-0.04641	-0.05099	0.099097	-0.17068	0.033337	-0.2645	0.666608	0.059308	-0.12632	0.063043
Fe	0.212173	-0.39087	0.145446	-0.19727	-0.03804	0.103649	-0.06905	0.14267	0.105016	-0.09116	-0.39378	-0.17043	-0.21999	0.671153	-0.04538	-0.021695
K	-0.08379	0.239546	-0.19631	-0.50144	0.141838	-0.08777	-0.25421	0.671823	0.08405	0.195899	-0.09706	0.017488	0.196798	-0.06238	-0.0453	0.044643
Mg	-0.06231	0.263373	0.266661	0.488889	-0.001	-0.14313	-0.30443	0.344149	0.270767	-0.51718	0.133864	-0.0458	0.049979	0.147358	0.00206	-0.00068
Mn	0.094213	-0.08491	0.000223	0.236519	0.876283	0.227085	-0.17044	-0.06696	0.004956	0.06686	-0.22701	0.060136	-0.00888	-0.109	0.001685	0.058232
Mo	0.247011	-0.26912	0.214656	-0.15115	0.001384	0.125805	0.227006	0.326819	-0.41974	-0.49884	-0.06219	-0.0532	0.035266	-0.42652	0.08924	-0.024819
Ni	-0.15218	-0.28466	0.187151	0.310029	-0.08293	-0.27272	-0.35964	0.151633	-0.57772	0.393745	0.016875	-0.14182	0.122108	0.056955	0.000856	-0.061054
P	0.312807	0.078059	-0.01935	-0.07811	-0.05156	0.533619	-0.357	0.0369	-0.07487	0.115273	0.57202	-0.24495	-0.25599	0.014079	0.007953	-0.038408
Rb	0.05701	0.131959	-0.64266	-0.00344	0.189841	-0.34946	0.008453	-0.06841	-0.36567	-0.33358	0.099611	-0.23069	-0.21541	0.214339	-0.04998	-0.020792
S	0.404705	-0.03587	-0.10857	-0.00244	-0.08244	-0.00599	-0.20321	-0.06069	-0.17677	-0.09389	0.098357	0.760636	0.270573	0.208365	-0.15134	0.059098
Sr	0.389045	-0.13055	-0.2135	0.061812	-0.15273	-0.20911	-0.28389	-0.08957	0.23631	0.042156	-0.16944	-0.10611	0.078363	-0.20333	0.686352	0.101831
Zn	0.236781	-0.07448	-0.20749	0.41036	0.03138	0.04654	0.574364	0.46495	0.083855	0.306833	0.184105	0.047547	0.004188	0.160704	0.120033	0.011542

15SHH

Element	PC1	PC2	PC3	PC4	PC5	PC6	PC7	PC8	PC9	PC10	PC11	PC12	PC13	PC14	PC15	PC16
As	-0.30248	0.037728	-0.0452	0.331964	-0.05254	0.344019	-0.13116	0.479189	-0.27354	0.227794	-0.24469	0.312836	-0.2886	0.137569	-0.0139	-0.200094
Ca	0.268373	-0.43305	-0.1878	-0.05506	-0.02721	-0.04893	-0.2702	-0.11003	-0.15981	0.043392	-0.03893	0.213875	-0.2172	-0.0454	-0.64286	0.276949
Cd	-0.44096	-0.11663	-0.03013	-0.10218	-0.01853	-0.10299	-0.00606	-0.15825	-0.18085	-0.15296	0.07618	-0.03525	-0.38569	0.281308	0.331551	0.586176
Co	-0.26045	-0.24367	0.433805	0.171531	-0.07175	-0.15793	-0.12416	0.044009	0.048811	-0.2674	-0.13371	-0.12853	-0.22071	-0.6666	-0.00389	-0.079828
Cu	0.072398	-0.08687	0.406908	-0.03748	0.159585	0.718127	-0.14291	-0.34086	0.079252	0.106341	0.329176	0.006004	-0.12211	0.01256	0.036552	-0.007984
Fe	0.026798	-0.33339	0.469821	0.247892	-0.12729	-0.20577	-0.23085	0.229446	0.169505	0.057603	0.029611	-0.21822	0.258353	0.537045	-0.06465	0.03975
K	0.0411	0.229774	0.131097	-0.52506	-0.33663	0.031549	-0.39987	0.117828	0.383878	-0.20336	-0.20825	0.333245	-0.10049	0.083139	0.064487	-0.015591
Mg	-0.03385	-0.10289	-0.17397	0.306001	-0.6684	0.278786	0.137978	-0.27399	-0.08205	-0.41249	-0.038	0.064642	0.242645	0.056781	-0.00821	-0.033303
Mn	-0.41682	-0.12016	-0.15439	-0.21358	-0.00489	-0.15263	-0.03367	-0.15494	0.02956	-0.11988	0.350321	-0.102	-0.20312	0.226738	-0.26216	-0.619792
Mo	0.01544	-0.45321	-0.13075	-0.09156	0.085058	0.08787	0.302111	0.45536	0.264729	-0.18075	0.409156	0.364247	0.096518	-0.10661	0.164988	0.067539
Ni	0.10179	-0.05834	0.243048	-0.42841	-0.50487	0.012493	0.320441	0.206402	-0.37764	0.349853	0.139254	-0.20516	-0.08681	-0.09207	-0.02759	-0.004927
P	0.261272	0.163302	0.250393	0.32091	-0.13636	-0.3186	0.343424	-0.22335	0.188781	0.111863	0.127528	0.40376	-0.44945	0.119764	0.015817	-0.086201
Rb	0.189526	0.356522	0.163623	0.076799	0.071535	-0.1244	-0.26978	0.19638	-0.50485	-0.4182	0.468005	0.128711	0.073851	-0.0076	-0.03359	0.019209
S	0.30448	-0.16627	0.150789	-0.17924	0.271568	0.135234	0.324662	0.069357	-0.18832	-0.48766	-0.4239	-0.1268	-0.21989	0.263313	0.012025	-0.188832
Sr	0.288884	-0.37904	-0.15062	-0.0177	-0.0412	-0.16116	-0.3523	-0.19237	-0.24847	0.157052	-0.03199	0.111616	-0.01652	-0.03836	0.60186	-0.311806
Zn	-0.31736	-0.08751	0.323938	-0.18939	0.167184	-0.12054	0.162353	-0.26321	-0.27233	0.076667	-0.18802	0.531455	0.455231	0.01886	-0.07991	-0.029729

F

Element	aPC1	aPC2	aPC3	aPC4	aPC5	aPC6	aPC7	aPC8	aPC9	aPC10	aPC11	aPC12	aPC13	aPC14	aPC15	aPC16
As	0.332106	-0.32582	0.160904	-0.05013	0.117342	-0.0888	0.243449	-0.17908	0.001009	-0.0054	-0.39486	-0.28929	0.616121	0.022018	0.122718	0.085838
Ca	-0.29028	-0.1298	0.452074	-0.16591	0.090383	-0.16891	-0.05261	-0.07064	0.186467	0.153815	0.235822	-0.39565	-0.03225	-0.45295	-0.37976	0.039142
Cd	-0.16742	0.24585	-0.17643	-0.41885	0.069925	0.213529	0.129996	0.401731	0.119506	0.202201	-0.59522	-0.16345	-0.11973	-0.04584	-0.14205	0.058401
Co	-0.11586	-0.06586	-0.07372	-0.22427	-0.50253	0.225786	0.589848	-0.39685	0.294379	0.000365	0.111104	0.094672	-0.08545	-0.02099	0.041835	0.010863
Cu	0.006695	0.37687	-0.38785	0.075908	0.349078	-0.06428	0.174305	-0.10615	0.243435	-0.5039	0.099483	0.024835	0.256607	-0.29341	-0.24179	0.00228
Fe	0.176253	0.235025	0.168549	-0.1297	-0.39269	-0.42151	0.28805	0.250596	-0.49645	-0.32027	6.54E-05	-0.09337	-0.0732	-0.05534	-0.14912	0.04913
K	0.293161	-0.2423	-0.22041	-0.37752	0.154194	0.008973	-0.07254	-0.26811	-0.20031	0.040468	0.044155	0.040135	-0.13718	0.248237	-0.56272	-0.347609
Mg	-0.17525	-0.45736	-0.29641	-0.20376	-0.06652	-0.20008	-0.08592	0.132811	-0.09596	-0.14604	-0.10111	0.165984	-0.01205	-0.50958	0.316017	-0.365044
Mn	-0.21689	0.149465	0.118555	-0.38156	0.259322	0.417016	0.124896	0.048132	-0.45454	0.000723	0.380398	-0.01733	0.307146	0.021721	0.243905	-0.077494
Mo	0.018562	-0.23128	0.294785	0.373661	0.016627	0.56087	0.128866	0.094214	-0.19574	-0.28239	-0.23957	0.195835	-0.11079	-0.24371	-0.29861	-0.068383
Ni	0.415383	0.069395	-0.01542	-0.23365	0.145979	0.104276	-0.14354	-0.31403	-0.15003	0.020038	-0.04728	0.065819	-0.34674	-0.40002	0.205261	0.51435
P	-0.32721	-0.36532	-0.30587	-0.03458	-0.11477	-0.02068	-0.08778	0.105804	-0.16115	-0.05079	0.079612	0.136004	0.219025	0.117159	-0.28178	0.661622
Rb	0.180256	-0.24446	1.73E-05	0.079376	0.434638	-0.18075	0.568134	0.370651	0.094627	0.23512	0.241852	0.194893	-0.21829	0.02662	0.032995	0.097712
S	0.379981	0.1441	0.033921	-0.08495	-0.29378	0.098008	-0.15344	0.268195	0.155345	0.351002	0.171236	0.40021	0.420141	-0.28484	-0.19691	-0.043014
Sr	-0.16811	0.033761	0.46729	-0.34342	0.169166	-0.22069	-0.07896	-0.08275	0.205248	-0.26579	-0.19944	0.592457	0.032018	0.190447	0.015634	0.05292
Zn	-0.28998	0.22647	-0.06034	0.255268	0.10266	-0.23309	0.184592	-0.37857	-0.375	0.473411	-0.24801	0.273784	0.086847	-0.17215	-0.10624	-0.062314

U

Element	aPC1	aPC2	aPC3	aPC4	aPC5	aPC6	aPC7	aPC8	aPC9	aPC10	aPC11	aPC12	aPC13	aPC14	aPC15	aPC16
As	0.208983	-0.1335	0.247619	-0.2199	0.337861	-0.37407	0.141843	-0.04564	0.571617	-0.16487	0.244243	-0.07089	0.224154	-0.23041	0.174171	0.016683
Ca	0.154977	-0.19711	-0.59265	-0.02593	0.053704	-0.22377	-0.09303	0.247851	-0.03545	-0.22033	-0.0343	-0.09198	0.161955	-0.2286	-0.5678	0.043102
Cd	0.049325	0.192001	-0.31899	0.402419	-0.30047	0.123726	0.285429	-0.06327	0.597829	0.051738	0.124049	0.297635	-0.14793	0.058218	-0.06026	0.113954
Co	0.176064	-0.22477	0.140756	0.014231	-0.51813	-0.27926	0.40983	-0.19218	-0.03678	0.214529	-0.22954	-0.46362	0.104786	0.090204	-0.10685	0.039847
Cu	-0.10634	0.253239	0.194951	0.320077	-0.08441	-0.47551	0.069036	0.546883	-0.21908	0.096452	0.393271	0.043176	0.096395	0.132151	-0.00233	0.080238
Fe	-0.3255	0.016431	0.115726	0.131901	0.311736	-0.38408	0.033473	-0.19878	0.063062	0.281148	-0.51881	0.376802	0.088044	0.025959	-0.25282	0.105609
K	0.399848	-0.09678	0.012737	0.312294	0.064408	0.096967	-0.08023	-0.03186	-0.04671	-0.17799	-0.1424	0.189733	0.58172	0.450014	0.096558	-0.267756
Mg	0.387578	-0.0215	0.171182	0.2886	0.197896	0.009612	-0.12795	0.141714	0.093627	0.34483	-0.07306	-0.12297	-0.40343	-0.18042	-0.17916	-0.534298
Mn	0.347034	0.102062	-0.2202	-0.26283	-0.18585	-0.18053	0.165913	0.196541	-0.19803	0.131209	-0.2683	0.422287	-0.0171	-0.32985	0.445898	-0.06032
Mo	-0.14962	-0.17747	0.141538	-0.158	0.188968	0.381233	0.578404	0.550142	0.07807	-0.05381	-0.20623	0.02832	0.021148	0.102812	-0.12322	-0.065871
Ni	-0.34487	-0.08695	-0.06714	0.475238	-0.01697	0.149643	-0.02508	0.068945	0.020447	0.008851	-0.14663	-0.25281	0.338189	-0.55513	0.317226	-0.077523
P	0.412348	-0.06718	0.231894	0.190199	0.096872	0.214679	-0.18408	0.157323	-0.00921	0.092818	-0.15696	-0.03621	-0.0305	-0.1051	-0.02042	0.757862
Rb	0.018962	-0.47707	0.268341	0.231725	-0.10633	-0.0047	0.203741	-0.2247	-0.29243	-0.32276	0.244558	0.41532	-0.17573	-0.26229	-0.14435	-0.022681
S	0.106035	0.4994	0.070597	0.178353	0.130711	-0.13017	0.216545	-0.09559	-0.105	-0.6327	-0.31626	-0.20395	-0.24073	-0.03839	0.011139	-0.000922
Sr	-0.0653	-0.46301	-0.34593	0.203957	0.259726	-0.23394	0.036937	0.060567	-0.01925	-0.04162	-0.05713	-0.14187	-0.38473	0.342445	0.43623	0.108625
Zn	-0.15451	-0.19067	0.248102	-0.07063	-0.44815	-0.12442	-0.45469	0.319023	0.328029	-0.31877	-0.3129	0.097251	-0.1399	0.061691	0.023572	-0.088738

FUGG

Element	aPC1	aPC2	aPC3	aPC4	aPC5	aPC6	aPC7	aPC8	aPC9	aPC10	aPC11	aPC12	aPC13	aPC14	aPC15	aPC16
As	0.006644	-0.42111	0.212756	-0.22598	0.30533	-0.16709	0.125187	-0.18334	0.216633	-0.47926	0.321313	-0.15097	0.006603	-0.27151	-0.27249	-0.072076
Ca	-0.23733	-0.06249	-0.56404	0.170993	0.224017	-0.18313	-0.07224	-0.14754	-0.15547	0.271959	0.489695	-0.25898	0.217695	-0.0764	0.14837	-0.03787
Cd	0.187674	0.3381	-0.31525	-0.06527	-0.05473	-0.06799	-0.04895	0.448003	0.412898	-0.4131	0.255379	0.148769	0.265246	0.185825	0.047239	-0.022293
Co	-0.16829	0.178419	-0.13319	-0.33124	0.252139	0.653431	0.45449	-0.04255	0.253393	0.187299	0.057151	-0.03931	-0.07077	-0.05969	0.026603	0.009439
Cu	-0.22185	0.400953	0.170752	0.136977	0.100522	-0.01402	0.033506	-0.02972	-0.11441	-0.12237	-0.23889	0.043627	0.496488	-0.5362	0.003219	-0.329754
Fe	0.265923	-0.18213	0.076771	0.221252	0.222497	0.085246	0.47287	0.47104	-0.45691	-0.07663	-0.02529	-0.24457	0.1858	0.169021	-0.01933	-0.021258
K	-0.19463	0.362582	0.104106	-0.25934	0.296416	-0.31895	0.031829	-0.0772	-0.11188	-0.19842	-0.1269	-0.3196	-0.30984	0.357249	0.315716	-0.250118
Mg	-0.33173	-0.06103	0.01201	-0.3965	-0.07132	-0.19705	-0.03017	0.462944	-0.12261	0.021133	-0.06557	-0.07322	-0.03393	-0.35536	0.180767	0.533369
Mn	0.215697	0.112322	-0.52824	-0.30311	-0.21838	0.039466	0.070964	-0.29155	-0.38506	-0.32444	-0.25491	-0.10298	-0.01177	-0.0834	-0.29149	0.09735
Mo	-0.35868	0.197193	0.148257	0.10213	0.145749	-0.094	0.063298	-0.16147	0.022407	-0.00101	-0.03424	0.006303	0.316377	0.423978	-0.48716	0.470106
Ni	0.246857	0.353186	-0.00449	0.14951	0.23406	-0.04242	-0.22896	0.242007	0.094365	0.22286	0.034478	-0.32625	-0.37766	-0.26981	-0.4916	0.032956
P	-0.34901	-0.11335	-0.02305	-0.31377	-0.21547	-0.03448	-0.03757	0.308025	-0.19187	0.174262	0.141967	0.177891	-0.03555	0.156556	-0.44252	-0.530916
Rb	0.237975	-0.05658	0.061218	-0.33187	0.530087	0.224779	-0.53942	0.014496	-0.2625	0.056401	-0.00688	0.265081	0.218037	0.09825	0.048755	0.040064
S	0.248104	0.288609	0.106494	-0.10366	0.075023	-0.35034	0.392142	-0.13564	-0.21273	0.180148	0.339141	0.543349	-0.14286	-0.11988	0.002178	0.098933
Sr	-0.21506	-0.21768	-0.38947	0.261706	0.422677	-0.15164	0.098147	0.12257	0.126895	-0.10317	-0.42462	0.422876	-0.25732	-0.043	-0.06645	-0.039074
Zn	-0.29977	0.143272	0.071343	0.31788	-0.04519	0.386381	-0.16199	0.03018	-0.34646	-0.44539	0.35706	0.155532	-0.34748	-0.04977	0.038309	0.100994

GRS

Element	aPC1	aPC2	aPC3	aPC4	aPC5	aPC6	aPC7	aPC8	aPC9	aPC10	aPC11	aPC12	aPC13	aPC14	aPC15	aPC16
As	0.314047	-0.14305	0.068601	-0.14893	0.066663	0.044829	0.186392	-0.28454	0.325777	-0.59309	-0.1659	0.337646	-0.30553	-0.16772	0.104481	-0.007302
Ca	0.2464	0.325775	-0.02055	0.049075	0.036592	-0.09137	0.060934	0.054581	0.085748	-0.10829	0.482603	-0.18191	0.077144	0.107806	0.715031	-0.005614
Cd	0.256306	-0.29791	0.01247	-0.01347	0.326357	-0.11116	-0.07154	0.235126	-0.16454	-0.12134	0.076345	0.049783	0.331847	-0.15213	-0.03899	0.693585
Co	0.243142	-0.32395	0.019096	-0.01663	0.271565	-0.11738	-0.03899	0.20791	-0.11634	-0.09883	0.011469	-0.00024	0.38867	-0.13778	-0.01153	-0.713726
Cu	0.263919	-0.28592	0.020226	0.061448	0.169069	-0.16038	-0.12081	0.226075	-0.22085	0.297651	0.101197	0.024024	-0.74941	0.080383	0.077315	-0.046513
Fe	0.326917	-0.12947	-0.00026	0.033484	0.01964	0.038249	0.189086	-0.01425	0.145318	0.026959	-0.26811	-0.13142	0.13606	0.837298	-0.07355	0.03801
K	0.242572	0.287736	-0.04812	0.061713	0.157748	-0.39464	-0.20005	-0.60676	-0.41658	0.047109	-0.27817	-0.07956	0.04678	-0.04093	0.010271	-0.001439
Mg	0.199425	0.377398	0.029885	0.024014	0.112269	-0.1917	-0.07097	0.090488	0.162628	-0.23741	0.450111	-0.11511	-0.10866	0.0705	-0.66037	-0.047119
Mn	0.097388	0.372832	-0.20751	0.077581	0.538748	0.658396	-0.12558	0.112298	-0.05987	0.032825	-0.18881	0.062206	-0.06464	-0.02436	0.009756	-0.035626
Mo	0.330365	-0.07249	0.02265	0.085666	-0.00199	0.058093	0.144013	-0.10942	0.467938	0.372675	-0.14591	-0.53583	0.002745	-0.41225	-0.05773	0.045976
Ni	-0.04794	-0.07298	-0.80243	0.478896	-0.10321	-0.20573	0.029527	0.11339	0.103327	-0.17186	-0.09537	0.013138	-0.03678	-0.03985	0.00096	0.003213
P	0.101424	0.415331	0.208604	-0.01861	-0.12226	-0.34522	-0.09291	0.552135	0.156878	0.011247	-0.50585	0.173483	0.004804	-0.0811	0.091241	0.009702
Rb	0.195412	0.018471	-0.47882	-0.78538	-0.22277	0.033405	-0.2279	0.062988	-0.01315	0.065355	0.00073	-0.06358	0.009508	0.001802	-0.0118	0.002366
S	0.323081	0.10528	-0.08855	0.090388	-0.1448	0.031686	0.237387	-0.11147	0.056984	0.48787	0.212609	0.669498	0.180326	-0.05581	-0.08556	-0.01125
Sr	0.302909	0.094831	0.037961	0.086447	-0.43187	0.262328	0.425429	0.169381	-0.55655	-0.20611	-0.05423	-0.19257	-0.04934	-0.14679	-0.09541	-0.000747
Zn	0.27419	-0.11607	0.145593	0.300256	-0.41779	0.273021	-0.72402	-0.05007	0.070918	-0.0986	0.037949	0.048918	0.069204	0.023896	0.016085	-0.00041

Supplementary Table 10. PC-QTLs identified in individual environment or tissue.

Locus symbol[1]	Environment/tissue	Principle component	Chromosome	Start (cM)	End (cM)	Physical position (Kb)	LOD	ADD[2]	PVE[3]
02GF_PC3	02GF	3	3	73.785	74.097	17130 ~ 17534	7.815	0.443	10.062
02GF_PC5	02GF	5	2	79.626	80.016	15571 ~ 16509	4.666	0.32	8.217
02GF_PC12	02GF	12	3	65.599	65.989	15009 ~ 15680	4.156	-0.166	8.385
02GF_PC14	02GF	14	2	114.022	114.411	23325 ~ 23873	4.022	-0.106	5.595
03GF_PC3	03GF	3	3	72.964	73.276	16895 ~ 17416	9.599	-0.503	11.761
03GF_PC5	03GF	5	2	70.149	70.542	9005 ~ 9751	4.117	0.341	9.711
03GF_PC9	03GF	9	1	66.528	66.92	13048 ~ 13304	4.29	0.179	6.457
03GF_PC10	03GF	10	5	68.654	69.044	15782 ~ 16219	4.03	-0.151	5.373
03GF_PC11	03GF	11	1	129.77	130.356	30534 ~ 31134	4.045	0.145	5.055
03GF_PC12	03GF	12	3	36.111	36.423	9597 ~ 9925	4.211	0.169	7.969
03GF_PC13	03GF	13	11	48.399	48.594	9759 ~ 10282	4.475	0.145	6.2
06GF_PC1	06GF	1	3	5.118	5.314	2211 ~ 2515	4.365	-0.682	12.666
06GF_PC2	06GF	2	1	28.704	29.094	4151 ~ 4469	4.694	0.64	12.587
06GF_PC5	06GF	5	3	67.38	67.575	15630 ~ 16243	4.013	-0.275	7.638
06GF_PC6	06GF	6	3	154.893	155.205	30365 ~ 30750	4.655	-0.337	13.431
06GF_PC7	06GF	7	2	70.149	70.737	9413 ~ 9751	8.445	0.34	15.457
06GF_PC8	06GF	8	8	37.197	37.705	3602 ~ 3939	5.05	0.253	9.791
06GF_PC9	06GF	9	9	16.757	16.757	7350 ~ 7583	5.04	-0.255	13.303
06GF_PC11	06GF	11	2	138.302	138.497	28585 ~ 29657	4.798	0.202	10.909
07GF_PC1	07GF	1	3	173.329	173.915	34655 ~ 35388	7.837	-0.765	18.063
07GF_PC5	07GF	5	10	92.626	92.938	17153 ~ 17433	8.244	0.516	20.981
07GF_PC6	07GF	6	5	97.401	97.714	21342 ~ 22849	5.476	0.311	9.82
07GF_PC7	07GF	7	3	0.39	0.39	475 ~ 751	6.202	0.339	13.895
07GF_PC9	07GF	9	3	107.393	107.393	24828 ~ 25054	4.243	0.223	8.473
07GF_PC16	07GF	16	3	65.599	65.989	15009 ~ 15680	4.191	-0.076	5.628
07GU_PC2	07GU	2	5	90.551	90.94	21015 ~ 21469	4.667	0.407	6.701
07GU_PC9	07GU	9	2	51.838	54.839	4987 ~ 5798	9.429	0.369	18.431
07GU_PC14	07GU	14	3	65.599	65.989	15009 ~ 15680	4.312	-0.16	9.585
08GF2_PC2	08GF2	2	2	106.137	106.529	21244 ~ 21828	7.665	-0.658	19.179

Locus symbol[1]	Environment/tissue	Principle component	Chromosome	Start (cM)	End (cM)	Physical position (Kb)	LOD	ADD[2]	PVE[3]
08GF2_PC3	08GF2	3	1	69.894	70.48	12887 ~ 14648	4.03	0.4	7.698
08GF2_PC4	08GF2	4	2	143.252	143.645	30663 ~ 30959	4.051	-0.442	12.787
08GF2_PC5	08GF2	5	3	25.115	25.31	6921 ~ 7503	4.714	-0.324	8.374
08GF2_PC9	08GF2	9	3	56.866	57.061	13858 ~ 14577	5.629	-0.255	10.55
08GF2_PC16	08GF2	16	1	47.206	47.596	8300 ~ 8726	4.819	-0.08	6.651
08GU1_PC2	08GU1	2	3	0.783	1.175	731 ~ 1013	4.915	-0.486	8.982
08GU1_PC3	08GU1	3	8	11.016	11.718	995 ~ 1387	6.477	-0.439	11.241
08GU1_PC9	08GU1	9	1	72.641	72.836	14367 ~ 20948	4.632	-0.243	8.371
08GU1_PC10	08GU1	10	2	51.838	54.839	4987 ~ 5798	4.407	-0.288	13.878
08GU1_PC12	08GU1	12	11	89.46	89.655	20598 ~ 20934	4.527	0.226	11.833
08GU1_PC15	08GU1	15	3	65.599	66.185	15009 ~ 15814	9.348	-0.189	19.523
08GU3_PC6	08GU3	6	3	31.925	32.71	8358 ~ 9374	6.188	0.354	10.399
08GU3_PC8	08GU3	8	6	78.825	78.825	7166 ~ 7413	4.697	0.341	15.074
08GU3_PC16	08GU3	16	1	48.182	48.767	7979 ~ 9014	4.372	-0.097	11.231
11GGH_PC7	11GGH	7	2	81.382	81.576	16555 ~ 16757	4.097	0.328	11.011
11GGH_PC8	11GGH	8	2	57.03	57.42	5672 ~ 5923	4.911	0.235	7.251
15RTH_PC4	15RTH	4	4	222.842	222.842	33160 ~ 33413	5.713	0.342	9.717
15RTH_PC5	15RTH	5	3	24.137	24.723	6638 ~ 6963	20.648	-0.597	34.862
15RTH_PC6	15RTH	6	6	18.602	18.602	1995 ~ 2216	4.18	0.337	12.2
15SHH_PC1	15SHH	1	11	83.595	83.79	19351 ~ 19879	7.404	-0.74	13.681
15SHH_PC2	15SHH	2	8	50.006	50.707	6478 ~ 7234	5.114	-0.471	8.761
15SHH_PC7	15SHH	7	6	32.627	32.939	3567 ~ 4015	5.137	0.293	10.335
15SHH_PC9	15SHH	9	7	51.187	51.382	7007 ~ 7904	4.157	0.228	7.693
15SHH_PC16	15SHH	16	7	52.367	52.756	8331 ~ 9044	8.247	-0.126	17.641

Locus symbol[1]	Environment/tissue	Principle component	Chromosome	Start (cM)	End (cM)	Physical position (Kb)	LOD	ADD[2]	PVE[3]

[1]: 02GF: grains (G) of RILs grown under flooded (F) on 2002;
03GF: grains (G) of RILs grown under flooded (F) on 2003;
06GF: grains (G) of RILs grown under flooded (F) on 2006;
07GF: grains (G) of RILs grown under flooded (F) on 2007;
07GU: grains (G) of RILs grown under unflooded (U) on 2007;
08GF2: grains (G) of RILs grown under flooded (F) in the field site number 2 on 2008;
08GU1: grains (G) of RILs grown under unflooded (U) in the field site number 1 on 2008;
08GU3: grains (G) of RILs grown under unflooded (U) in the field site number 3 on 2008;
11GGH: grains (G) of RILs grown in a greenhouse (GH) condition on 2011;
15RTH: roots (RT) of RILs grown hydroponically (H) on 2015;
15SHH: shoots (SH) of RILs grown hydroponically (H) on 2015.

[2]ADD: additive effect

[3]PVE: percentage of variance explained

Supplementary Table 11. aPC-QTLs identified across environments and tissues.

Locus symbol[1]	Across environment/tissue	Environment/ tissue	Principle component	Chromosome	Start (cM)	End (cM)	Physical position (Kb)	LOD	ADD[2]	PVE[3]
F_02GF_aPC2	F	02GF	2	11	12.889	13.084	2667 ~ 2976	5.929	0.389	11.084
F_02GF_aPC3	F	02GF	3	3	72.572	72.964	16795 ~ 17302	6.157	-0.43	10.993
F_02GF_aPC6	F	02GF	6	3	96.144	96.533	21250 ~ 21637	5.386	0.239	6.478
F_02GF_aPC8	F	02GF	8	2	77.235	77.627	11524 ~ 12290	5.412	-0.261	11.182
F_02GF_aPC12	F	02GF	12	3	65.599	66.185	15009 ~ 15814	6.413	-0.156	8.512
F_03GF_aPC1	F	03GF	1	3	107.393	107.393	24828 ~ 25054	4.357	0.213	4.395
F_03GF_aPC2	F	03GF	2	1	28.704	29.094	4151 ~ 4469	5.661	0.381	10.42
F_03GF_aPC3	F	03GF	3	3	65.599	65.989	15009 ~ 15680	14.571	-0.567	16.057
F_03GF_aPC3	F	03GF	3	8	40.695	41.084	4569 ~ 5234	8.334	0.465	10.813
F_03GF_aPC4	F	03GF	4	3	146.673	146.984	29007 ~ 29488	4.088	0.377	6.691
F_03GF_aPC5	F	03GF	5	2	133.887	134.082	27703 ~ 28099	5.415	0.229	7.308
F_03GF_aPC6	F	03GF	6	3	67.38	67.575	15630 ~ 16243	4.109	0.271	7.368
F_03GF_aPC8	F	03GF	8	11	14.279	14.279	2796 ~ 3031	5.204	-0.297	11.179
F_03GF_aPC12	F	03GF	12	3	65.599	65.989	15009 ~ 15680	10.207	-0.243	16.42
F_03GF_aPC16	F	03GF	16	5	73.667	73.667	17432 ~ 17675	17.478	0.073	5.965
F_06GF_aPC2	F	06GF	2	1	0	0.389	280 ~ 526	4.816	0.551	11.548
F_06GF_aPC2	F	06GF	2	11	0	0.392	553 ~ 755	5.401	0.597	13.597
F_06GF_aPC3	F	06GF	3	3	72.572	73.276	16795 ~ 17263	5.276	-0.428	9.632
F_06GF_aPC4	F	06GF	4	7	25.793	26.183	2692 ~ 3014	4.276	-0.312	5.404
F_06GF_aPC6	F	06GF	6	7	52.367	52.756	8331 ~ 9044	7.325	0.379	15.004
F_06GF_aPC10	F	06GF	10	2	63.981	64.371	7426 ~ 8314	6.522	0.213	8.345
F_06GF_aPC12	F	06GF	12	2	133.887	134.082	27703 ~ 28099	5.377	0.168	9.367
F_06GF_aPC13	F	06GF	13	3	79.525	79.836	19843 ~ 20700	5.535	0.141	6.866
F_07GF_aPC1	F	07GF	1	2	106.137	106.137	21244 ~ 21828	4.369	-0.285	11.291
F_07GF_aPC2	F	07GF	2	3	173.329	173.915	34655 ~ 35388	8.149	0.532	15.286
F_07GF_aPC12	F	07GF	12	6	113.886	114.198	18934 ~ 19410	4.365	-0.135	6.851
F_07GF_aPC13	F	07GF	13	3	102.242	102.634	23027 ~ 24369	6.255	0.17	11.188
F_07GF_aPC14	F	07GF	14	1	43.751	44.063	6487 ~ 7945	5.4	-0.133	8.183
F_07GF_aPC15	F	07GF	15	2	108.709	109.294	22317 ~ 22919	5.046	-0.12	5.534
F_07GF_aPC16	F	07GF	16	3	0.39	0.783	475 ~ 858	4.038	0.085	8.571
F_08GF2_aPC3	F	08GF2	3	2	108.904	109.294	22383 ~ 22919	7.859	0.446	14.444
F_08GF2_aPC5	F	08GF2	5	2	138.302	138.497	28585 ~ 29657	5.838	0.363	16.093
F_08GF2_aPC6	F	08GF2	6	10	108.35	108.74	19686 ~ 20840	6.814	-0.298	12.223

Locus symbol[1]	Across environment/tissue	Environment/ tissue	Principle component	Chromosome	Start (cM)	End (cM)	Physical position (Kb)	LOD	ADD[2]	PVE[3]
F_08GF2_aPC7	F	08GF2	7	3	159.147	159.537	30959 ~ 31392	5.148	0.224	11.708
F_08GF2_aPC9	F	08GF2	9	5	7.396	7.396	2294 ~ 2522	4.325	-0.25	13.655
F_08GF2_aPC10	F	08GF2	10	9	24.769	25.161	9295 ~ 9580	5.183	-0.223	9.416
F_08GF2_aPC11	F	08GF2	11	2	51.838	54.839	4987 ~ 5798	4.769	-0.223	10.719
F_08GF2_aPC15	F	08GF2	15	9	8.475	8.475	5700 ~ 5945	4.4	0.166	9.358
U_07GU_aPC6	U	07GU	6	2	51.838	54.839	4987 ~ 5798	5.277	0.281	9.568
U_07GU_aPC8	U	07GU	8	2	62.374	62.374	7398 ~ 7627	7.533	-0.299	12.302
U_07GU_aPC15	U	07GU	15	3	0.195	0.39	10 ~ 751	4.116	-0.076	3.126
U_08GU1_aPC2	U	08GU1	2	3	5.904	6.49	2150 ~ 2964	4.373	0.431	11.419
U_08GU1_aPC3	U	08GU1	3	12	21.601	21.796	7469 ~ 10488	4.943	0.316	6.585
U_08GU1_aPC6	U	08GU1	6	1	71.271	71.466	13480 ~ 19607	6.044	-0.352	10.036
U_08GU1_aPC13	U	08GU1	13	3	65.599	65.989	15009 ~ 15680	7.174	0.257	17.829
U_08GU1_aPC15	U	08GU1	15	3	65.599	66.185	15009 ~ 15814	6.998	-0.193	17.749
U_08GU3_aPC14	U	08GU3	14	2	133.887	134.082	27703 ~ 28099	5.257	-0.118	6.489
FUGG_02GF_aPC2	FUGG	02GF	2	3	72.964	73.276	16895 ~ 17416	5.274	0.17	9.987
FUGG_02GF_aPC3	FUGG	02GF	3	3	73.785	74.097	17130 ~ 17534	8.219	0.29	10.592
FUGG_02GF_aPC4	FUGG	02GF	4	3	67.38	67.575	15630 ~ 16243	5.33	-0.198	6.902
FUGG_02GF_aPC5	FUGG	02GF	5	3	72.572	72.964	16795 ~ 17302	5.001	-0.287	12.619
FUGG_02GF_aPC12	FUGG	02GF	12	3	65.599	65.794	15009 ~ 15553	6.007	-0.144	10.177
FUGG_03GF_aPC2	FUGG	03GF	2	3	67.38	67.575	15630 ~ 16243	4.437	0.172	8.189
FUGG_03GF_aPC3	FUGG	03GF	3	3	72.964	73.276	16895 ~ 17416	10.635	0.405	14.782
FUGG_03GF_aPC4	FUGG	03GF	4	2	89.172	89.561	18039 ~ 18477	5.183	0.232	7.524
FUGG_03GF_aPC4	FUGG	03GF	4	3	65.599	66.185	15009 ~ 15814	11.703	-0.326	14.854
FUGG_03GF_aPC5	FUGG	03GF	5	3	65.599	65.989	15009 ~ 15680	7.414	-0.277	17.571
FUGG_03GF_aPC6	FUGG	03GF	6	3	65.599	65.989	15009 ~ 15680	5.057	0.148	7.338
FUGG_03GF_aPC12	FUGG	03GF	12	3	65.599	65.794	15009 ~ 15553	7.388	-0.197	15.849
FUGG_03GF_aPC15	FUGG	03GF	15	7	90.678	90.678	16645 ~ 16866	5.125	-0.072	9.583
FUGG_06GF_aPC3	FUGG	06GF	3	3	74.606	74.996	17216 ~ 17853	4.569	0.255	6.856
FUGG_06GF_aPC4	FUGG	06GF	4	11	4.262	4.457	1104 ~ 1362	6.491	0.392	18.702
FUGG_06GF_aPC6	FUGG	06GF	6	2	80.016	80.211	16118 ~ 16564	5.227	0.198	11.585
FUGG_06GF_aPC7	FUGG	06GF	7	3	3.147	3.539	1510 ~ 2121	4.677	0.25	9.065
FUGG_06GF_aPC8	FUGG	06GF	8	3	173.329	173.915	34655 ~ 35388	5.484	-0.137	7.558
FUGG_06GF_aPC9	FUGG	06GF	9	5	4.214	4.214	1927 ~ 2149	4.942	-0.067	2.053
FUGG_06GF_aPC11	FUGG	06GF	11	3	65.599	65.794	15009 ~ 15553	5.126	0.15	8.265

Locus symbol[1]	Across environment/tissue	Environment/ tissue	Principle component	Chromosome	Start (cM)	End (cM)	Physical position (Kb)	LOD	ADD[2]	PVE[3]
FUGG_06GF_aPC12	FUGG	06GF	12	5	97.401	97.714	21342 ~ 22849	4.573	-0.145	10.991
FUGG_06GF_aPC13	FUGG	06GF	13	3	5.904	5.904	2150 ~ 2399	4.043	-0.129	10.083
FUGG_06GF_aPC15	FUGG	06GF	15	3	171.37	171.761	33876 ~ 34377	4.167	-0.119	13.676
FUGG_07GF_aPC3	FUGG	07GF	3	2	106.137	106.529	21244 ~ 21828	6.684	-0.306	9.751
FUGG_07GF_aPC4	FUGG	07GF	4	3	179.9	179.9	35596 ~ 35836	6.319	0.35	13.154
FUGG_07GF_aPC10	FUGG	07GF	10	10	93.641	93.952	17262 ~ 17851	6.381	-0.173	18.777
FUGG_07GF_aPC11	FUGG	07GF	11	8	35.397	35.591	3396 ~ 3608	5.676	-0.09	5.392
FUGG_07GF_aPC15	FUGG	07GF	15	7	82.128	82.323	10259 ~ 13394	5.062	-0.096	12.081
FUGG_07GU_aPC5	FUGG	07GU	5	6	0	0.39	19 ~ 201	4.051	-0.198	10.598
FUGG_07GU_aPC14	FUGG	07GU	14	2	51.838	54.839	4987 ~ 5798	10.418	0.227	26.14
FUGG_08GF2_aPC1	FUGG	08GF2	1	3	56.866	57.061	13858 ~ 14577	4.635	0.148	5.151
FUGG_08GF2_aPC3	FUGG	08GF2	3	3	72.572	73.276	16795 ~ 17263	7.637	0.276	12.384
FUGG_08GF2_aPC4	FUGG	08GF2	4	1	35.433	35.628	4441 ~ 5565	6.068	0.28	11.91
FUGG_08GF2_aPC7	FUGG	08GF2	7	2	143.252	143.645	30663 ~ 30959	6.287	-0.23	14.846
FUGG_08GF2_aPC10	FUGG	08GF2	10	2	85.093	85.288	17512 ~ 17936	4.339	-0.189	14.813
FUGG_08GF2_aPC12	FUGG	08GF2	12	5	0	0.312	26 ~ 183	4.309	-0.151	10.632
FUGG_08GF2_aPC14	FUGG	08GF2	14	3	98.129	98.519	22755 ~ 23002	4.889	0.098	10.178
FUGG_08GF2_aPC15	FUGG	08GF2	15	3	171.37	171.761	33876 ~ 34377	5.156	-0.122	16.307
FUGG_08GF2_aPC15	FUGG	08GF2	15	7	92.078	92.078	16902 ~ 17141	4.43	-0.115	14.652
FUGG_08GF2_aPC16	FUGG	08GF2	16	11	33.143	33.533	7029 ~ 7296	6.139	-0.08	9.024
FUGG_08GU1_aPC8	FUGG	08GU1	8	5	7.396	7.591	2294 ~ 3528	4.79	0.223	9.428
FUGG_08GU1_aPC11	FUGG	08GU1	11	3	65.599	65.989	15009 ~ 15680	4.901	0.124	7.364
FUGG_08GU1_aPC12	FUGG	08GU1	12	3	67.38	67.575	15630 ~ 16243	5.521	-0.213	15.972
FUGG_08GU3_aPC12	FUGG	08GU3	12	1	221.474	221.863	42743 ~ 43112	6.01	-0.278	24.819
FUGG_08GU3_aPC15	FUGG	08GU3	15	2	79.626	80.016	15571 ~ 16509	5.152	-0.163	14.83
FUGG_08GU3_aPC15	FUGG	08GU3	15	7	39.557	39.869	5035 ~ 5303	6.309	-0.175	17.06
FUGG_11GGH_aPC2	FUGG	11GGH	2	2	51.838	54.839	4987 ~ 5798	4.465	-0.237	12.032
FUGG_11GGH_aPC12	FUGG	11GGH	12	8	22.41	23.312	2479 ~ 2754	6.005	-0.084	3.01
FUGG_11GGH_aPC13	FUGG	11GGH	13	8	1.793	1.988	40 ~ 737	5.718	0.17	7.132
FUGG_11GGH_aPC14	FUGG	11GGH	14	2	51.838	54.839	4987 ~ 5798	8.38	0.275	15.838
FUGG_03GF_aPC11	FUGG5	03GF	11	1	34.052	34.247	4924 ~ 5443	4.187	-0.127	6.927
GRS_02GF_aPC2	GRS	02GF	2	11	14.279	14.279	2796 ~ 3031	4.059	-0.041	4.875
GRS_02GF_aPC3	GRS	02GF	3	3	1.965	2.161	757 ~ 1496	8.548	0.118	15.933
GRS_02GF_aPC4	GRS	02GF	4	3	0.783	1.175	731 ~ 1013	8.541	0.198	14.845

Locus symbol[1]	Across environment/tissue	Environment/ tissue	Principle component	Chromosome	Start (cM)	End (cM)	Physical position (Kb)	LOD	ADD[2]	PVE[3]
GRS_02GF_aPC6	GRS	02GF	6	2	92.161	92.473	18706 ~ 19096	4.628	0.055	7.373
GRS_02GF_aPC9	GRS	02GF	9	3	65.599	66.185	15009 ~ 15814	5.804	0.027	11.922
GRS_03GF_aPC1	GRS	03GF	1	7	93.061	93.451	17092 ~ 17521	4.389	-0.037	10.981
GRS_03GF_aPC2	GRS	03GF	2	11	14.279	14.279	2796 ~ 3031	4.421	-0.053	9.077
GRS_03GF_aPC4	GRS	03GF	4	9	95.087	95.283	22149 ~ 22591	6.096	0.121	11.642
GRS_03GF_aPC5	GRS	03GF	5	11	14.279	14.279	2796 ~ 3031	4.989	-0.058	9.437
GRS_03GF_aPC6	GRS	03GF	6	3	72.964	73.276	16895 ~ 17416	5.635	-0.061	6.911
GRS_03GF_aPC7	GRS	03GF	7	6	80.119	80.821	7576 ~ 7991	5.019	-0.057	8.727
GRS_03GF_aPC9	GRS	03GF	9	3	65.599	65.989	15009 ~ 15680	12.728	0.043	20.666
GRS_03GF_aPC12	GRS	03GF	12	3	72.964	73.276	16895 ~ 17416	5.419	0.021	6.946
GRS_06GF_aPC2	GRS	06GF	2	11	17.871	18.263	3588 ~ 3894	4.803	-0.067	11.445
GRS_06GF_aPC3	GRS	06GF	3	3	5.904	6.49	2150 ~ 2964	6.931	0.22	20.102
GRS_06GF_aPC4	GRS	06GF	4	3	5.904	6.1	2150 ~ 2568	6.053	0.394	20.394
GRS_06GF_aPC5	GRS	06GF	5	7	30.515	30.515	3722 ~ 3958	6.313	0.11	13.87
GRS_06GF_aPC6	GRS	06GF	6	7	52.367	52.756	8331 ~ 9044	6.841	0.092	18.039
GRS_06GF_aPC12	GRS	06GF	12	3	75.892	76.203	17548 ~ 17953	4.553	0.027	8.886
GRS_07GF_aPC1	GRS	07GF	1	3	171.956	172.348	33992 ~ 34411	4.169	-0.033	6.394
GRS_07GF_aPC3	GRS	07GF	3	3	0	0.39	82 ~ 751	7.064	0.085	13.562
GRS_07GF_aPC5	GRS	07GF	5	7	83.113	83.308	14625 ~ 14955	4.902	0.051	10.381
GRS_07GF_aPC7	GRS	07GF	7	3	2.951	3.147	1477 ~ 1733	8.96	0.057	7.293
GRS_07GF_aPC9	GRS	07GF	9	3	67.38	67.575	15630 ~ 16243	7.047	0.03	14.271
GRS_07GF_aPC9	GRS	07GF	9	10	93.641	93.952	17262 ~ 17851	6.047	0.031	15.018
GRS_07GF_aPC10	GRS	07GF	10	3	5.904	5.904	2150 ~ 2399	4.436	-0.044	12.484
GRS_07GF_aPC12	GRS	07GF	12	3	98.129	98.519	22755 ~ 23002	6.605	0.033	17.335
GRS_07GU_aPC13	GRS	07GU	13	2	51.838	54.839	4987 ~ 5798	12.019	0.035	21.728
GRS_07GU_aPC14	GRS	07GU	14	3	1.965	2.161	757 ~ 1496	6.974	0.023	20.27
GRS_07GU_aPC15	GRS	07GU	15	2	89.172	89.561	18039 ~ 18477	4.609	0.019	10.816
GRS_07GU_aPC15	GRS	07GU	15	5	90.551	90.94	21015 ~ 21469	4.518	0.018	10.023
GRS_08GF2_aPC3	GRS	08GF2	3	3	2.951	3.147	1477 ~ 1733	7.242	0.09	9.639
GRS_08GF2_aPC5	GRS	08GF2	5	7	55.531	55.531	9360 ~ 9598	7.266	0.053	10.94
GRS_08GF2_aPC6	GRS	08GF2	6	2	106.137	106.529	21244 ~ 21828	6.793	0.089	18.008
GRS_08GF2_aPC8	GRS	08GF2	8	2	143.252	143.645	30663 ~ 30959	4.49	0.033	7.698
GRS_08GF2_aPC16	GRS	08GF2	16	7	63.245	63.634	12866 ~ 13067	4.484	-0.002	8.318
GRS_08GU1_aPC1	GRS	08GU1	1	3	0.783	1.175	731 ~ 1013	6.498	-0.1	20.945

Locus symbol[1]	Across environment/tissue	Environment/ tissue	Principle component	Chromosome	Start (cM)	End (cM)	Physical position (Kb)	LOD	ADD[2]	PVE[3]
GRS_08GU1_aPC2	GRS	08GU1	2	2	92.161	92.473	18706 ~ 19096	5.517	-0.083	12.948
GRS_08GU1_aPC3	GRS	08GU1	3	3	0.783	1.175	731 ~ 1013	6.802	0.287	12.286
GRS_08GU1_aPC4	GRS	08GU1	4	3	5.904	6.295	2150 ~ 2742	7.738	0.354	22.133
GRS_08GU1_aPC5	GRS	08GU1	5	3	0.783	1.175	731 ~ 1013	10.704	0.144	18.426
GRS_08GU1_aPC5	GRS	08GU1	5	7	51.187	51.382	7007 ~ 7904	7.341	0.116	12.01
GRS_08GU1_aPC6	GRS	08GU1	6	4	219.8	220.191	33503 ~ 34084	4.187	0.097	11.96
GRS_08GU1_aPC7	GRS	08GU1	7	3	0.783	1.175	731 ~ 1013	7.132	0.131	16.972
GRS_08GU1_aPC13	GRS	08GU1	13	2	57.42	57.42	5698 ~ 5923	3.931	0.016	7.103
GRS_11GGH_aPC8	GRS	11GGH	8	9	24.769	25.552	9295 ~ 9869	4.977	0.078	9.018
GRS_11GGH_aPC11	GRS	11GGH	11	8	37.197	37.509	3602 ~ 3876	4.361	0.06	7.278
GRS_15RTH_aPC4	GRS	15RTH	4	2	109.099	109.294	22418 ~ 22919	6.019	0.206	9.843
GRS_15RTH_aPC11	GRS	15RTH	11	12	0	0.393	54 ~ 1267	4.524	-0.152	11.26
GRS_15RTH_aPC13	GRS	15RTH	13	11	63.02	63.721	16254 ~ 16666	4.545	0.134	6.836
GRS_15SHH_aPC2	GRS	15SHH	2	3	27.126	27.126	7302 ~ 7527	6.753	0.16	12.38
GRS_15SHH_aPC5	GRS	15SHH	5	11	83.595	83.79	19351 ~ 19879	4.337	0.199	7.551
GRS_15SHH_aPC6	GRS	15SHH	6	11	83.595	83.79	19351 ~ 19879	7.16	0.371	13.466
GRS_15SHH_aPC13	GRS	15SHH	13	7	33.839	34.035	4014 ~ 4330	5.133	-0.047	8.55

[1]: 02GF: grains (G) of RILs grown under flooded (F) on 2002;
03GF: grains (G) of RILs grown under flooded (F) on 2003;
06GF: grains (G) of RILs grown under flooded (F) on 2006;
07GF: grains (G) of RILs grown under flooded (F) on 2007;
07GU: grains (G) of RILs grown under unflooded (U) on 2007;
08GF2: grains (G) of RILs grown under flooded (F) in the field site number 2 on 2008;
08GU1: grains (G) of RILs grown under unflooded (U) in the field site number 1 on 2008;
08GU3: grains (G) of RILs grown under unflooded (U) in the field site number 3 on 2008;
11GGH: grains (G) of RILs grown in a greenhouse (GH) condition on 2011;
15RTH: roots (RT) of RILs grown hydroponically (H) on 2015;
15SHH: shoots (SH) of RILs grown hydroponically (H) on 2015;
F: 02GF+03GF+06GF+07GF+08GF2;
U: 07GU+08GU1+08GU3;
FUGG: F+U+11GGH;
GRS: FUGG+15RTH+15SHH.

[2]ADD: additive effect

[3]PVE: percentage of variance explained

Supplementary Table 12. Co-localization of elemental QTLs with PC-QTLs and aPC-QTLs. The QTLs in color background were the elemental QTLs co-localized with at least one PC-QTL or aPC-QTLs.

Locus symbol[1]	Across environment/tissue	Environment/tissue	Principle component/tissue	Chromosome	Start (cM)	End (cM)	Physical position (Kb)	LOD	ADD[2]	PVE[3]	Group	Elements with top 5 PC loadings[4]	Candidate gene	
													ID	Annotation
F_06GF_aPC2	F	06GF	aPC2	1	0	0.389	280 ~ 526	4.816	0.551	11.548	aPC-QTL		LOC_Os01g01610	IscA-like iron-sulfur assembly protein mitochondrial precursor
P		03GF	Grain	1	28.704	29.094	4151 ~ 4469	6.099	-77.026	7.744	Elemental QTL		LOC_Os01g08660	Aquaporin protein (SIP1;1)
06GF_PC2		06GF	PC2	1	28.704	29.094	4151 ~ 4469	4.694	0.64	12.587	PC-QTL	P Mg Fe K S		
F_03GF_aPC2	F	03GF	aPC2	1	28.704	29.094	4151 ~ 4469	5.661	0.381	10.42	aPC-QTL	Mg Cu P As Cd		
Co		08GU1	Grain	1	34.052	34.247	4924 ~ 5443	3.331	-0.012	7.298	Elemental QTL		LOC_Os01g09730	TNFR/CD27/30/40/95 cysteine-rich region domain containing protein
Co		U_mean	Grain	1	34.052	34.247	4924 ~ 5443	7.485	-0.005	12.536	Elemental QTL		LOC_Os01g09790	IQ calmodulin-binding motif domain containing protein
Co		02-06_mean	Grain	1	34.052	34.247	4924 ~ 5443	3.704	-0.004	5.958	Elemental QTL		LOC_Os01g09830	OsGrx_A2 - glutaredoxin subgroup III
FUGG_03GF_aPC11	FUGG	03GF	aPC11	1	34.052	34.247	4924 ~ 5443	4.187	-0.127	6.927	aPC-QTL	Ca Sr Zn S As		
K		08GF2	Grain	1	35.433	35.628	4441 ~ 5565	5.156	-129.614	13.164	Elemental QTL		LOC_Os01g09470	IQ calmodulin-binding motif family protein
K		F_mean	Grain	1	35.433	35.628	4441 ~ 5565	5.072	-88.211	13.89	Elemental QTL		LOC_Os01g09790	IQ calmodulin-binding motif domain containing protein
K		U_mean	Grain	1	35.433	35.628	4441 ~ 5565	4.481	-107.642	14.757	Elemental QTL			
K		02-06_mean	Grain	1	35.433	35.628	4441 ~ 5565	6.634	-92.566	13.722	Elemental QTL			
FUGG_08GF2_aPC4	FUGG	08GF2	aPC4	1	35.433	35.628	4441 ~ 5565	6.068	0.28	11.91	aPC-QTL	Mg Rb Co Zn P		
Co		08GF2	Grain	1	35.628	36.212	4411 ~ 5699	5.674	-0.004	11.776	Elemental QTL		LOC_Os01g10400	Similar to Metallothionein-like protein type 3 (MT-3) (MWMT3)
P		U_mean	Grain	1	35.628	36.212	4411 ~ 5699	3.854	-63.955	6.324	Elemental QTL		LOC_Os01g09830	OsGrx_A2 - glutaredoxin subgroup III
Co		03GF	Grain	1	40.72	40.72	7268 ~ 7497	4.303	-0.004	4.71	Elemental QTL		LOC_Os01g13120	aquaporin protein
													LOC_Os01g13130	aquaporin protein
K		06GF	Grain	1	40.72	40.72	7268 ~ 7497	3.743	-102.376	14.403	Elemental QTL		LOC_Os01g13120	aquaporin protein
													LOC_Os01g13130	aquaporin protein
K		07GF	Grain	1	43.751	44.063	6487 ~ 7945	6.75	-95.89	14.003	Elemental QTL		LOC_Os01g11946	ABC transporter ATP-binding protein
K		08GF2	Grain	1	43.751	43.751	6487 ~ 6735	3.196	-106.079	8.823	Elemental QTL		LOC_Os01g12210	aluminum-activated malate transporter
F_07GF_aPC14	F	07GF	aPC14	1	43.751	44.063	6487 ~ 7945	5.4	-0.133	8.183	aPC-QTL	Mg Ca Ni Cu S		
08GF2_PC16		08GF2	PC16	1	47.206	47.596	8300 ~ 8726	4.819	-0.08	6.651	PC-QTL		LOC_Os01g15010	Similar to NTGB2
Co		11GGH	Grain	1	48.182	48.572	8813 ~ 9111	5.006	-0.008	6.734	Elemental QTL		LOC_Os01g14700	Heavy metal-associated domain HMA domain containing protein
08GU3_PC16		08GU3	PC16	1	48.182	48.767	7979 ~ 9014	4.372	-0.097	11.231	PC-QTL	P Mg Sr Ca Co		
K		02GF	Grain	1	63.879	64.191	12322 ~ 12743	4.945	-74.328	8.619	Elemental QTL		LOC_Os01g22700	organic cation transporter-related putative expressed
03GF_PC9		03GF	PC9	1	66.528	66.92	13048 ~ 13304	4.29	0.179	6.457	PC-QTL		LOC_Os01g23620	Similar to GTP-binding protein SAR1A
Rb		15RTH	Root	1	67.711	67.711	12414 ~ 12650	4.142	-0.821	9.499	Elemental QTL		LOC_Os01g22390	WD domain containing protein putative expressed
08GF2_PC3		08GF2	PC3	1	69.894	70.48	12887 ~ 14648	4.03	0.4	7.698	PC-QTL		LOC_Os01g25386	Similar to multidrug resistance protein ABC transporter family
U_08GU1_aPC6	U	08GU1	aPC6	1	71.271	71.466	13480 ~ 19607	6.044	-0.352	10.036	aPC-QTL		LOC_Os01g33240	ABC transporter-like domain containing protein
08GU1_PC9		08GU1	PC9	1	72.641	72.836	14367 ~ 20948	4.632	-0.243	8.371	PC-QTL		LOC_Os01g34850	Similar to HKT1 (High-affinity potassium uptake transporter)
K		11GGH	Grain	1	119.806	120.196	27893 ~ 28357	3.925	-138.792	7.972	Elemental QTL		LOC_Os01g48680	two pore calcium channel protein 1
K		03GF	Grain	1	123.474	123.866	28388 ~ 29992	3.832	-79.53	10.886	Elemental QTL		LOC_Os01g50100	ABC transporter ATP-binding protein
P		08GF2	Grain	1	123.474	123.866	28388 ~ 29992	4.728	-73.893	6.734	Elemental QTL		LOC_Os01g50160	MDR-like ABC transporter
P		08GF2	Grain	1	125.846	125.846	28836 ~ 29068	3.18	-44.273	2.418	Elemental QTL		LOC_Os01g50460	MtN3 and saliva related transmembrane protein family protein (SWEET2B)
K		06GF	Grain	1	129.77	129.77	30534 ~ 30758	3.238	-92.426	11.739	Elemental QTL		LOC_Os01g53570	aluminum-activated malate transporter
03GF_PC11		03GF	PC11	1	129.77	130.356	30534 ~ 31134	4.045	0.145	5.055	PC-QTL	Ni K Cd Mg S		
Ni		15RTH	Root	1	211.996	212.385	42038 ~ 42512	4.024	0.2	5.643	Elemental QTL		LOC_Os01g72570	CRT-like transporter Glutathione homeostasis Arsenic tolerance
FUGG_08GU3_aPC12	FUGG	08GU3	aPC12	1	221.474	221.863	42743 ~ 43112	6.01	-0.278	24.819	aPC-QTL		LOC_Os01g73530	ABC transporter ATP-binding protein
As		08GF2	Grain	2	51.838	54.839	4987 ~ 5798	3.098	0.059	5.02	Elemental QTL		LOC_Os02g10290	Heavy metal-transporting P1B type ATPase (OsHMA4)
Cu		07GF	Grain	2	51.838	54.839	4987 ~ 5798	9.111	-0.206	10.123	Elemental QTL			
Cu		08GF2	Grain	2	51.838	54.839	4987 ~ 5798	9.043	-0.313	15.664	Elemental QTL			
Cu		08GU1	Grain	2	51.838	54.839	4987 ~ 5798	7.16	-0.435	12.137	Elemental QTL			
Cu		07GU	Grain	2	51.838	54.839	4987 ~ 5798	14.342	-0.807	26.35	Elemental QTL			
Cu		F_mean	Grain	2	51.838	54.839	4987 ~ 5798	10.043	-0.203	13.364	Elemental QTL			
Cu		U_mean	Grain	2	51.838	54.839	4987 ~ 5798	13.879	-0.252	15.698	Elemental QTL			
Cu		02-06_mean	Grain	2	51.838	54.839	4987 ~ 5798	14.398	-0.318	20.196	Elemental QTL			
07GU_PC9		07GU	PC9	2	51.838	54.839	4987 ~ 5798	9.429	0.369	18.431	PC-QTL	Cu Ni Fe As Cd		
08GU1_PC10		08GU1	PC10	2	51.838	54.839	4987 ~ 5798	4.407	-0.288	13.878	PC-QTL	As Fe S Ni Zn		
FUGG_11GGH_aPC2	FUGG	11GGH	aPC2	2	51.838	54.839	4987 ~ 5798	4.465	-0.237	12.032	aPC-QTL	As Cu K Ni Cd		
U_07GU_aPC6	U	07GU	aPC6	2	51.838	54.839	4987 ~ 5798	5.277	0.281	9.568	aPC-QTL	Cu Fe Mo As Co		
F_08GF2_aPC11	F	08GF2	aPC11	2	51.838	54.839	4987 ~ 5798	4.769	-0.223	10.719	aPC-QTL	Cd As Mn Zn Rb		
GRS_07GU_aPC13	GRS[7]	07GU	aPC13	2	51.838	54.839	4987 ~ 5798	12.019	0.035	21.728	aPC-QTL	Cu Co Cd As S		
FUGG_07GU_aPC14	FUGG	07GU	aPC14	2	51.838	54.839	4987 ~ 5798	10.418	0.227	26.14	aPC-QTL	Cu Mo K Mg As		
FUGG_11GGH_aPC14	FUGG	11GGH	aPC14	2	51.838	54.839	4987 ~ 5798	8.38	0.275	15.838	aPC-QTL	Cu Mo K Mg As		

Locus symbol[1]	Across environment/tissue	Environment/tissue	Principle component/tissue	Chromosome	Start (cM)	End (cM)	Physical position (Kb)	LOD	ADD[2]	PVE[3]	Group	Elements with top 5 PC loadings[4]	Candidate gene ID	Candidate gene Annotation
Cd		U_mean	Grain	2	57.03	57.42	5672 ~ 5923	3.192	0.001	2.936	Elemental QTL		LOC_Os02g10800	ADP-glucose transporter
11GGH_PC8		11GGH	PC8	2	57.03	57.42	5672 ~ 5923	4.911	0.235	7.251	PC-QTL	Cu Co **Cd** K Mo		
GRS_08GU1_aPC13	GRS	08GU1	aPC13	2	57.42	57.42	5698 ~ 5923	3.931	0.016	7.103	aPC-QTL		LOC_Os02g10800	ADP-glucose transporter
U_07GU_aPC8	U	07GU	aPC8	2	62.374	62.374	7398 ~ 7627	7.533	-0.299	12.302	aPC-QTL		LOC_Os02g13870	A member of the Nodulin26-like Intrinsic Protein (NIP) family
Fe		03GF	Grain	2	63.981	64.176	7426 ~ 8010	3.518	0.379	5.276	Elemental QTL		LOC_Os02g13870	A member of the Nodulin26-like Intrinsic Protein (NIP) family
F_06GF_aPC10	F	06GF	aPC10	2	63.981	64.371	7426 ~ 8314	6.522	0.213	8.345	aPC-QTL	Cu Zn S **Fe** Mo	LOC_Os02g14840	potassium channel KAT1
Cd		07GU	Grain	2	64.764	64.764	8194 ~ 8419	5.27	0.001	0.118	Elemental QTL		LOC_Os02g14840	potassium channel KAT1
Fe		F_mean	Grain	2	65.156	65.351	8184 ~ 8521	4.752	0.432	10.775	Elemental QTL		LOC_Os02g14840	potassium channel KAT1
03GF_PC5		03GF	PC5	2	70.149	70.542	9005 ~ 9751	4.117	0.341	9.711	PC-QTL	Mo Zn **Fe** Mn Sr	LOC_Os02g16660	Similar to Transient receptor potential cation channel subfamily A member 1
06GF_PC7		06GF	PC7	2	70.149	70.737	9413 ~ 9751	8.445	0.34	15.457	PC-QTL	Co Cu Mn **Fe** Zn		
Fe		08GF2	Grain	2	70.542	71.129	8983 ~ 9931	6.221	0.478	11.919	Elemental QTL			
Fe		U_mean	Grain	2	70.542	71.129	8983 ~ 9931	4.054	0.583	7.404	Elemental QTL			
Co		02GF	Grain	2	74.093	74.288	10459 ~ 10672	4.364	0.004	7.993	Elemental QTL		LOC_Os02g18180	ATP-binding cassette sub-family E member 1
Fe		08GF2	Grain	2	74.093	74.288	10459 ~ 10672	4.369	0.397	8.028	Elemental QTL		LOC_Os02g18180	ATP-binding cassette sub-family E member 1
F_02GF_aPC8	F	02GF	aPC8	2	77.235	77.627	11524 ~ 12290	5.412	-0.261	11.182	aPC-QTL		LOC_Os02g20330	Similar to cation cation antiporter
S		08GU3	Grain	2	78.919	80.211	15471 ~ 16319	10.803	-101.379	3.193	Elemental QTL		LOC_Os02g27490	Bile acid:sodium symporter family protein
Mg		08GU3	Grain	2	79.626	80.016	15571 ~ 16509	7.604	-20.056	1.448	Elemental QTL		LOC_Os02g26700	Cation transport regulator-like protein (OsARP)
02GF_PC5		02GF	PC5	2	79.626	80.016	15571 ~ 16509	4.666	0.32	8.217	PC-QTL	Mo Zn Mn Fe Rb		
FUGG_08GU3_aPC15	FUGG	08GU3	aPC15	2	79.626	80.016	15571 ~ 16509	5.152	-0.163	14.83	aPC-QTL	Ni Mo P K Mn		
Fe		02-06_mean	Grain	2	80.016	80.992	16363 ~ 16704	24.327	0.713	2.15	Elemental QTL		LOC_Os02g27490	Bile acid:sodium symporter family protein
FUGG_06GF_aPC6	FUGG	06GF	aPC6	2	80.016	80.211	16118 ~ 16564	5.227	0.198	11.585	aPC-QTL	Co Zn S K Rb		
11GGH_PC7		11GGH	PC7	2	81.382	81.576	16555 ~ 16757	4.097	0.328	11.011	PC-QTL			
Zn		02GF	Grain	2	85.093	85.288	17512 ~ 17936	4.797	1.14	11.163	Elemental QTL		LOC_Os02g29510	Similar to non-imprinted in Prader-Willi/Angelman syndrome region protein 1
FUGG_08GF2_aPC10	FUGG	08GF2	aPC10	2	85.093	85.288	17512 ~ 17936	4.339	-0.189	14.813	aPC-QTL	As **Zn** Cd Mn Ca		
Mg		07GU	Grain	2	89.172	89.561	18039 ~ 18477	3.195	-39.702	8.57	Elemental QTL		LOC_Os02g30650	heavy metal-associated domain containing protein
FUGG_03GF_aPC4	FUGG	03GF	aPC4	2	89.172	89.561	18039 ~ 18477	5.183	0.232	7.524	aPC-QTL	**Mg** Rb Co Zn P		
GRS_07GU_aPC15	GRS	07GU	aPC15	2	89.172	89.561	18039 ~ 18477	4.609	0.019	10.816	aPC-QTL		LOC_Os02g30650	heavy metal-associated domain containing protein
P		08GU1	Grain	2	92.161	92.473	18706 ~ 19096	3.57	-102.2	7.777	Elemental QTL		LOC_Os02g31867	C2 calcium-dependent membrane targeting domain containing protein
GRS_08GU1_aPC2	GRS	08GU1	aPC2	2	92.161	92.473	18706 ~ 19096	5.517	-0.083	12.948	aPC-QTL	**P** Mg Mn Ca Co	LOC_Os02g31910	potassium transporter
GRS_02GF_aPC6	GRS	02GF	aPC6	2	92.161	92.473	18706 ~ 19096	4.628	0.055	7.373	aPC-QTL	Mn K **P** Zn Sr	LOC_Os02g31940	potassium transporter
Zn		U_mean	Grain	2	100.645	101.428	20098 ~ 20585	5.015	0.972	12.163	Elemental QTL		LOC_Os02g33820	abscisic stress-ripening
Zn		07GF	Grain	2	101.037	101.233	20274 ~ 21435	4.051	1.086	10.23	Elemental QTL		LOC_Os02g34580	ammonium transporter protein
Zn		08GF2	Grain	2	101.037	101.233	20274 ~ 21435	5.918	0.953	10.67	Elemental QTL		LOC_Os02g35190	chloride channel protein
Ca		06GF	Grain	2	106.137	106.529	21244 ~ 21828	4.896	5.005	6.411	Elemental QTL		LOC_Os02g36414	Transporter family protein
08GF2_PC2		08GF2	PC2	2	106.137	106.529	21244 ~ 21828	7.665	-0.658	19.179	PC-QTL	Sr **Ca** Mn P K	LOC_Os02g36440	Transporter family protein
F_07GF_aPC1	F	07GF	aPC1	2	106.137	106.137	21244 ~ 21828	4.369	-0.285	11.291	aPC-QTL	Ni S As P K	LOC_Os02g36450	Transporter family protein
FUGG_07GF_aPC3	FUGG	07GF	aPC3	2	106.137	106.529	21244 ~ 21828	6.684	-0.306	9.751	aPC-QTL	**Ca** Mn Sr Cd As		
GRS_08GF2_aPC6	GRS	08GF2	aPC6	2	106.137	106.529	21244 ~ 21828	6.793	0.089	18.008	aPC-QTL	Mn K P Zn Sr		
F_07GF_aPC15	F	07GF	aPC15	2	108.709	109.294	22317 ~ 22919	5.046	-0.12	5.534	aPC-QTL		LOC_Os02g37160	heavy metal associated domain containing protein
F_08GF2_aPC3	F	08GF2	aPC3	2	108.904	109.294	22383 ~ 22919	7.859	0.446	14.444	aPC-QTL		LOC_Os02g37280	heavy metal associated domain containing protein
Ca		02-06_mean	Grain	2	109.099	109.294	22418 ~ 22919	6.957	4.44	5.632	Elemental QTL		LOC_Os02g37094	Similar to ABC-2 type transporter family protein
GRS_15RTH_aPC4	GRS	15RTH	aPC4	2	109.099	109.294	22418 ~ 22919	6.019	0.206	9.843	aPC-QTL	Rb Ni Zn As S	LOC_Os02g37160	heavy metal transport Fdetoxification protein
Ca		U_mean	Grain	2	114.022	114.411	23325 ~ 23873	9.323	7.02	9.651	Elemental QTL		LOC_Os02g38750	Similar to Iron-stress related protein
02GF_PC14		02GF	PC14	2	114.022	114.411	23325 ~ 23873	4.022	-0.106	5.595	PC-QTL	S Fe Ni As P	LOC_Os02g39380	OsCML17 - Calmodulin-related calcium sensor protein
													LOC_Os02g39550	calcium-binding mitochondrial protein anon-60Da
Sr		07GF	Grain	2	119.339	120.04	24308 ~ 24723	7.951	0.041	9.976	Elemental QTL		LOC_Os02g40710	ammonium transporter protein
Mn		08GF2	Grain	2	130.814	131.126	26561 ~ 26832	3.426	1.963	11.489	Elemental QTL		LOC_Os02g44080	aquaporin protein
F_03GF_aPC5	F	03GF	aPC5	2	133.887	134.082	27703 ~ 28099	5.415	0.229	7.308	aPC-QTL		LOC_Os02g45690	Mechanosensitive ion channel MscS domain containing protein
F_06GF_aPC12	F	06GF	aPC12	2	133.887	134.082	27703 ~ 28099	5.377	0.168	9.367	aPC-QTL		LOC_Os02g45690	Mechanosensitive ion channel MscS domain containing protein
U_08GU3_aPC14	U	08GU3	aPC14	2	133.887	134.082	27703 ~ 28099	5.257	-0.118	6.489	aPC-QTL		LOC_Os02g45690	Mechanosensitive ion channel MscS domain containing protein
Cd		02GF	Grain	2	138.302	138.497	28585 ~ 29657	3.627	-0.002	6.212	Elemental QTL		LOC_Os02g46990	AAA-type ATPase family protein
Cd		F_mean	Grain	2	138.302	138.497	28585 ~ 29657	5.122	-0.002	6.294	Elemental QTL			
06GF_PC11		06GF	PC11	2	138.302	138.497	28585 ~ 29657	4.798	0.202	10.909	PC-QTL	Mn Rb Ni **Cd** Zn		
F_08GF2_aPC5	F	08GF2	aPC5	2	138.302	138.497	28585 ~ 29657	5.838	0.363	16.093	aPC-QTL	Co Rb Fe Cu S		
S		08GF2	Grain	2	139.088	139.088	29516 ~ 29758	4.508	-64.371	9.87	Elemental QTL		LOC_Os02g48450	xylem cysteine proteinase 2 precursor
08GF2_PC4		08GF2	PC4	2	143.252	143.645	30663 ~ 30959	4.051	-0.442	12.787	PC-QTL		LOC_Os02g50680	AAA-type ATPase family protein putative expressed
FUGG_08GF2_aPC7	FUGG	08GF2	aPC7	2	143.252	143.645	30663 ~ 30959	6.287	-0.23	14.846	aPC-QTL		LOC_Os02g50680	AAA-type ATPase family protein putative expressed
GRS_08GF2_aPC8	GRS	08GF2	aPC8	2	143.252	143.645	30663 ~ 30959	4.49	0.033	7.698	aPC-QTL		LOC_Os02g50680	AAA-type ATPase family protein putative expressed
Ca		08GU1	Grain	3	0	0.39	82 ~ 751	3.749	3.307	2.095	Elemental QTL		LOC_Os03g02070	heavy-metal-associated domain-containing protein
Fe		U_mean	Grain	3	0	0.39	82 ~ 751	11.469	0.536	6.243	Elemental QTL			
Rb		07GF	Grain	3	0	0.39	82 ~ 751	5.255	-0.824	11.025	Elemental QTL			
As		15SHH	Shoot	3	0	0.783	82 ~ 858	6.542	-0.1	13.259	Elemental QTL			
GRS_07GF_aPC3	GRS	07GF	aPC3	3	0	0.39	82 ~ 751	7.064	0.085	13.562	aPC-QTL	Ni **Rb** P Mn Zn		
Fe		F_mean	Grain	3	0.195	0.39	10 ~ 751	5.243	0.342	6.744	Elemental QTL			
U_07GU_aPC15	U	07GU	aPC15	3	0.195	0.39	10 ~ 751	4.116	-0.076	3.126	aPC-QTL	**Ca** Mn Sr Ni **Fe**		
07GF_PC7		07GF	PC7	3	0.39	0.39	475 ~ 751	6.202	0.339	13.895	PC-QTL		LOC_Os03g02070	heavy-metal-associated domain-containing protein

Locus symbol[1]	Across environment/tissue	Environment/tissue	Principle component/tissue	Chromosome	Start (cM)	End (cM)	Physical position (Kb)	LOD	ADD[2]	PVE[3]	Group	Elements with top 5 PC loadings[4]	Candidate gene	
													ID	Annotation
F_07GF_aPC16	F	07GF	aPC16	3	0.39	0.783	475 ~ 858	4.038	0.085	8.571	aPC-QTL		LOC_Os03g02070	heavy-metal-associated domain-containing protein
As		02GF	Grain	3	0.783	1.175	731 ~ 1013	3.507	-0.03	5.96	Elemental QTL		LOC_Os03g02380	Major facilitator superfamily domain-containing protein (OsCd1)
Rb		02GF	Grain	3	0.783	1.175	731 ~ 1013	9.515	-1.21	14.981	Elemental QTL			
Rb		08GU1	Grain	3	0.783	1.175	731 ~ 1013	8.467	-2.501	22.84	Elemental QTL			
08GU1_PC2		08GU1	PC2	3	0.783	1.175	731 ~ 1013	4.915	-0.486	8.982	PC-QTL	Ca Mn Sr P **Rb**		
GRS_08GU1_aPC1	GRS	08GU1	aPC1	3	0.783	1.175	731 ~ 1013	6.498	-0.1	20.945	aPC-QTL	Mo Fe S **As** Sr		
GRS_08GU1_aPC3	GRS	08GU1	aPC3	3	0.783	1.175	731 ~ 1013	6.802	0.287	12.286	aPC-QTL	Ni **Rb** P Mn Zn		
GRS_02GF_aPC4	GRS	02GF	aPC4	3	0.783	1.175	731 ~ 1013	8.541	0.198	14.845	aPC-QTL	**Rb** Ni Zn **As** S		
GRS_08GU1_aPC5	GRS	08GU1	aPC5	3	0.783	1.175	731 ~ 1013	10.704	0.144	18.426	aPC-QTL	Mn Sr Zn Cd Co		
GRS_08GU1_aPC7	GRS	08GU1	aPC7	3	0.783	1.175	731 ~ 1013	7.132	0.131	16.972	aPC-QTL	Zn Sr S **Rb** K		
Ca		08GU1	Grain	3	1.175	2.161	698 ~ 1496	3.4	3.16	1.913	Elemental QTL		LOC_Os03g02860	heavy metal-associated domain containing
As		07GF	Grain	3	1.965	2.161	757 ~ 1496	6.531	0.054	6.791	Elemental QTL		LOC_Os03g02670	transporter family protein
Fe		07GF	Grain	3	1.965	3.147	757 ~ 1733	9.87	0.152	0.131	Elemental QTL		LOC_Os03g02860	heavy metal-associated domain containing
Fe		07GU	Grain	3	1.965	2.161	757 ~ 1496	7.916	0.746	21.529	Elemental QTL		LOC_Os03g03180	uncharacterized Cys-rich domain containing protein
Rb		02-06_mean	Grain	3	1.965	2.161	757 ~ 1496	13.717	-0.975	21.945	Elemental QTL			
GRS_02GF_aPC3	GRS	02GF	aPC3	3	1.965	2.161	757 ~ 1496	8.548	0.118	15.933	aPC-QTL	Ni **Rb** P Mn Zn		
GRS_07GU_aPC14	GRS	07GU	aPC14	3	1.965	2.161	757 ~ 1496	6.974	0.023	20.27	aPC-QTL	Fe Mo **As** Cd Sr		
Rb		08GF2	Grain	3	2.951	3.147	1477 ~ 1733	3.034	-0.879	10.2	Elemental QTL		LOC_Os03g03590	Cation/H+ exchanger domain containing protein
GRS_08GF2_aPC3	GRS	08GF2	aPC3	3	2.951	3.147	1477 ~ 1733	7.242	0.09	9.639	aPC-QTL	Ni **Rb** P Mn Zn		
GRS_07GF_aPC7	GRS	07GF	aPC7	3	2.951	3.147	1477 ~ 1733	8.96	0.057	7.293	aPC-QTL	Zn Sr S **Rb** K		
FUGG_06GF_aPC7	FUGG	06GF	aPC7	3	3.147	3.539	1510 ~ 2121	4.677	0.25	9.065	aPC-QTL		LOC_Os03g03500	heavy metal-associated domain containing
As		07GF	Grain	3	3.539	3.734	1720 ~ 2071	5.883	0.053	6.661	Elemental QTL		LOC_Os03g04360	inorganic phosphate transporter
As		15RTH	Root	3	3.539	3.734	1720 ~ 2071	3.548	-0.24	9.173	Elemental QTL		LOC_Os03g04360	inorganic phosphate transporter
Mo		08GU3	Grain	3	3.734	5.314	1690 ~ 2515	9.982	-0.071	2.837	Elemental QTL		LOC_Os03g04920	Similar to MRP-like ABC transporter
Rb		11GGH	Grain	3	5.118	5.314	2211 ~ 2515	4.61	-0.175	7.448	Elemental QTL		LOC_Os03g04920	Similar to MRP-like ABC transporter
06GF_PC1		06GF	PC1	3	5.118	5.314	2211 ~ 2515	4.365	-0.682	12.666	PC-QTL	Cd Ni As Co **Rb**		
As		06GF	Grain	3	5.904	6.49	2150 ~ 2964	3.597	-0.066	8.765	Elemental QTL		LOC_Os03g04920	multidrug resistance-associated protein putative expressed
Cd		08GU3	Grain	3	5.904	6.49	2150 ~ 2964	4.244	-0.035	11.191	Elemental QTL		LOC_Os03g05290	aquaporin protein
Rb		06GF	Grain	3	5.904	6.49	2150 ~ 2964	6.993	-2.304	19.17	Elemental QTL		LOC_Os03g05750	heavy-metal-associated domain-containing protein
Rb		F_mean	Grain	3	5.904	6.49	2150 ~ 2964	7.833	-0.89	15.705	Elemental QTL			
U_08GU1_aPC2	U	08GU1	aPC2	3	5.904	6.49	2150 ~ 2964	4.373	0.431	11.419	aPC-QTL	S **Rb** Sr Cu Co		
GRS_06GF_aPC3	GRS	06GF	aPC3	3	5.904	6.49	2150 ~ 2964	6.931	0.22	20.102	aPC-QTL	Ni **Rb** P Mn Zn		
GRS_06GF_aPC4	GRS	06GF	aPC4	3	5.904	6.1	2150 ~ 2568	6.053	0.394	20.394	aPC-QTL	**Rb** Ni Zn As S		
GRS_08GU1_aPC4	GRS	08GU1	aPC4	3	5.904	6.295	2150 ~ 2742	7.738	0.354	22.133	aPC-QTL	**Rb** Ni Zn As S		
GRS_07GF_aPC10	GRS	07GF	aPC10	3	5.904	5.904	2150 ~ 2399	4.436	-0.044	12.484	aPC-QTL	**As** S Mo Cu Mg		
FUGG_06GF_aPC13	FUGG	06GF	aPC13	3	5.904	5.904	2150 ~ 2399	4.043	-0.129	10.083	aPC-QTL	Cu Ni Zn Mo K		
Rb		06GF	Grain	3	6.1	6.1	2335 ~ 2568	5.257	-2.346	18.344	Elemental QTL		LOC_Os03g05750	heavy-metal-associated domain-containing protein
15RTH_PC5		15RTH	PC5	3	24.137	24.723	6638 ~ 6963	20.648	-0.597	34.862	PC-QTL		LOC_Os03g12530	Manganese transporter
Mn		15RTH	Root	3	24.332	24.723	6477 ~ 6963	10.949	-2.113	28.198	Elemental QTL		LOC_Os03g12530	Manganese transporter
Zn		07GF	Grain	3	25.115	25.31	6921 ~ 7503	5.337	1.15	11.771	Elemental QTL		LOC_Os03g13050	Protein of unknown function DUF607 family protein
08GF2_PC5		08GF2	PC5	3	25.115	25.31	6921 ~ 7503	4.714	-0.324	8.374	PC-QTL	**Zn** Cd Mo Ni S	LOC_Os03g13274	Similar to Low affinity nitrate transporter NRT1.2
GRS_15SHH_aPC2	GRS	15SHH	aPC2	3	27.126	27.126	7302 ~ 7527	6.753	0.16	12.38	aPC-QTL		LOC_Os03g01012	tetraspanin family protein putative expressed
Cd		08GF2	Grain	3	29.1	29.493	8102 ~ 8634	5.241	-0.001	2.775	Elemental QTL		LOC_Os03g14950	Patatin-related phospholipase A
08GU3_PC6		08GU3	PC6	3	31.925	32.71	8358 ~ 9374	6.188	0.354	10.399	PC-QTL		LOC_Os03g15690	Similar to mitochondrial phosphate transporter
03GF_PC12		03GF	PC12	3	36.111	36.423	9597 ~ 9925	4.211	0.169	7.969	PC-QTL		LOC_Os03g17310	calcium-transporting ATPase endoplasmic reticulum-type
K		07GU	Grain	3	51.738	51.933	11873 ~ 12572	4.167	98.753	5.327	Elemental QTL		LOC_Os03g21890	potassium transporter
P		08GF2	Grain	3	51.738	52.129	11873 ~ 12720	3.517	42.074	2.163	Elemental QTL		LOC_Os03g21490	ABC transporter ATP-binding protein
Fe		15SHH	Shoot	3	56.866	57.061	13858 ~ 14577	4.146	-1.48	3.35	Elemental QTL		LOC_Os03g24860	Transporter family protein
08GF2_PC9		08GF2	PC9	3	56.866	57.061	13858 ~ 14577	5.629	-0.255	10.55	PC-QTL	Mn Rb **Fe** Ni Ca	LOC_Os03g24870	Transporter family protein
FUGG_08GF2_aPC1	FUGG	08GF2	aPC1	3	56.866	57.061	13858 ~ 14577	4.635	0.148	5.151	aPC-QTL	Mo P Mg Zn **Fe**		
Sr		03GF	Grain	3	65.599	65.989	15009 ~ 15680	22.666	-0.11	30.047	Elemental QTL		LOC_Os03g26650	heavy metal-associated domain containing protein
Sr		08GU1	Grain	3	65.599	65.989	15009 ~ 15680	4.839	-0.057	18.953	Elemental QTL		LOC_Os03g27040	heavy metal-associated domain containing protein
Sr		02-06_mean	Grain	3	65.599	65.989	15009 ~ 15680	15.376	-0.055	26.091	Elemental QTL			
02GF_PC12		02GF	PC12	3	65.599	65.989	15009 ~ 15680	4.156	-0.166	8.385	PC-QTL	**Sr** Ca K Zn Co		
07GU_PC14		07GU	PC14	3	65.599	65.989	15009 ~ 15680	4.312	-0.16	9.585	PC-QTL	Mg As **Sr** K S		
08GU1_PC15		08GU1	PC15	3	65.599	66.185	15009 ~ 15814	9.348	-0.189	19.523	PC-QTL	Ca **Sr** Mn Mo Mg		
07GF_PC16		07GF	PC16	3	65.599	65.989	15009 ~ 15680	4.191	-0.076	5.628	PC-QTL	Ca **Sr** As Mg Mo		
F_03GF_aPC3	F	03GF	aPC3	3	65.599	65.989	15009 ~ 15680	14.571	-0.567	16.057	aPC-QTL	**Sr** Ca Cu **P** Mg		
FUGG_03GF_aPC4	FUGG	03GF	aPC4	3	65.599	66.185	15009 ~ 15814	11.703	-0.326	14.854	aPC-QTL	Mg Rb Co Zn **P**		
FUGG_03GF_aPC5	FUGG	03GF	aPC5	3	65.599	65.989	15009 ~ 15680	7.414	-0.277	17.571	aPC-QTL	Rb **Sr** As K Co		
FUGG_03GF_aPC6	FUGG	03GF	aPC6	3	65.599	65.989	15009 ~ 15680	5.057	0.148	7.338	aPC-QTL	Co Zn S K Rb		
GRS_02GF_aPC9	GRS	02GF	aPC9	3	65.599	66.185	15009 ~ 15814	5.804	0.027	11.922	aPC-QTL	**Sr** Mo K S Cu		
GRS_03GF_aPC9	GRS	03GF	aPC9	3	65.599	65.989	15009 ~ 15680	12.728	0.043	20.666	aPC-QTL	**Sr** Mo K S Cu		
FUGG_06GF_aPC11	FUGG	06GF	aPC11	3	65.599	65.794	15009 ~ 15553	5.126	0.15	8.265	aPC-QTL	Ca **Sr** Zn S As		
FUGG_08GU1_aPC11	FUGG	08GU1	aPC11	3	65.599	65.989	15009 ~ 15680	4.901	0.124	7.364	aPC-QTL	Ca **Sr** Zn S As		
F_02GF_aPC12	F	02GF	aPC12	3	65.599	66.185	15009 ~ 15814	6.413	-0.156	8.512	aPC-QTL	**Sr** S Ca As Zn		

Locus symbol[1]	Across environment/tissue	Environment/tissue	Principle component/tissue	Chromosome	Start (cM)	End (cM)	Physical position (Kb)	LOD	ADD[2]	PVE[3]	Group	Elements with top 5 PC loadings[4]	Candidate gene	
													ID	Annotation
F_03GF_aPC12	F	03GF	aPC12	3	65.599	65.989	15009 ~ 15680	10.207	-0.243	16.42	aPC-QTL	Sr S Ca As Zn		
FUGG_02GF_aPC12	FUGG	02GF	aPC12	3	65.599	65.794	15009 ~ 15553	6.007	-0.144	10.177	aPC-QTL	S Sr Ni K Rb		
FUGG_03GF_aPC12	FUGG	03GF	aPC12	3	65.599	65.794	15009 ~ 15553	7.388	-0.197	15.849	aPC-QTL	S Sr Ni K Rb		
U_08GU1_aPC13	U	08GU1	aPC13	3	65.599	65.989	15009 ~ 15680	7.174	0.257	17.829	aPC-QTL	K Mg Sr Ni S		
U_08GU1_aPC15	U	08GU1	aPC15	3	65.599	66.185	15009 ~ 15814	6.998	-0.193	17.749	aPC-QTL	Ca Mn Sr Ni Fe		
P		F_mean	Grain	3	65.794	66.185	15186 ~ 15814	4.119	36.271	2.658	Elemental QTL			
Sr		02GF	Grain	3	65.794	66.185	15186 ~ 15814	14.432	-0.074	19.508	Elemental QTL			
Zn		F_mean	Grain	3	66.185	70.965	15310 ~ 16650	3.054	-0.139	0.308	Elemental QTL		LOC_Os03g27040	heavy metal-associated domain containing protein
Sr		06GF	Grain	3	67.38	67.575	15630 ~ 16243	9.622	-0.081	28.274	Elemental QTL		LOC_Os03g27960	Similar to Low affinity calcium transporter
Sr		07GF	Grain	3	67.38	67.575	15630 ~ 16243	4.453	-0.034	6.984	Elemental QTL		LOC_Os03g28120	potassium channel protein
Sr		F_mean	Grain	3	67.38	67.575	15630 ~ 16243	22.104	-0.064	23.994	Elemental QTL			
Sr		U_mean	Grain	3	67.38	67.575	15630 ~ 16243	5.578	-0.041	11.535	Elemental QTL			
06GF_PC5		06GF	PC5	3	67.38	67.575	15630 ~ 16243	4.013	-0.275	7.638	PC-QTL	Rb Cu Mo Zn Mn		
FUGG_03GF_aPC2	FUGG	03GF	aPC2	3	67.38	67.575	15630 ~ 16243	4.437	0.172	8.189	aPC-QTL	As Cu K Ni Cd		
FUGG_02GF_aPC4	FUGG	02GF	aPC4	3	67.38	67.575	15630 ~ 16243	5.33	-0.198	6.902	aPC-QTL	Mg Rb Co Zn P		
F_03GF_aPC6	F	03GF	aPC6	3	67.38	67.575	15630 ~ 16243	4.109	0.271	7.368	aPC-QTL	Mo Fe Mn Zn Co		
GRS_07GF_aPC9	GRS	07GF	aPC9	3	67.38	67.575	15630 ~ 16243	7.047	0.03	14.271	aPC-QTL	Sr Mo K As Cu		
FUGG_08GU1_aPC12	FUGG	08GU1	aPC12	3	67.38	67.575	15630 ~ 16243	5.521	-0.213	15.972	aPC-QTL	S Sr Ni K Rb		
Ca		07GF	Grain	3	72.572	73.276	16795 ~ 17263	3.623	-6.301	4.899	Elemental QTL		LOC_Os03g29850	metal cation transporter
Ca		08GF2	Grain	3	72.572	73.276	16795 ~ 17263	7.33	-6.734	13.97	Elemental QTL		LOC_Os03g29920	Heavy metal transport/detoxification protein domain containing protein
Sr		08GF2	Grain	3	72.572	72.964	16795 ~ 17302	8.803	-0.053	15.659	Elemental QTL			
F_02GF_aPC3	F	02GF	aPC3	3	72.572	72.964	16795 ~ 17302	6.157	-0.43	10.993	aPC-QTL	Sr Ca Cu P Mg		
F_06GF_aPC3	F	06GF	aPC3	3	72.572	73.276	16795 ~ 17263	5.276	-0.428	9.632	aPC-QTL	Sr Ca Cu P Mg		
FUGG_08GF2_aPC3	FUGG	08GF2	aPC3	3	72.572	73.276	16795 ~ 17263	7.637	0.276	12.384	aPC-QTL	Ca Mn Sr Cd As		
FUGG_02GF_aPC5	FUGG	02GF	aPC5	3	72.572	72.964	16795 ~ 17302	5.001	-0.287	12.619	aPC-QTL	Rb Sr As K Co		
Ca		03GF	Grain	3	72.964	73.276	16895 ~ 17416	9.422	-7.272	11.356	Elemental QTL		LOC_Os03g29920	Heavy metal transport/detoxification protein domain containing protein
Ca		F_mean	Grain	3	72.964	73.276	16895 ~ 17416	5.71	-5.477	8.328	Elemental QTL			
03GF_PC3		03GF	PC3	3	72.964	73.276	16895 ~ 17416	9.599	-0.503	11.761	PC-QTL	Ca Sr S Mn Cu		
FUGG_02GF_aPC2	FUGG	02GF	aPC2	3	72.964	73.276	16895 ~ 17416	5.274	0.17	9.987	aPC-QTL	As Cu K Ni Cd		
FUGG_03GF_aPC3	FUGG	03GF	aPC3	3	72.964	73.276	16895 ~ 17416	10.635	0.405	14.782	aPC-QTL	Ca Mn Sr Cd As		
GRS_03GF_aPC6	GRS	03GF	aPC6	3	72.964	73.276	16895 ~ 17416	5.635	-0.061	6.911	aPC-QTL	Mn K P Zn Sr		
GRS_03GF_aPC12	GRS	03GF	aPC12	3	72.964	73.276	16895 ~ 17416	5.419	0.021	6.946	aPC-QTL	S Mo As Sr Ca		
Ca		02GF	Grain	3	73.785	74.097	17130 ~ 17534	6.552	-7.581	11.008	Elemental QTL		LOC_Os03g30460	vacuolar protein sorting-associated protein 52
02GF_PC3		02GF	PC3	3	73.785	74.097	17130 ~ 17534	7.815	0.443	10.062	PC-QTL	Sr Ca Mn Ni P		
FUGG_02GF_aPC3	FUGG	02GF	aPC3	3	73.785	74.097	17130 ~ 17534	8.219	0.29	10.592	aPC-QTL	Ca Mn Sr Cd As		
FUGG_06GF_aPC3	FUGG	06GF	aPC3	3	74.606	74.996	17216 ~ 17853	4.569	0.255	6.856	aPC-QTL		LOC_Os03g30460	vacuolar protein sorting-associated protein 52
GRS_06GF_aPC12	GRS	06GF	aPC12	3	75.892	76.203	17548 ~ 17953	4.553	0.027	8.886	aPC-QTL		LOC_Os03g31340	protein transport protein SEC61 subunit gamma putative
F_06GF_aPC13	F	06GF	aPC13	3	79.525	79.836	19843 ~ 20700	5.535	0.141	6.866	aPC-QTL		LOC_Os03g36540	magnesium-chelatase subunit chlI chloroplast precursor
F_02GF_aPC6	F	02GF	aPC6	3	96.144	96.533	21250 ~ 21637	5.386	0.239	6.478	aPC-QTL		LOC_Os03g38610	MADS box transcription factor
GRS_07GF_aPC12	GRS	07GF	aPC12	3	98.129	98.519	22755 ~ 23002	6.605	0.033	17.335	aPC-QTL		LOC_Os03g41064	Similar to Metal transporter Nramp6
FUGG_08GF2_aPC14	FUGG	08GF2	aPC14	3	98.129	98.519	22755 ~ 23002	4.889	0.098	10.178	aPC-QTL		LOC_Os03g41064	Similar to Metal transporter Nramp6
Co		F_mean	Grain	3	101.223	102.634	23148 ~ 24369	3.698	0.002	1.793	Elemental QTL		LOC_Os03g42020	calcium-transporting ATPase plasma membrane-type
F_07GF_aPC13	F	07GF	aPC13	3	102.242	102.634	23027 ~ 24369	6.255	0.17	11.188	aPC-QTL	As S Ni Mn Cu	LOC_Os03g43580	IQ calmodulin-binding motif family protein
07GF_PC9		07GF	PC9	3	107.393	107.393	24828 ~ 25054	4.243	0.223	8.473	PC-QTL		LOC_Os03g44190	ATG6/Beclin-1 protein
F_03GF_aPC1	F	03GF	aPC1	3	107.393	107.393	24828 ~ 25054	4.357	0.213	4.395	aPC-QTL		LOC_Os03g44190	ATG6/Beclin-1 protein
Cu		02GF	Grain	3	107.901	108.096	25061 ~ 25359	3.566	0.147	4.98	Elemental QTL		LOC_Os03g44610	IQ calmodulin-binding motif domain containing protein
Co		F_mean	Grain	3	126.517	131.43	26439 ~ 27472	3.859	0.002	2.145	Elemental QTL		LOC_Os03g47810	Similar to nitrate and chloride transporter
As		03GF	Grain	3	136.011	136.322	27110 ~ 27985	5.256	0.029	7.462	Elemental QTL		LOC_Os03g48000	Mg2+ transporter protein CorA-like domain containing protein
Cd		11GGH	Grain	3	136.011	136.322	27110 ~ 27985	3.951	-0.003	10.181	Elemental QTL		LOC_Os03g48000	Mg2+ transporter protein CorA-like domain containing protein
Cu		15SHH	Shoot	3	139.244	143.351	28517 ~ 28965	3.758	0.477	4.009	Elemental QTL		LOC_Os03g50250	DnaK family protein putative expressed
Cu		15SHH	Shoot	3	145.266	149.845	28932 ~ 30643	4.188	0.462	3.757	Elemental QTL		LOC_Os03g52090	calcium-transporting ATPase 3 endoplasmic reticulum-type
Cd		03GF	Grain	3	146.673	146.984	29007 ~ 29488	4.538	-0.006	6.013	Elemental QTL		LOC_Os03g51050	peptide transporter PTR2 putative expressed
F_03GF_aPC4	F	03GF	aPC4	3	146.673	146.984	29007 ~ 29488	4.088	0.377	6.691	aPC-QTL	Cd Mn K Mo Sr		
As		02-06_mean	Grain	3	154.893	155.205	30365 ~ 30750	3.341	0.012	1.942	Elemental QTL		LOC_Os03g53110	CorA-like magnesium transporter protein
Mo		03GF	Grain	3	154.893	155.205	30365 ~ 30750	6.475	0.028	10.782	Elemental QTL			
Mo		F_mean	Grain	3	154.893	155.205	30365 ~ 30750	7.709	0.025	11.015	Elemental QTL			
06GF_PC6		06GF	PC6	3	154.893	155.205	30365 ~ 30750	4.655	-0.337	13.431	PC-QTL	Mo As Ni Rb Co		
Mo		03GF	Grain	3	157.123	157.435	30456 ~ 30845	5.089	0.026	8.534	Elemental QTL		LOC_Os03g53780	ammonium transporter 2
F_08GF2_aPC7	F	08GF2	aPC7	3	159.147	159.537	30959 ~ 31392	5.148	0.224	11.708	aPC-QTL		LOC_Os03g54790	ABC transporter ATP-binding protein
As		F_mean	Grain	3	160.317	160.707	32945 ~ 33220	3.468	0.029	3.403	Elemental QTL		LOC_Os03g57200	glutathione S-transferase
Mo		06GF	Grain	3	167.019	167.214	32479 ~ 33122	7.115	0.048	19.514	Elemental QTL		LOC_Os03g57200	glutathione S-transferase
Sr		11GGH	Grain	3	171.37	171.761	33876 ~ 34377	3.523	0.051	10.246	Elemental QTL		LOC_Os03g59640	magnesium-chelatase subunit chlD chloroplast precursor
FUGG_06GF_aPC15	FUGG	06GF	aPC15	3	171.37	171.761	33876 ~ 34377	4.167	-0.119	13.676	aPC-QTL	Ni Mo P K Mn		
FUGG_08GF2_aPC15	FUGG	08GF2	aPC15	3	171.37	171.761	33876 ~ 34377	5.156	-0.122	16.307	aPC-QTL	Ni Mo P K Mn		
K		F_mean	Grain	3	171.565	172.348	34016 ~ 34411	5.316	-82.443	12.132	Elemental QTL		LOC_Os03g59770	EF hand family protein
Mo		06GF	Grain	3	171.956	172.348	33992 ~ 34411	7.233	0.046	17.3	Elemental QTL		LOC_Os03g59770	EF hand family protein

Locus symbol[1]	Across environment/tissue	Environment/tissue	Principle component/tissue	Chromosome	Start (cM)	End (cM)	Physical position (Kb)	LOD	ADD[2]	PVE[3]	Group	Elements with top 5 PC loadings[4]	Candidate gene	
													ID	Annotation
GRS_07GF_aPC1	GRS	07GF	aPC1	3	171.956	172.348	33992 ~ 34411	4.169	-0.033	6.394	aPC-QTL	Mo Fe S As Sr	LOC_Os03g59790	EF hand family protein
Mn		07GF	Grain	3	172.939	173.329	34394 ~ 34776	3.701	1.75	9.517	Elemental QTL		LOC_Os03g60820	transporter major facilitator superfamily domain containing protein
07GF_PC1		07GF	PC1	3	173.329	173.915	34655 ~ 35388	7.837	-0.765	18.063	PC-QTL	Mg K P Rb Zn	LOC_Os03g61290	Cation/proton antiporter
F_07GF_aPC2	F	07GF	aPC2	3	173.329	173.915	34655 ~ 35388	8.149	0.532	15.286	aPC-QTL	Mg Cu P As Cd	LOC_Os03g62270	MATE efflux family protein
FUGG_06GF_aPC8	FUGG	06GF	aPC8	3	173.329	173.915	34655 ~ 35388	5.484	-0.137	7.558	aPC-QTL	Fe Mg Cd P Mn		
Mg		08GF2	Grain	3	173.525	173.915	34655 ~ 35388	5.75	-47.435	11.807	Elemental QTL			
Mg		U_mean	Grain	3	173.525	173.915	34655 ~ 35388	7.846	-50.146	16.643	Elemental QTL			
Ni		07GU	Grain	3	175.701	176.693	35367 ~ 35720	3.796	-0.542	10.384	Elemental QTL		LOC_Os03g62650	Ion channel DMI1
Co		08GU3	Grain	3	177.284	179.309	35422 ~ 35967	4.54	-0.002	0.162	Elemental QTL		LOC_Os03g62650	Ion channel DMI1
P		F_mean	Grain	3	179.309	179.309	35715 ~ 35967	4.191	-57.769	6.742	Elemental QTL		LOC_Os03g63420	OsGrx_S14 - glutaredoxin subgroup II expressed
P		U_mean	Grain	3	179.309	179.309	35715 ~ 35967	3.944	-73.251	8.296	Elemental QTL		LOC_Os03g63420	OsGrx_S14 - glutaredoxin subgroup II expressed
P		07GF	Grain	3	179.9	179.9	35596 ~ 35836	5.533	-82.364	7.573	Elemental QTL		LOC_Os03g63420	OsGrx_S14 - glutaredoxin subgroup II expressed
FUGG_07GF_aPC4	FUGG	07GF	aPC4	3	179.9	179.9	35596 ~ 35836	6.319	0.35	13.154	aPC-QTL	Mg Rb Co Zn P		
Fe		11GGH	Grain	4	22.085	22.085	7803 ~ 8065	4.331	0.219	6.972	Elemental QTL		LOC_Os04g14130	Pentatricopeptide repeat domain containing protein
Cu		08GU3	Grain	4	62.146	62.457	20459 ~ 20886	5.427	0.554	11.801	Elemental QTL		LOC_Os04g34530	integral membrane protein DUF6 containing protein
Sr		08GU3	Grain	4	62.146	62.457	20459 ~ 20886	4.281	-0.012	4.297	Elemental QTL		LOC_Os04g34530	integral membrane protein DUF6 containing protein
Ni		11GGH	Grain	4	219.8	220.191	33503 ~ 34084	3.788	-0.074	4.653	Elemental QTL		LOC_Os04g56570	heavy metal-associated domain containing protein
GRS_08GU1_aPC6	GRS	08GU1	aPC6	4	219.8	220.191	33503 ~ 34084	4.187	0.097	11.96	aPC-QTL	Mn K P Zn Sr	LOC_Os04g57200	heavy metal transport Fdetoxification protein putative
Mg		06GF	Grain	4	219.996	219.996	32593 ~ 32825	3.31	-39.786	9.881	Elemental QTL		LOC_Os04g55210	chloride channel protein
Zn		08GF2	Grain	4	222.842	222.842	33160 ~ 33413	3.744	0.813	7.264	Elemental QTL		LOC_Os04g56330	ABC transporter ATP-binding protein
15RTH_PC4		15RTH	PC4	4	222.842	222.842	33160 ~ 33413	5.713	0.342	9.717	PC-QTL	K Mg Zn Ni As		
Ni		08GF2	Grain	5	0	0.312	26 ~ 183	3.252	-0.1	3.256	Elemental QTL		LOC_Os05g01240	AML1 putative expressed
FUGG_08GF2_aPC12	FUGG	08GF2	aPC12	5	0	0.312	26 ~ 183	4.309	-0.151	10.632	aPC-QTL	S Sr Ni K Rb		
S		07GF	Grain	5	1.019	1.331	2258 ~ 2547	6.88	-47.237	13.247	Elemental QTL		LOC_Os05g04990	adenosylmethionine decarboxylase putative expressed
S		08GF2	Grain	5	1.019	1.331	2258 ~ 2547	6.688	-74.081	12.947	Elemental QTL		LOC_Os05g04990	adenosylmethionine decarboxylase putative expressed
S		U_mean	Grain	5	1.019	1.331	2258 ~ 2547	8.145	-62.772	16.505	Elemental QTL		LOC_Os05g04990	adenosylmethionine decarboxylase putative expressed
Mg		08GU1	Grain	5	2.644	3.036	1461 ~ 2660	3.162	21.27	2.326	Elemental QTL		LOC_Os05g03780	cation efflux family protein putative expressed
S		F_mean	Grain	5	2.644	3.231	1461 ~ 2759	8.008	-50.696	12.698	Elemental QTL		LOC_Os05g05460	Similar to calcium ion binding protein
Co		08GF2	Grain	5	4.214	4.214	1927 ~ 2149	4.716	-0.003	9.274	Elemental QTL		LOC_Os05g04380	peroxidase precursor putative expressed
FUGG_06GF_aPC9	FUGG	06GF	aPC9	5	4.214	4.214	1927 ~ 2149	4.942	-0.067	2.053	aPC-QTL	Fe Cd Mn Zn Rb		
FUGG_08GU1_aPC8	FUGG	08GU1	aPC8	5	7.396	7.591	2294 ~ 3528	4.79	0.223	9.428	aPC-QTL		LOC_Os05g05590	Similar to Na+/H+ antiporter
F_08GF2_aPC9	F	08GF2	aPC9	5	7.396	7.396	2294 ~ 2522	4.325	-0.25	13.655	aPC-QTL		LOC_Os05g05160	Similar to Mitogen-activated protein kinase 14.
S		08GU1	Grain	5	9.577	9.577	2797 ~ 3089	4.059	-66.13	11.626	Elemental QTL		LOC_Os05g05460	Similar to calcium ion binding protein.
Mo		07GU	Grain	5	16.782	17.094	4908 ~ 5244	3.795	-0.016	5.603	Elemental QTL		LOC_Os05g08930	chloroplast lumen common family protein putative expressed
03GF_PC10		03GF	PC10	5	68.654	69.044	15782 ~ 16219	4.03	-0.151	5.373	PC-QTL		LOC_Os05g27790	membrane associated DUF588 domain containing protein
As		06GF	Grain	5	73.667	73.667	17432 ~ 17675	4.813	-0.054	5.656	Elemental QTL		LOC_Os05g30570	heavy metal-associated domain containing protein
F_03GF_aPC16	F	03GF	aPC16	5	73.667	73.667	17432 ~ 17675	17.478	0.073	5.965	aPC-QTL	P Ni Mg K Rb		
Cd		F_mean	Grain	5	76.703	76.703	16965 ~ 17194	4.702	0.002	2.933	Elemental QTL		LOC_Os05g29050	phospholipase D p1 putative expressed
P		08GU3	Grain	5	76.703	76.898	16965 ~ 17377	5.366	-119.264	1.712	Elemental QTL		LOC_Os05g29860	Mitochondrial carrier protein domain containing protein
Mn		02-06_mean	Grain	5	78.298	78.298	17682 ~ 17908	4.113	1.695	9.792	Elemental QTL		LOC_Os05g30570	heavy metal-associated domain containing protein expressed
As		07GU	Grain	5	79.827	79.827	19868 ~ 20262	4.022	-0.006	6.874	Elemental QTL		LOC_Os05g33910	MATE
07GU_PC2		07GU	PC2	5	90.551	90.94	21015 ~ 21469	4.667	0.407	6.701	PC-QTL		LOC_Os05g35740	Similar to Pi starvation-induced protein
GRS_07GU_aPC15	GRS	07GU	aPC15	5	90.551	90.94	21015 ~ 21469	4.518	0.018	10.023	aPC-QTL		LOC_Os05g35740	Similar to Pi starvation-induced protein
07GF_PC6		07GF	PC6	5	97.401	97.714	21342 ~ 22849	5.476	0.311	9.82	PC-QTL		LOC_Os05g38670	cation efflux family protein
FUGG_06GF_aPC12	FUGG	06GF	aPC12	5	97.401	97.714	21342 ~ 22849	4.573	-0.145	10.991	aPC-QTL		LOC_Os05g38670	cation efflux family protein
Zn		02-06_mean	Grain	5	100.027	100.027	23019 ~ 23246	3.239	0.749	9.558	Elemental QTL		LOC_Os05g38670	cation efflux family protein
FUGG_07GU_aPC5	FUGG	07GU	aPC5	6	0	0.39	19 ~ 201	4.051	-0.198	10.598	aPC-QTL		LOC_Os06g01260	glutathione gamma-glutamylcysteinyltransferase 1 putative expressed
P		15RTH	Root	6	17.423	17.617	1748 ~ 2031	5.646	218.824	13.964	Elemental QTL		LOC_Os06g04250	phosphate-induced protein 1 conserved region domain containing protein
15RTH_PC6		15RTH	PC6	6	18.602	18.602	1995 ~ 2216	4.18	0.337	12.2	PC-QTL		LOC_Os06g04900	transporter family protein
15SHH_PC7		15SHH	PC7	6	32.627	32.939	3567 ~ 4015	5.137	0.293	10.335	PC-QTL		LOC_Os06g08170	transporter major facilitator family
Co		11GGH	Grain	6	77.648	77.842	6891 ~ 7095	3.404	0.007	5.618	Elemental QTL		LOC_Os06g12630	glutathione S-transferase N-terminal domain
08GU3_PC8		08GU3	PC8	6	78.825	78.825	7166 ~ 7413	4.697	0.341	15.074	PC-QTL		LOC_Os06g13200	proton-dependent oligopeptide transport
GRS_03GF_aPC7	GRS	03GF	aPC7	6	80.119	80.821	7576 ~ 7991	5.019	-0.057	8.727	aPC-QTL		LOC_Os06g14030	potassium channel SKOR
Cd		02GF	Grain	6	110.491	110.88	13728 ~ 16296	3.768	-0.003	6.861	Elemental QTL		LOC_Os06g27920	zinc ion binding protein
F_07GF_aPC12	F	07GF	aPC12	6	113.886	114.198	18934 ~ 19410	4.365	-0.135	6.851	aPC-QTL		LOC_Os06g32600	THION15 - Plant thionin family protein precursor
Co		07GF	Grain	6	120.343	120.736	20102 ~ 20456	6.262	-0.007	14.201	Elemental QTL		LOC_Os06g35060	heavy metal-associated domain containing protein
Co		F_mean	Grain	6	120.343	120.736	20102 ~ 20456	4.161	-0.004	8.527	Elemental QTL		LOC_Os06g35060	heavy metal-associated domain containing protein
Co		02-06_mean	Grain	6	120.343	120.736	20102 ~ 20456	4.666	-0.004	7.727	Elemental QTL		LOC_Os06g35060	heavy metal-associated domain containing protein
Cd		02-06_mean	Grain	6	122.746	122.942	20927 ~ 21271	3.829	-0.005	6.072	Elemental QTL		LOC_Os06g36090	ABC-2 type transporter
Ni		08GF2	Grain	6	124.137	124.449	21243 ~ 21675	5.546	-0.127	5.404	Elemental QTL		LOC_Os06g36330	MATE domain containing protein
Ni		F_mean	Grain	6	124.137	124.449	21243 ~ 21675	5.615	-0.039	4.931	Elemental QTL		LOC_Os06g36330	MATE domain containing protein
F_06GF_aPC4	F	06GF	aPC4	7	25.793	26.183	2692 ~ 3014	4.276	-0.312	5.404	aPC-QTL		LOC_Os07g06130	Nramp ion-transporter family protein Ethylene signaling pathway
P		02GF	Grain	7	26.183	26.495	2745 ~ 3219	3.099	76.841	6.5	Elemental QTL		LOC_Os07g06130	Nramp ion-transporter family protein Ethylene signaling pathway
P		06GF	Grain	7	26.495	27.402	2934 ~ 3285	3.031	59.629	3.698	Elemental QTL		LOC_Os07g06130	Nramp ion-transporter family protein Ethylene signaling pathway
GRS_06GF_aPC5	GRS	06GF	aPC5	7	30.515	30.515	3722 ~ 3958	6.313	0.11	13.87	aPC-QTL		LOC_Os07g07790	LTPL75 - Protease inhibitor Fseed storage FLTP family protein

Locus symbol[1]	Across environment/tissue	Environment/tissue	Principle component/tissue	Chromosome	Start (cM)	End (cM)	Physical position (Kb)	LOD	ADD[2]	PVE[3]	Group	Elements with top 5 PC loadings[4]	Candidate gene	
													ID	Annotation
Ca		15SHH	Shoot	7	33.839	34.035	4014 ~ 4330	3.863	-98.295	6.458	Elemental QTL		LOC_Os07g08300	major facilitator superfamily antiporter
GRS_15SHH_aPC13	GRS	15SHH	aPC13	7	33.839	34.035	4014 ~ 4330	5.133	-0.047	8.55	aPC-QTL	Cu Co Cd As S		
FUGG_08GU3_aPC15	FUGG	08GU3	aPC15	7	39.557	39.869	5035 ~ 5303	6.309	-0.175	17.06	aPC-QTL		LOC_Os07g09600	vesicle-associated membrane protein
Mn		08GU1	Grain	7	51.187	51.382	7007 ~ 7904	3.122	2.075	8.407	Elemental QTL		LOC_Os07g12900	Heavy metal-transporting P1B-ATPase (OsHMA3)
15SHH_PC9		15SHH	PC9	7	51.187	51.382	7007 ~ 7904	4.157	0.228	7.693	PC-QTL	Rb K Ni As Zn		
GRS_08GU1_aPC5	GRS	08GU1	aPC5	7	51.187	51.382	7007 ~ 7904	7.341	0.116	12.01	aPC-QTL	Mn Sr Zn Cd Co		
Mn		06GF	Grain	7	52.367	52.756	8331 ~ 9044	5.38	2.715	13.594	Elemental QTL		LOC_Os07g15370	Natural resistance associated macrophage protein 5 (OsNRAMP5)
15SHH_PC16		15SHH	PC16	7	52.367	52.756	8331 ~ 9044	8.247	-0.126	17.641	PC-QTL	Mn Cd Sr Ca As		
F_06GF_aPC6	F	06GF	aPC6	7	52.367	52.756	8331 ~ 9044	7.325	0.379	15.004	aPC-QTL	Mo Fe Mn Zn Co		
GRS_06GF_aPC6	GRS	06GF	aPC6	7	52.367	52.756	8331 ~ 9044	6.841	0.092	18.039	aPC-QTL	Mn K P Zn Sr		
Mg		02-06_mean	Grain	7	53.847	54.159	8265 ~ 8937	4.107	-35.119	10.927	Elemental QTL		LOC_Os07g15460	Natural resistance associated macrophage protein 1 (OsNRAMP1)
GRS_08GF2_aPC5	GRS	08GF2	aPC5	7	55.531	55.531	9360 ~ 9598	7.266	0.053	10.94	aPC-QTL		LOC_Os07g16130	acetyltransferase GNAT family putative expressed
Mn		07GF	Grain	7	56.316	56.628	9001 ~ 10330	3.589	1.729	9.29	Elemental QTL		LOC_Os07g17120	late embryogenesis abundant protein
Mn		F_mean	Grain	7	56.316	56.628	9001 ~ 10330	6.881	1.973	14.06	Elemental QTL		LOC_Os07g17120	late embryogenesis abundant protein
GRS_08GF2_aPC16	GRS	08GF2	aPC16	7	63.245	63.634	12866 ~ 13067	4.484	-0.002	8.318	aPC-QTL		LOC_Os07g22930	starch synthase putative expressed
FUGG_07GF_aPC15	FUGG	07GF	aPC15	7	82.128	82.323	10259 ~ 13394	5.062	-0.096	12.081	aPC-QTL		LOC_Os07g20340	heavy metal-associated domain containing protein
Mn		U_mean	Grain	7	83.113	83.308	14625 ~ 14955	4.717	2.246	15.333	Elemental QTL		LOC_Os07g25710	MYB-CC family protein Pi-starvation signaling
GRS_07GF_aPC5	GRS	07GF	aPC5	7	83.113	83.308	14625 ~ 14955	4.902	0.051	10.381	aPC-QTL	Mn Sr Zn Cd Co		
Mo		08GF2	Grain	7	90.678	90.678	16645 ~ 16866	6.578	0.037	16.518	Elemental QTL		LOC_Os07g28480	glutathione S-transferase
Mo		07GU	Grain	7	90.678	90.678	16645 ~ 16866	3.541	0.021	9.974	Elemental QTL			
Mo		F_mean	Grain	7	90.678	90.678	16645 ~ 16866	8.35	0.027	12.89	Elemental QTL			
Mo		U_mean	Grain	7	90.678	90.678	16645 ~ 16866	9.998	0.034	14.77	Elemental QTL			
Mo		02-06_mean	Grain	7	90.678	90.678	16645 ~ 16866	3.466	0.028	7.952	Elemental QTL			
FUGG_03GF_aPC15	FUGG	03GF	aPC15	7	90.678	90.678	16645 ~ 16866	5.125	-0.072	9.583	aPC-QTL	Ni Mo P K Mn		
Mo		08GF2	Grain	7	92.078	92.47	16902 ~ 17246	7.946	0.038	17.239	Elemental QTL		LOC_Os07g29230	LTPL63 - Protease inhibitor Fseed storage FLTP family protein precursor
FUGG_08GF2_aPC15	FUGG	08GF2	aPC15	7	92.078	92.078	16902 ~ 17141	4.43	-0.115	14.652	aPC-QTL	Ni Mo P K Mn		
GRS_03GF_aPC1	GRS	03GF	aPC1	7	93.061	93.451	17092 ~ 17521	4.389	-0.037	10.981	aPC-QTL		LOC_Os07g29230	LTPL63 - Protease inhibitor Fseed storage FLTP family protein precursor
As		02-06_mean	Grain	8	1.793	1.988	40 ~ 737	3.558	-0.019	4.778	Elemental QTL		LOC_Os08g01120	OsMOT1;1
Mo		02-06_mean	Grain	8	1.793	1.988	40 ~ 737	3.494	0.026	6.809	Elemental QTL			
FUGG_11GGH_aPC13	FUGG	11GGH	aPC13	8	1.793	1.988	40 ~ 737	5.718	0.17	7.132	aPC-QTL	Cu Ni Zn Mo K		
08GU1_PC3		08GU1	PC3	8	11.016	11.718	995 ~ 1387	6.477	-0.439	11.241	PC-QTL		LOC_Os08g02450	Cation/H+ exchanger domain containing protein
FUGG_11GGH_aPC12	FUGG	11GGH	aPC12	8	22.41	23.312	2479 ~ 2754	6.005	-0.084	3.01	aPC-QTL		LOC_Os08g04890	OsCML32 - Calmodulin-related calcium sensor protein
Mg		06GF	Grain	8	35.397	35.591	3396 ~ 3608	3.071	30.251	5.713	Elemental QTL			
P		06GF	Grain	8	35.397	35.591	3396 ~ 3608	3.153	78.171	7.066	Elemental QTL			
FUGG_07GF_aPC11	FUGG	07GF	aPC11	8	35.397	35.591	3396 ~ 3608	5.676	-0.09	5.392	aPC-QTL	Ca Sr Zn S As		
06GF_PC8		06GF	PC8	8	37.197	37.705	3602 ~ 3939	5.05	0.253	9.791	PC-QTL		LOC_Os08g07010	ABC-2 type transporter domain containing protein
GRS_11GGH_aPC11	GRS	11GGH	aPC11	8	37.197	37.509	3602 ~ 3876	4.361	0.06	7.278	aPC-QTL		LOC_Os08g06440	secretory carrier-associated membrane protein 5
Cu		03GF	Grain	8	38.724	39.508	3914 ~ 4481	6.565	-0.206	9.481	Elemental QTL		LOC_Os08g07010	ABC-2 type transporter domain containing protein
Cu		02GF	Grain	8	39.116	39.508	4110 ~ 4481	3.505	-0.171	6.761	Elemental QTL		LOC_Os08g07450	glutaredoxin protein putative expressed
F_03GF_aPC3	F	03GF	aPC3	8	40.695	41.084	4569 ~ 5234	8.334	0.465	10.813	aPC-QTL		LOC_Os08g08060	vacuolar protein sorting-associated protein 18
S		U_mean	Grain	8	49.811	50.006	6403 ~ 6794	5.393	-47.078	9.283	Elemental QTL		LOC_Os08g11220	expressed protein
Mo		15SHH	Shoot	8	50.006	50.707	6478 ~ 7234	3.822	2.115	7.09	Elemental QTL		LOC_Os08g12160	expressed protein
15SHH_PC2		15SHH	PC2	8	50.006	50.707	6478 ~ 7234	5.114	-0.471	8.761	PC-QTL	Mo Ca Sr Rb Fe		
F_08GF2_aPC15	F	08GF2	aPC15	9	8.475	8.475	5700 ~ 5945	4.4	0.166	9.358	aPC-QTL		LOC_Os09g10620	seed maturation protein LEA 4
06GF_PC9		06GF	PC9	9	16.757	16.757	7350 ~ 7583	5.04	-0.255	13.303	PC-QTL		LOC_Os09g12790	potassium channel protein
P		11GGH	Grain	9	21.795	21.991	8638 ~ 9061	3.623	150.184	6.123	Elemental QTL		LOC_Os09g14700	2-aminoethanethiol dioxygenase
GRS_11GGH_aPC8	GRS	11GGH	aPC8	9	24.769	25.552	9295 ~ 9869	4.977	0.078	9.018	aPC-QTL		LOC_Os09g15330	transporter family protein
F_08GF2_aPC10	F	08GF2	aPC10	9	24.769	25.161	9295 ~ 9580	5.183	-0.223	9.416	aPC-QTL		LOC_Os09g15330	transporter family protein
As		03GF	Grain	9	83.078	83.39	20581 ~ 20921	5.194	-0.029	6.572	Elemental QTL		LOC_Os09g35780	C2 calcium/lipid-binding region CaLB domain containing protein
As		02-06_mean	Grain	9	83.078	83.39	20581 ~ 20921	3.903	-0.02	5.386	Elemental QTL		LOC_Os09g35780	C2 calcium/lipid-binding region CaLB domain containing protein
Rb		03GF	Grain	9	95.087	95.283	22149 ~ 22591	6.295	-0.731	12.116	Elemental QTL		LOC_Os09g38772	Molybdenum cofactor sulfurase C-terminal domain containing protein
GRS_03GF_aPC4	GRS	03GF	aPC4	9	95.087	95.283	22149 ~ 22591	6.096	0.121	11.642	aPC-QTL	Rb Ni Zn As S	LOC_Os09g38777	Molybdenum cofactor sulfurase C-terminal domain containing protein
													LOC_Os09g38960	potassium transporter
Mg		07GF	Grain	10	87.752	87.752	13975 ~ 14209	4.207	31.029	5.895	Elemental QTL		LOC_Os10g27050	OsPP2Ac-4 - Phosphatase 2A isoform 4 belonging to family 2 expressed
07GF_PC5		07GF	PC5	10	92.626	92.938	17153 ~ 17433	8.244	0.516	20.981	PC-QTL		LOC_Os10g33210	peptide transporter PTR3-A
Cd		U_mean	Grain	10	93.641	93.952	17262 ~ 17851	4.28	-0.001	8.102	Elemental QTL		LOC_Os10g33210	peptide transporter PTR3-A putative expressed
GRS_07GF_aPC9	GRS	07GF	aPC9	10	93.641	93.952	17262 ~ 17851	6.047	0.031	15.018	aPC-QTL	Sr Mo K As Cu		
FUGG_07GF_aPC10	FUGG	07GF	aPC10	10	93.641	93.952	17262 ~ 17851	6.381	-0.173	18.777	aPC-QTL	As Zn Cd Mn Ca		
Ca		08GU3	Grain	10	105.16	106.755	19532 ~ 19828	21.817	-9.481	2.942	Elemental QTL		LOC_Os10g36924	aquaporin protein
F_08GF2_aPC6	F	08GF2	aPC6	10	108.35	108.74	19686 ~ 20840	6.814	-0.298	12.223	aPC-QTL		LOC_Os10g37920	MATE efflux family protein
Mg		03GF	Grain	10	109.525	109.525	20819 ~ 21071	4.173	31.05	6.013	Elemental QTL		LOC_Os10g39210	heavy metal-associated domain containing protein
S		03GF	Grain	10	118.967	118.967	21215 ~ 21439	3.18	-49.514	5.201	Elemental QTL		LOC_Os10g39980	Divalent ion symporter domain containing protein
F_06GF_aPC2	F	06GF	aPC2	11	0	0.392	553 ~ 755	5.401	0.597	13.597	aPC-QTL		LOC_Os11g02464	vacuolar-sorting receptor precursor
Mg		06GF	Grain	11	4.262	4.457	1104 ~ 1362	4.869	-43.989	11.002	Elemental QTL		LOC_Os11g03240	MATE efflux family protein
FUGG_06GF_aPC4	FUGG	06GF	aPC4	11	4.262	4.457	1104 ~ 1362	6.491	0.392	18.702	aPC-QTL	Mg Rb Co Zn P	LOC_Os11g03484	MATE efflux family protein

Locus symbol[1]	Across environment/tissue	Environment/tissue	Principle component/tissue	Chromosome	Start (cM)	End (cM)	Physical position (Kb)	LOD	ADD[2]	PVE[3]	Group	Elements with top 5 PC loadings[4]	Candidate gene ID	Candidate gene Annotation
													LOC_Os11g03500	MATE efflux family protein
Ni		15SHH	Shoot	11	5.652	6.437	1343 ~ 2237	3.435	0.046	2.889	Elemental QTL		LOC_Os11g05010	heavy-metal-associated domain-containing protein
Mg		02GF	Grain	11	12.889	13.084	2667 ~ 2976	4.77	-37.921	8.223	Elemental QTL		LOC_Os11g06100	carrier putative expressed
F_02GF_aPC2	F	02GF	aPC2	11	12.889	13.084	2667 ~ 2976	5.929	0.389	11.084	aPC-QTL	**Mg** Cu P As Cd	LOC_Os11g06110	carrier putative expressed
Mn		03GF	Grain	11	14.279	14.279	2796 ~ 3031	4.434	-1.848	5.222	Elemental QTL		LOC_Os11g06100	carrier putative expressed
GRS_02GF_aPC2	GRS	02GF	aPC2	11	14.279	14.279	2796 ~ 3031	4.059	-0.041	4.875	aPC-QTL	P Mg **Mn** Ca Co		
GRS_03GF_aPC2	GRS	03GF	aPC2	11	14.279	14.279	2796 ~ 3031	4.421	-0.053	9.077	aPC-QTL	P Mg **Mn** Ca Co		
GRS_03GF_aPC5	GRS	03GF	aPC5	11	14.279	14.279	2796 ~ 3031	4.989	-0.058	9.437	aPC-QTL	**Mn** Sr Zn Cd Co		
F_03GF_aPC8	F	03GF	aPC8	11	14.279	14.279	2796 ~ 3031	5.204	-0.297	11.179	aPC-QTL	Cd Co Zn Rb Ni		
Mg		02GF	Grain	11	17.871	18.263	3588 ~ 3894	4.303	-33.591	6.578	Elemental QTL		LOC_Os11g07600	ABC-2 type transporter domain containing protein
GRS_06GF_aPC2	GRS	06GF	aPC2	11	17.871	18.263	3588 ~ 3894	4.803	-0.067	11.445	aPC-QTL	P **Mg** Mn Ca Co		
Mg		F_mean	Grain	11	19.458	19.458	3833 ~ 4055	4.073	-23.985	5.465	Elemental QTL		LOC_Os11g07600	ABC-2 type transporter domain containing protein
FUGG_08GF2_aPC16	FUGG	08GF2	aPC16	11	33.143	33.533	7029 ~ 7296	6.139	-0.08	9.024	aPC-QTL		LOC_Os11g12740	peptide transporter PTR2
03GF_PC13		03GF	PC13	11	48.399	48.594	9759 ~ 10282	4.475	0.145	6.2	PC-QTL		LOC_Os11g18170	glutathione peroxidase
GRS_15RTH_aPC13	GRS	15RTH	aPC13	11	63.02	63.721	16254 ~ 16666	4.545	0.134	6.836	aPC-QTL		LOC_Os11g29490	ATPase P-type K/Mg/Cd/Cu/Zn/Na/Ca/Na/H-transporter family protein
Sr		15SHH	Shoot	11	83.595	83.79	19351 ~ 19879	4.03	-0.129	6.032	Elemental QTL		LOC_Os11g34350	ATP-binding cassette sub-family E member (OsABCE2)
Zn		15SHH	Shoot	11	83.595	83.79	19351 ~ 19879	4.62	2.861	10.328	Elemental QTL			
15SHH_PC1		15SHH	PC1	11	83.595	83.79	19351 ~ 19879	7.404	-0.74	13.681	PC-QTL	Cd Mn **Zn** S As		
GRS_15SHH_aPC5	GRS	15SHH	aPC5	11	83.595	83.79	19351 ~ 19879	4.337	0.199	7.551	aPC-QTL	Mn **Sr** **Zn** Cd Co		
GRS_15SHH_aPC6	GRS	15SHH	aPC6	11	83.595	83.79	19351 ~ 19879	7.16	0.371	13.466	aPC-QTL	Mn K P **Zn** **Sr**		
Cd		15RTH	Root	11	83.79	84.18	19608 ~ 19944	6.287	3.972	12.73	Elemental QTL		LOC_Os11g34660	LTPL98 - Protease inhibitor Fseed storage FLTP family protein precursor
Cd		15SHH	Shoot	11	83.79	84.18	19608 ~ 19944	4.294	0.513	11.294	Elemental QTL		LOC_Os11g34660	LTPL98 - Protease inhibitor Fseed storage FLTP family protein precursor
Mn		15SHH	Shoot	11	83.79	84.18	19608 ~ 19944	6.924	10.941	14.777	Elemental QTL		LOC_Os11g34660	LTPL98 - Protease inhibitor Fseed storage FLTP family protein precursor
Sr		15RTH	Root	11	89.46	89.655	20598 ~ 20934	3.009	-0.613	4.977	Elemental QTL		LOC_Os11g36240	pectinesterase putative expressed
08GU1_PC12		08GU1	PC12	11	89.46	89.655	20598 ~ 20934	4.527	0.226	11.833	PC-QTL	Mn Ni K **Sr** Rb		
GRS_15RTH_aPC11	GRS	15RTH	aPC11	12	0	0.393	54 ~ 1267	4.524	-0.152	11.26	aPC-QTL		LOC_Os12g01580	MATE efflux family protein
U_08GU1_aPC3	U	08GU1	aPC3	12	21.601	21.796	7469 ~ 10488	4.943	0.316	6.585	aPC-QTL		LOC_Os12g13720	Plant PDR ABC transporter associated domain containing protein
Ca		08GU1	Grain	12	113.623	113.818	25482 ~ 26203	4.352	-5.821	6.49	Elemental QTL		LOC_Os12g42200	ATCHX putative expressed
S		15SHH	Shoot	12	113.623	113.818	25482 ~ 26203	5.326	162.836	5.182	Elemental QTL		LOC_Os12g42130	MATE efflux family protein
Mg		03GF	Grain	12	117.197	117.197	25394 ~ 25616	5.28	-37.56	9.801	Elemental QTL		LOC_Os12g41110	OsCML5 - Calmodulin-related calcium sensor protein expressed
Mg		07GU	Grain	12	121.465	121.857	25622 ~ 26855	4.421	-42.14	9.655	Elemental QTL		LOC_Os12g42200	ATCHX putative expressed
Mo		08GU3	Grain	12	122.849	125.047	26689 ~ 27152	11.267	0.058	1.901	Elemental QTL		LOC_Os12g43440	thaumatin putative expressed
P		07GU	Grain	12	122.849	122.849	26951 ~ 27194	3.674	-90.206	7.023	Elemental QTL		LOC_Os12g43820	GCRP5 - Glycine and cysteine rich family protein precursor

[1]: 02GF: grains (G) of RILs grown under flooded (F) on 2002;
03GF: grains (G) of RILs grown under flooded (F) on 2003;
06GF: grains (G) of RILs grown under flooded (F) on 2006;
07GF: grains (G) of RILs grown under flooded (F) on 2007;
07GU: grains (G) of RILs grown under unflooded (U) on 2007;
08GF2: grains (G) of RILs grown under flooded (F) in the field site number 2 on 2008;
08GU1: grains (G) of RILs grown under unflooded (U) in the field site number 1 on 2008;
08GU3: grains (G) of RILs grown under unflooded (U) in the field site number 3 on 2008;
11GGH: grains (G) of RILs grown in a greenhouse (GH) condition on 2011;
15RTH: roots (RT) of RILs grown hydroponically (H) on 2015;
15SHH: shoots (SH) of RILs grown hydroponically (H) on 2015;
F: 02GF+03GF+06GF+07GF+08GF2;
U: 07GU+08GU1+08GU3;
FUGG: F+U+11GGH;
GRS: FUGG+15RTH+15SHH.

[2]ADD: additive effect

[3]PVE: perventage of variance explained

[4]: the elements in red represent the elements conresponding to the co-localized elemental QTLs.

Supplementary Table 13. The sequence variations of candidate genes for the QTL clusters in Table 1.

QTL cluster	Chromosome	Candidate gene		Candidate gene sequence variation				
		ID	Annotation	Variation type	LM	TQ	Effect	Codon change
1	1	LOC_Os01g08660	Aquaporin protein (SIP1;1)	Indel	-	CTGCAATACACA	12 bp deletion in promoter	-
2	2	LOC_Os02g10290	Heavy metal-transporting P1B type ATPase (OsHMA4)	SNP	C	T	Nonsynonymous	gTc/gCc
3	2	LOC_Os02g26700	Cation transport regulator-like protein (OsARP)	SNP	C	A	Nonsynonymous	gaG/gaT
4	2	LOC_Os02g36414	Transporter family protein	Indel	GCGGGCTATTTTTTTTTCTCTCTGATT TGCTGAATTGCTGCTGCGG	-	47 bp deletion in 5'-UTR	-
		LOC_Os02g36440	Transporter family protein	SNP	A	C	Nonsynonymous	gAg/gCg
				SNP	G	T	Nonsynonymous	Ggc/Tgc
		LOC_Os02g36450	Transporter family protein	SNP	A	G	Nonsynonymous	gAc/gGc
				SNP	T	G	Nonsynonymous	gTc/gGc
				SNP	A	G	Nonsynonymous	Agc/Ggc
				SNP	A	G	Nonsynonymous	Atg/Gtg
				SNP	G	C	Nonsynonymous	Gtc/Ctc
5	2	LOC_Os02g46990	AAA-type ATPase family protein	SNP	C	G	Nonsynonymous	Gcg/Ccg
				SNP	T	C	Nonsynonymous	Ttc/Ctc
6	3	LOC_Os03g02380	Major facilitator superfamily domain-containing protein (OsCd1)	SNP	T	A	Nonsynonymous	gTt/gAt
7	3	LOC_Os03g24860	Transporter family protein	SNP	A	G	Nonsynonymous	gTc/gCc
				SNP	A	G	Nonsynonymous	Tgt/Cgt
		LOC_Os03g24870	Transporter family protein	SNP	T	C	Nonsynonymous	Aat/Gat
8	3	LOC_Os03g26650	Heavy metal-associated domain containing protein	Indel	-		9 bp insertion	-/CAAGAACAG
		LOC_Os03g27040	Heavy metal-associated domain containing protein	SNP	T	C	Nonsynonymous	cAc/cGc
9	3	LOC_Os03g27960	Sodium calcium exchanger protein	Indel	-	GCT	3 bp insertion	GCT/-
				Indel	AAC	-	3 bp deletion	-/ACC
		LOC_Os03g28120	Potassium channel protein	SNP	A	T	Nonsynonymous	GtC/GaC
				SNP	C	T	Nonsynonymous	gTC/aTC
				SNP	C	T	Nonsynonymous	CgG/CaG
				SNP	C	T	Nonsynonymous	gAG/aAG
10	3	LOC_Os03g29850	Metal cation transporter	Indel	AACAA	-	5 bp deletion in promoter	-
11	3	LOC_Os03g29920	Heavy metal transport/detoxification protein domain containing protein	SNP	C	G	Nonsynonymous	gCA/gGC
				SNP	A	C	Nonsynonymous	gCA/gGC
12	3	LOC_Os03g61290	Cation/proton antiporter	SNP	A	C	Nonsynonymous	caA/caC
		LOC_Os03g62270	MATE efflux family protein	SNP	A	G	Nonsynonymous	gAc/gGc
				SNP	T	C	Nonsynonymous	gTg/gCg
				SNP	G	A	Nonsynonymous	Gac/Aac
				SNP	G	A	Nonsynonymous	Gcc/Acc
13	7	LOC_Os07g12900	Heavy metal-transporting P1B type ATPase (OsHMA3)	SNP	A	G	Nonsynonymous	gTg/gCg
				SNP	A	G	Nonsynonymous	Tgc/Cgc
				SNP	T	C	Nonsynonymous	Agc/Ggc
				SNP	C	T	Nonsynonymous	Gtc/Atc
				SNP	A	T	Nonsynonymous	ttT/ttA

QTL cluster	Chromosome	Candidate gene		Candidate gene sequence variation				
		ID	Annotation	Variation type	LM	TQ	Effect	Codon change
14	7	LOC_Os07g15370	Natural resistance associated macrophage protein 5 (OsNRAMP5)	Indel	C	-	1 bp delection in 5'-UTR	-
				Indel	CTCTCTCTCTCTCTCTCTCT	-	20 bp deletion in intron near the splicing site	-
15	11	LOC_Os11g34350	ATP-binding cassette sub-family E member (OsABCE2)	SNP	G	A	Nonsynonymous	Gtg/Atg
